# Steric Pruning Unlocks Hierarchical Structuring, Thermochromism, C‐H/O Activation, and 6‐electron Redox Transmetalation in Planar Bismuth Triamides

**DOI:** 10.1002/anie.202518367

**Published:** 2025-11-17

**Authors:** William P. Howlett, Tyler J. Hannah, Lars J. C. van der Zee, J. Chris Slootweg, Jason D. Masuda, Saurabh S. Chitnis

**Affiliations:** ^1^ Department of Chemistry Dalhousie University 6243 Alumni Crescent Halifax NS B3H 4R2 Canada; ^2^ Department of Chemistry University of Victoria 3800 Finnerty Road Victoria BC V8P 5C2 Canada; ^3^ Van ’t Hoff Institute for Molecular Sciences University of Amsterdam PO box 94157 GD Amsterdam 1090 The Netherlands; ^4^ Department of Chemistry Saint Mary's University 923 Robie Street Halifax NS B3H 3C3 Canada

**Keywords:** Bismuth, Pincer ligands, Spectroscopy, Thermochromism, Tungsten

## Abstract

Planar bismuth triamides with tunable stereoelectronics offer a versatile platform for studying the effects of geometric constraints in p‐block elements. Here we show that exploring the low steric bulk regime in these compounds unlocks categorically new phenomena such as epitaxial hierarchical ordering, thermochromism, C─H/O activation, and 6‐electron pincer‐to‐pincer redox transmetalation. These behaviors are not observed in sterically encumbered derivatives, emphasizing the role of steric modulation in unlocking new phenomena in geometrically‐constrained main group complexes.

## Graphical abstract

Reducing steric bulk unlocks remarkable new physical phenomena such as epitaxial and hierarchical ordering, thermochromism, C─H/O activation, and multi‐electron redox transmetalation in planar bismuth triamides. These have all been hidden by the classical use of bulky substituents in this area. These results reveal exciting new properties for geometrically deformed compounds while dispelling the innocence of commonly used W(0) Lewis acids.

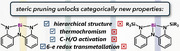

The geometry of p‐block molecules is generally well‐predicted by valence shell electron pair repulsion (VSEPR) theory based on analysis of bonding and lone electron‐pairs around a central atom. The model assumes that substituents can move freely to minimize Pauli repulsion and maximize covalent bonding. While this is true for untethered groups, it is not a given when substituents are interconnected. Such a scenario can be realized via the use of multidentate pincer ligands, which is emerging as a valuable strategy for eliciting new structural diversity, electronic environments, reactivity, and catalytic potential from main group systems.^[^ ^1^, ^2^, ^3^, ^4^, ^5^, ^6^ ^]^

We have been exploring pincer‐coordinated bismuth triamides whose planar geometries show significant deviation from the classically pyramidal geometry of BiR_3_ compounds.^[^ ^7^, ^8^ ^]^ Planarity exposes a vacant Bi 6p orbital perpendicular to the molecular plane and this orbital can either generate metal‐centered Lewis acidity (**A**) or, given its alignment with the ligand π‐system, form conjugated 18‐π compounds (**A′**) that show metal‐centered Lewis basicity via limiting resonance forms like **A″**.^[^ ^9^, ^10^, ^11^, ^12^, ^13^, ^14^ ^]^ The study of these ambiphilic compounds so far has focused primarily on electronic tuning,^[^ ^9^, ^15^, ^16^, ^17^ ^]^ leading to applications in polymerization catalysis,^[^ ^16^ ^]^ heterobimetallic coordination,^[^ ^18^ ^]^ oxygen atom transfer catalysis,^[^ ^19^ ^]^ radical reactivity,^[^ ^20^ ^]^ and predictions of metallopolymer synthesis.^[^ ^21^ ^]^ These results form a small part of the larger renaissance in molecular bismuth chemistry in recent years, which is revealing surprising new examples of stoichiometric and catalytic reactivity at stereo‐electronically tuned bismuth complexes (Figure 1).^[^ ^22^, ^23^, ^24^, ^25^, ^26^, ^27^, ^28^, ^29^, ^30^, ^31^, ^32^, ^33^, ^34^, ^35^, ^36^, ^37^, ^38^, ^39^, ^40^ ^]^

**Figure 1:**
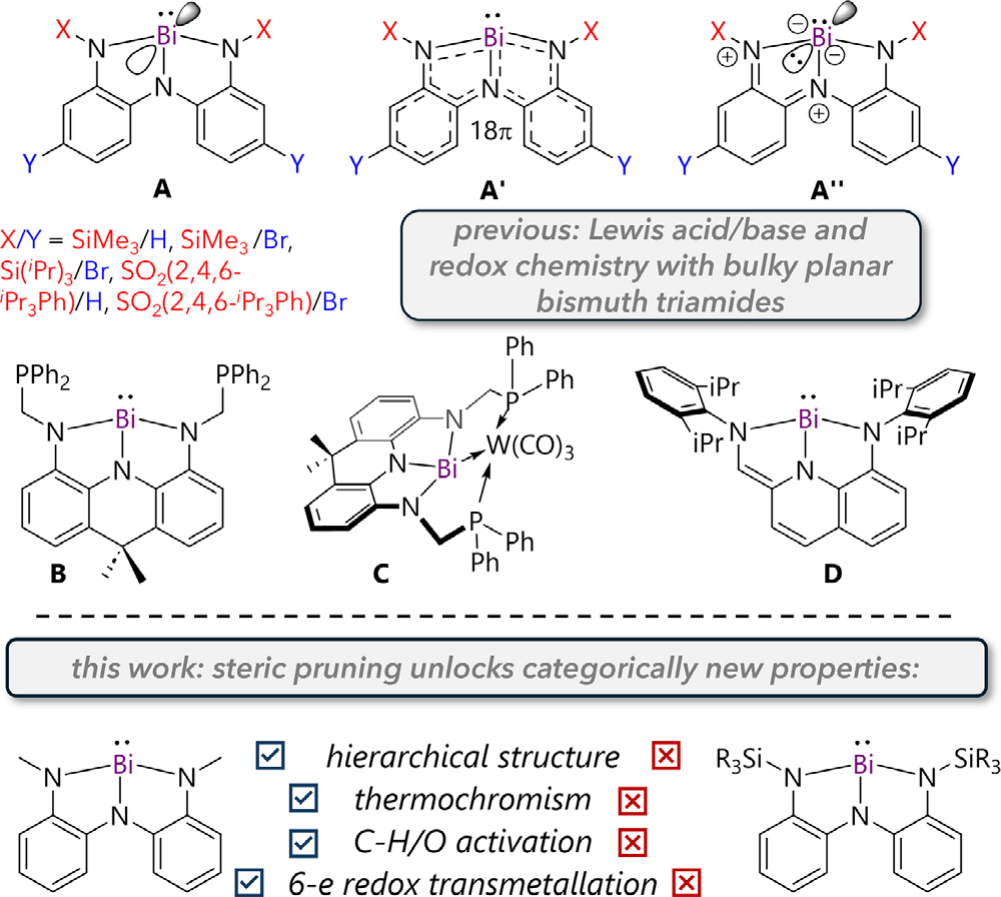
Previously explored and newly‐unlocked properties for planar bismuth triamides.

To date, nearly all derivatives of **A** have employed large flanking groups (N‐SiMe_3_ or larger), which attenuate metal‐centered reactivity.^[^ ^41^ ^]^ For example, only coordination to small Lewis bases (e.g., pyridine N‐oxide, phosphine oxide, dimethylamine) to the metal has been possible. At the other end of the spectrum, metal‐centered Lewis basicity was not evidenced until the recent synthesis of Abbenseth's acridane bismuth triamide **B**.^[^ ^18^ ^]^ In this compound the slightly smaller *N*‐substituent and the phosphine ligands enable Bi→W coordination (**C**), illustrating the ambiphilicity postulated by theoretical analyses.^[^ ^10^ ^]^ Recently, Hwang probed the unique electronic structure of a bulky N‐Dipp (Dipp = 2,6‐diisopropylphenyl) substituted planar bismuth triamide **D** and applied it towards oxygen atom transfer catalysis.^[^ ^19^ ^]^ Inspired by the widening scope of applications for such species and noting that only the high steric bulk regime has been probed so far, we hypothesized that an example with small substituents would reveal properties that were hitherto concealed by steric hindrance. Accordingly, here we show the emergence of unique hierarchical structuring, thermochromism, C─H/O bond activation, and six‐electron pincer‐to‐pincer redox transmetalation in a minimally‐shielded planar bismuth triamide.

Triamines **1a** or **1b**, bearing N─Me or N─Si(*^i^* Pr)_3_ groups, respectively were combined with Bi(NMe_2_)_3_ at ambient temperature to form complexes **2a**,**b** with loss of HNMe_2_ in 80%–95% yields (Figure 2a). The calculated %*V*_bur_ values of **2a** (43%) and **2b** (62%) and steric maps (Figure 2b) show the starkly different extents of crowding around bismuth, while their frontier molecular orbital energies and localizations are similar (Figure 2c), indicating minimal electronic variation. Thus, comparison of the two compounds isolates the role of steric hindrance. Notably, the %*V*_bur_ value for **B** (60%) and **D** (55%) are also large compared to **2a**, emphasizing the unprecedented steric environment of the latter.

**Figure 2:**
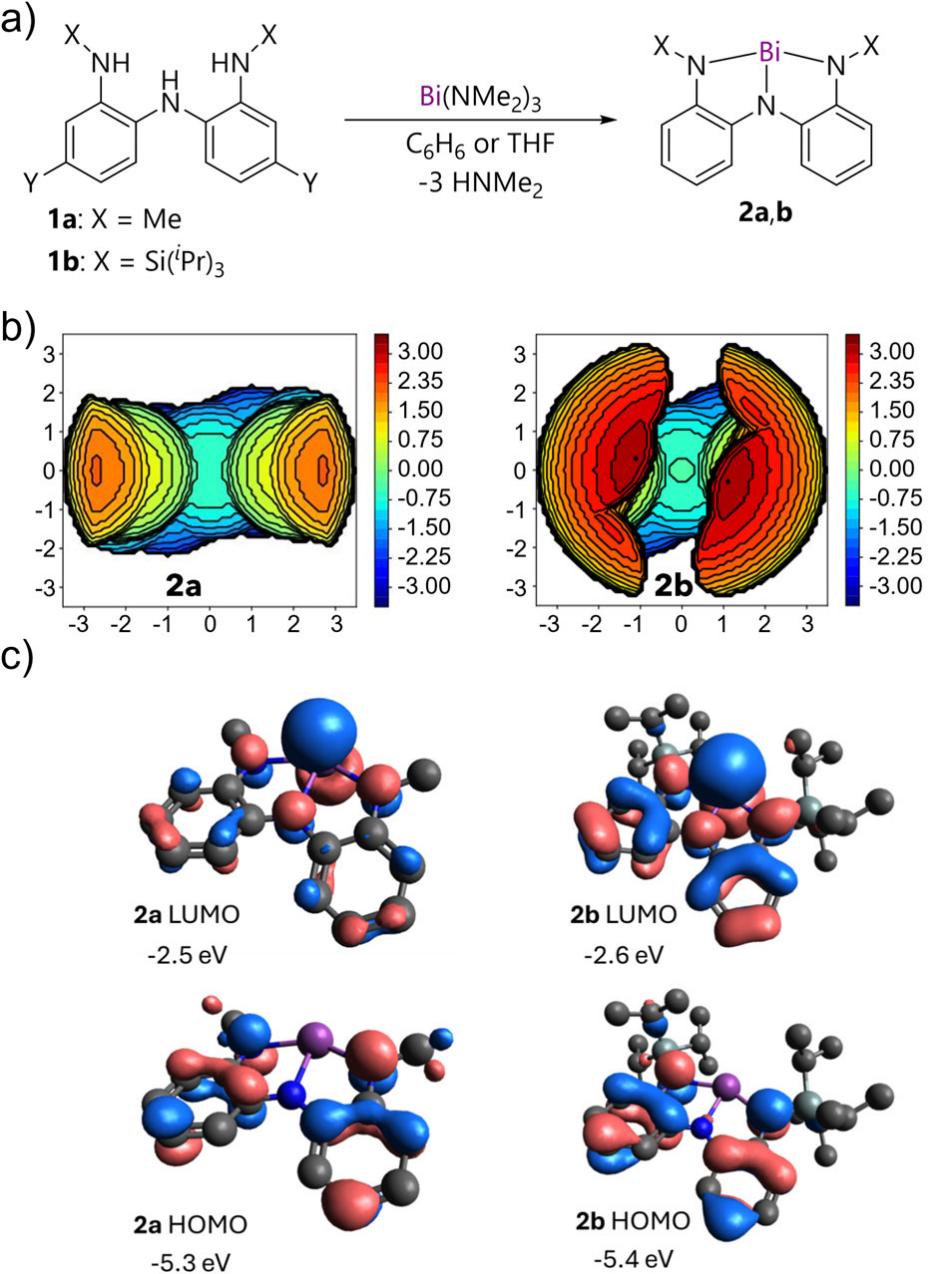
a) Synthesis of compounds **2a, b**. b) SambVca steric maps for compounds **2a** (%*V*_bur_ = 43%) and **2b** (%*V*_bur_ = 62%). Axes and color show distance in Å representing the distribution of steric bulk relative to the plane containing the bismuth atom. c) Frontier molecular orbitals of **2a, b** at the PBE0/def2‐QZVP + D3BJ level.

An X‐ray diffraction experiment on reddish‐brown crystals (Figure S80) revealed that **2a** forms centrosymmetric dimers in the solid state (Figure 3a, **2a′**). Each bismuth centre in the dimer is best described as being pyramidal, with a short, electron‐sharing Bi─N bond [2.329(2) Å] where a vacant Bi 6p orbital would be present in the monomer, and a long, dative N→Bi interaction [2.496(2) Å] (Figure S100). Such dimers have been observed for derivatives with electron withdrawing groups in the ligand previously, which was ascribed to increased metal‐centered Lewis acidity based on analyses of orbital interactions.^[^ ^9^, ^16^ ^]^ But the formation of **2a′** here shows dimerization is the default behavior for planar bismuth triamides even without electronegative substituents, except when sterically precluded.^[^ ^42^, ^43^, ^44^ ^]^

**Figure 3:**
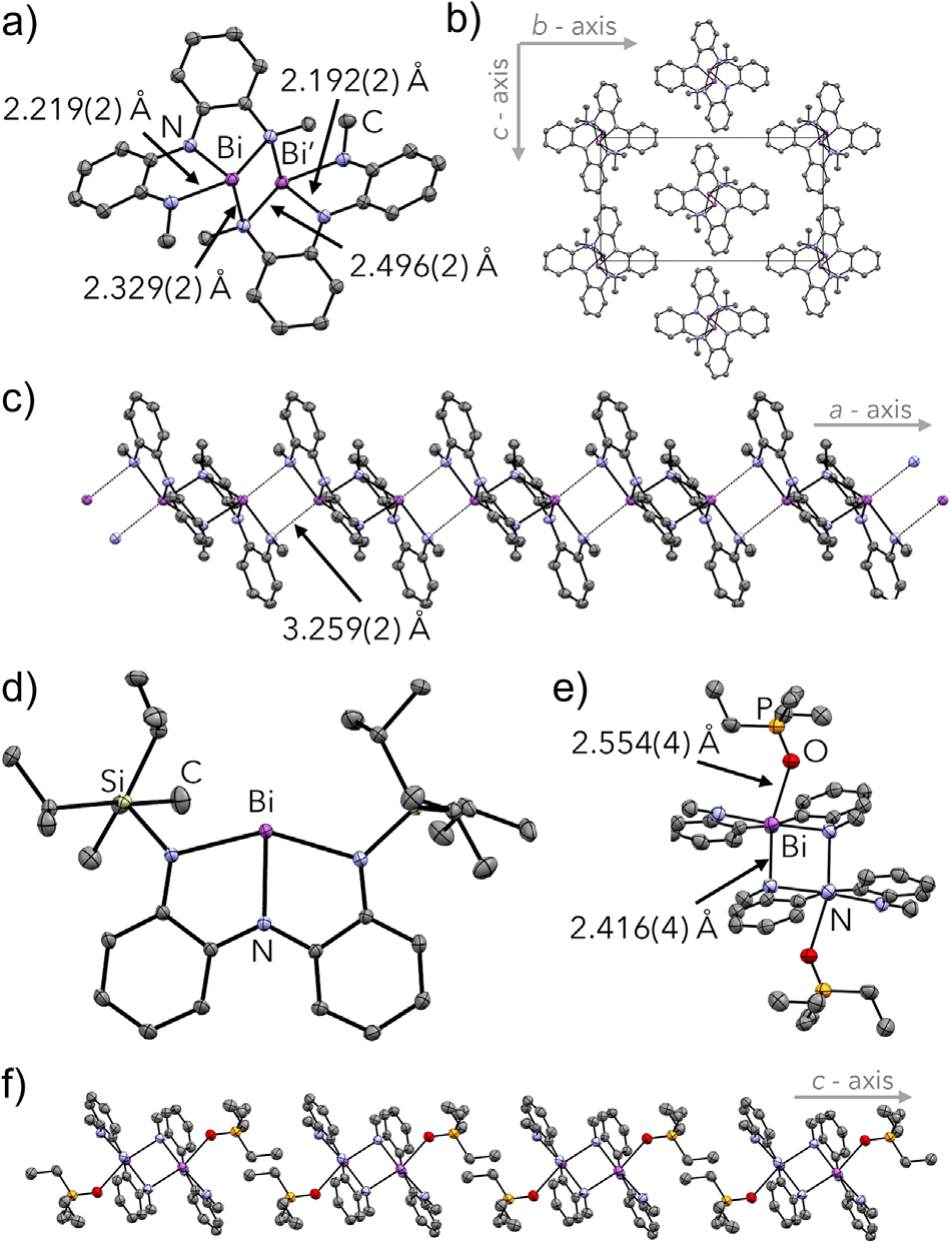
a) Molecular structure of **2a′** in the solid state. b) View down the a‐axis of **2a′** in the *bc* plane. c) The “polymer of dimer” structure of **2a′** along the *a*‐axis. d) Molecular structure of **2b** in the solid state. e) Molecular structure of **3** in the solid state showing selected bond lengths. f) Packing of **3** along the *c*‐axis.^[^ ^45^ ^]^

Notably, **2a′** further associates with equivalent dimers via long Bi—N interactions [3.259(2) Å], producing a “polymer of dimers” in the longitudinal *a*‐axis with hexagonal packing and minimal interactions in the transverse *bc*‐plane (Figure 3b,c). This additional level of structuring has not been observed previously and may explain the poor solubility of **2a** in non‐coordinating solvents. The structure of bulkier, planar, and monomeric derivative **2b** is much simpler by comparison (Figure 3d). It shows isotropic intermolecular C─H—H─C and C─H—π interactions interpreted as being due to London dispersion and it is highly soluble even in pentanes. Thus, pruning the steric bulk reveals a new hierarchical and highly‐oriented solid‐state structure, with consequences expected for behavior in solution.

The ^1^H NMR spectra in of **2a** and **2b** (in THF‐*d8* and benzene‐*d6*, respectively) show *C*_2v_ symmetry at 298 K, with four resonances of equal intensity being observed in the aromatic region. The UV‐vis spectra of both in THF (Figures S29 and S30) show a prominent band between 600‐640 nm and a second, less prominent one in the 500‐600 nm range. These bands are consistent with the blue color of the compounds in solution. Time‐dependent density functional theory (TD‐DFT) calculations predict absorbances for dimer **2a'** at significantly lower wavelengths and weaker intensity since the low‐lying 6p LUMO that is available at Bi in the monomer is partially quenched upon dimerization, leaving only higher energy acceptor orbitals for electronic transitions (Figure S98). On the other hand, the calculations show that strong absorbances in the 500‐650 nm range are expected for planar, monomeric compounds corresponding to a HOMO→LUMO and HOMO‐1→LUMO excitation (Figures S97 and S98), indicating the presence of monomeric **2a** in THF.

It was therefore surprising that the reaction between **2a** and excess OPEt_3_ in THF gave bimetallic **3** (Figure 1e), rather than the expected monometallic **2a**•(OPEt_3_)_2_. The structure of **3** is derived simply by replacing the inter‐dimer interactions with OPEt_3_ molecules in the longitudinal axis (cf. Figure 1c and f). Given that spectroscopy supports the presence of monomers but reactivity with OPEt_3_ implies the presence of dimers, we conclude that in THF, planar, monomeric, and blue **2a** exists in equilibrium with bent, dimeric and weakly‐colored **2a′**. In other words, the solid‐state “polymer‐of‐dimers” is dissociated in THF, but a monomer‐dimer equilibrium nevertheless persists (Figure 4a).

**Figure 4:**
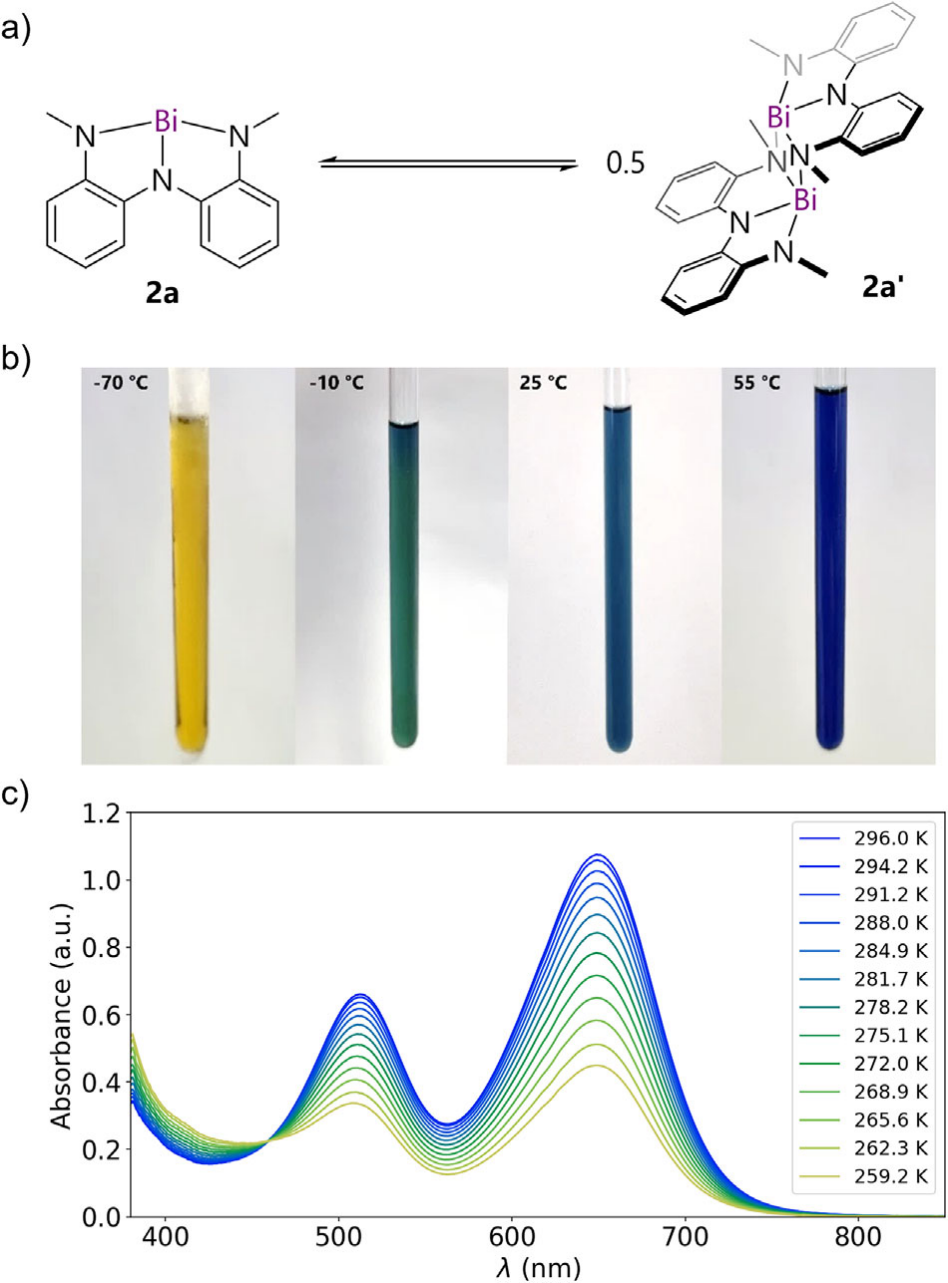
a) Proposed equilibrium between dimer **2a′** and monomer **2a**. b) Thermochromism of **2a**/**2a′** in THF. c) Temperature depended UV‐vis spectra in THF showing the conversion of **2a** into **2a′** upon cooling.

Since different colors are predicted for the monomer and dimer, this equilibrium offers a basis for thermochromism if the associated free energy change is modest. Consistently, blue THF solutions of **2a** at ambient temperature turn yellow reversibly upon cooling (Figure 4b, see video S1). Temperature dependent UV‐vis analysis confirms the disappearance of the two characteristic absorption bands of monomer **2a** upon cooling (Figure 4c), and appearance of a new band below 400 nm for dimer **2a'**. Furthermore, an isosbestic point at 459 nm indicates that the dimer indeed absorbs only at lower wavelengths than the monomer. Fitting of the peak absorption at 649 nm over the measured temperature range yields the dimerization Gibbs energy (Δ*G*_dim,298 K_) as −14.4 ± 0.3 kJ/mol (*K*_eq_ = 330 M^−1^ for dimerization) with an enthalpy and entropy of dimerization of −65.8 ± 3.3 kJ/mol and −172 ± 12 J/mol•K, respectively (Table S1).^[^ ^46^ ^]^ Similar results were obtained in toluene (Table S2). Further evidence of dimer formation at low temperature in solution was detected by ^1^H NMR spectroscopy in THF‐*d* *8*. Four aromatic resonances and a single N─Me resonance are observed at 298 K, consistent with a dimer that exhibits rapid side‐to‐side motion that renders the N─Me groups equivalent. But upon cooling to 188 K, eight aromatic resonances and two resonances for the N─Me groups are observed, matching the reduced symmetry of dimer **2a′**. Eyring analysis indicated a small barrier of 42.5 ± 5.6 kJ/mol for the dynamic motion that makes the two sets of resonances equivalent on the NMR timescale at 298 K (Figure S42). Thus, NMR spectroscopic evidence supports a monomer‐dimer equilibrium and connects it unambiguously to the observed thermochromism. Crucially, no thermochromism or evidence of dimerization was observed for **2b**, highlighting the significance of steric control in unlocking this photophysical phenomenon.

As neutral OPEt_3_ failed to dissociate dimeric **2a′**, we hypothesized that an anionic oxide may be more suitable due to its higher donor strength and introduction of a Coulombic barrier to dimerization. Surprisingly, the combination of **2a** with KO*^t^* Bu gave tetrametallic complex **4** (Figure 5a), whose structure shows μ_3_‐oxygen atoms bridging four bismuth centers and no remaining *^t^* butyl groups. Two potassium ions are closely associated with aryl rings and shrouded in THF molecules. We tentatively propose that following coordination of *^t^* BuO^−^ to bismuth in **2a′**, an intramolecular β─CH deprotonation of the *^t^* butyl group occurs by an amide arm to eliminate isobutene. A related mechanism for β─CH deprotonation from Bi‐O*^t^* Bu groups by an added equivalent of *^t^* BuO^−^ has been reported by Veith.^[^ ^47^ ^]^ The resulting Bi─O─Bi bridged intermediate could, upon dimerization, yield **4**. While free isobutene could not be detected in the reaction mixture, polyisobutylene was detected by ^1^H NMR and mass spectrometry, suggesting rapid polymerization under the reaction conditions (Figure S11, S27, and S28). Reactions involving KOMe and KOPh, which lack β‐CH groups showed no evidence of compound **4**, further supporting the proposed reaction pathway (Figures S17 and S19). From the reaction with KOMe, compound **7** which is a coordination polymer with now elongated Bi—N contacts [2.716(5) Å], was structurally characterized (Figure 5b), supporting the alkoxide coordination proposed as the first step in the sequence leading to formation of **4**. The bulkier derivative **2b** also showed no evidence of β─CH deprotonation, yielding instead the simple 1:1 adduct **5** (Figure 5c). Thus, reducing the steric bulk unlocks C─H/O bond activation at bismuth following coordination, when a labile *β*─CH is present.

**Figure 5:**
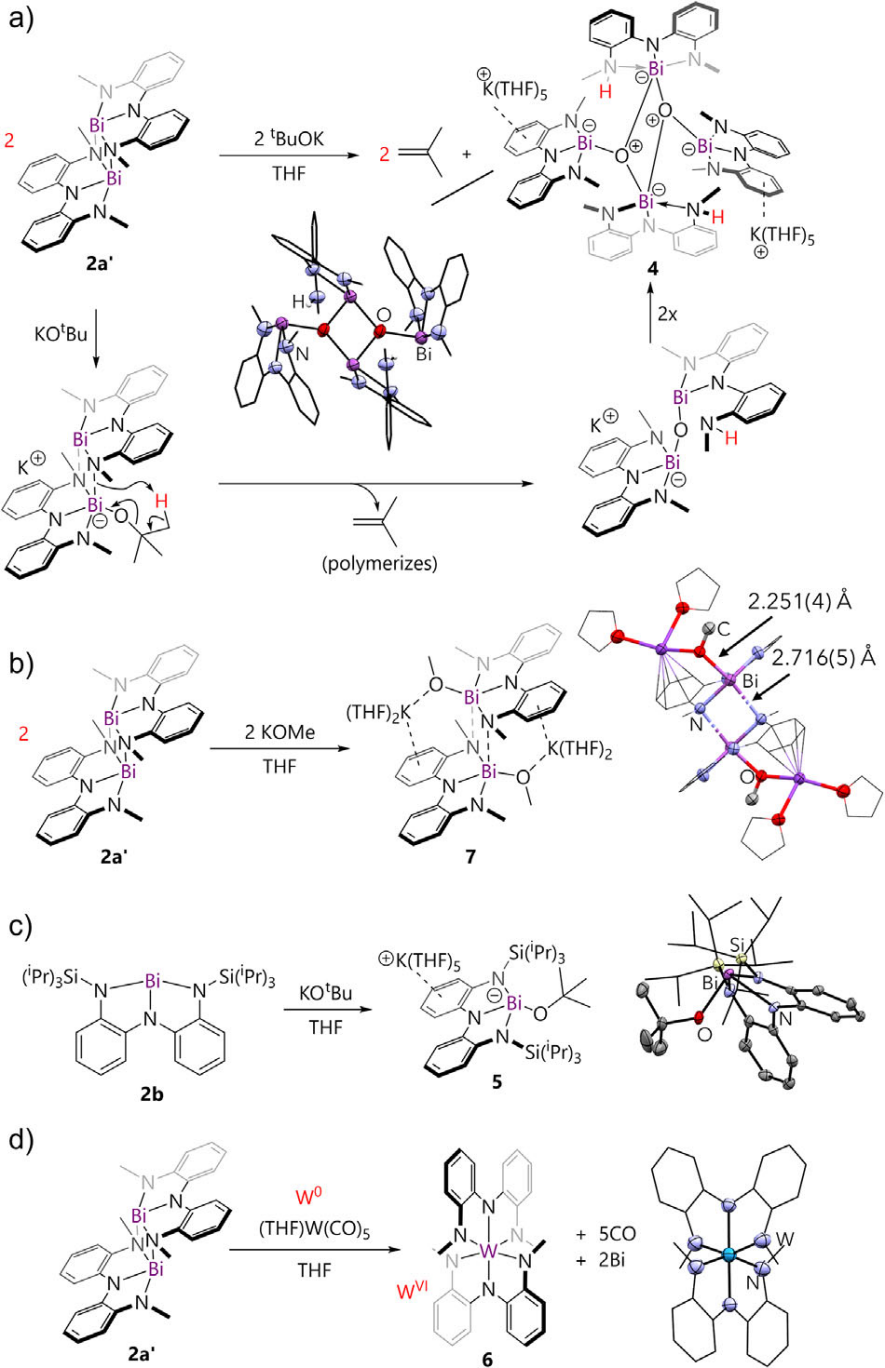
a) Reaction of **2a′** with KO*^t^* Bu to give **4**. Molecular structure of the dianionic portion of **4** is shown, with carbon atoms shown in wireframe and hydrogen atoms omitted for clarity. The full structure is shown in the Supporting Information. b) Reaction of **2a′** with KO*^t^* Bu to give **7** and its structure. Extended polymeric structure is shown in Supporting Information. c) Reaction of **2b** with KO*^t^* Bu to give **5** and structure of the anion in the latter. d) Reaction of **2a** with W(CO)_5_ and structure of the resulting hexa‐amide **6**.

To map the other end of ambiphilic reactivity, we considered the effect of steric variation on metal‐centered Lewis basicity. The W(0) compound W(CO)_5_(THF) is widely‐employed as a Lewis acid for isolation of otherwise reactive electron‐rich species, including soft bismuth donors (e.g., **C**).^[^ ^18^, ^48^, ^49^, ^50^ ^]^ We therefore combined **2a** with W(CO)_5_(THF), but in contrast to the expected 1:1 adduct, the W(VI) compound **6** was isolated in quantitative NMR yield and 84% isolated yield (Figure 5d). A bismuth mirror was also deposited. This outcome represents a remarkable six‐electron pincer‐to‐pincer redox transmetalation giving only the third example of a tungsten hexa‐amide.^[^ ^51^, ^52^, ^53^, ^54^ ^]^ Moreover, of the ca. 1000 reported reactions of W(CO)_5_(THF) or W(CO)_5_(NCMe) catalogued within ca. 900 reports in the Chemical Abstract Service, all but five show simple ligand substitution processes. The five showing redox chemistry represent W(0)→W(I),^[^ ^55^ ^]^ W(0)→W(II),^[^ ^56^, ^57^ ^]^ W(0)→W(III),^[^ ^58^ ^]^ or W(0)→W(IV) oxidations,^[^ ^59^ ^]^ and these involve electronegative chalcogens. By comparison, the six‐electron W(0)→W(VI) transformation observed here is unprecedented and particularly surprising given that an electron‐rich triamide ligand rather than oxidizing chalcogens or halogens are involved. In related work, Dostàl has previously shown pincer‐to‐pincer transfer from Sb(I) to give Ir(III) and Rh(III) centres^[^ ^60^ ^]^ and recently Abbenseth showed oxidative insertion of a Mo(0) complex into a planar phosphorus triamide.^[^ ^61^ ^]^ As no intermediates were detected spectroscopically in the formation of **6**, detailed mechanistic discussion is not possible at this stage. In contrast to **2a**, no interaction was observed between **2b** and W(CO)_5_(THF), suggesting that the coordination‐redox sequence observed in the former bismuth complex is frustrated in the latter by the steric bulk around the metal.

In summary, probing the as‐yet unexplored low steric bulk regime of planar bismuth triamides reveals i) a unique hierarchically‐organized structure in the solid state, ii) unexpected physical phenomena such as thermochromism, iii) rare C‐H/O bond activation, and iv) an unprecedented six‐electron pincer‐to‐pincer redox transmetalation from bismuth to tungsten. These properties have hitherto been obscured by steric bulk^[^ ^62^ ^]^ and their detection reveals exciting new vistas in the study of an emerging compound class. Beyond insights specific to bismuth chemistry, these results also illustrate how electron‐rich pincer ligands, which are known to be super‐reductants,^[^ ^11^ ^]^ can counterintuitively behave as oxidants under strong metal‐ligand coupling conditions (e.g., as captured by limiting resonance form **A″**, Figure 1), while also dispelling the oft‐presumed innocence of widely‐used W(0) Lewis acids, and the accepted robustness of pincer coordination. Given the widespread use of electron‐rich pincers as ancillary ligands and low oxidation state transition metal centers as soft Lewis acids, these findings may prove instructive for both transition metal and main group coordination chemistry. Future studies will explore applications of the observed stoichiometric reactivity in catalysis and develop new solid‐state materials leveraging the long‐range epitaxial ordering observed in **2a**.

## Supporting Information

The authors have cited additional references within the Supporting Information.^[^ ^7^, ^43^, ^46^, ^63^, ^64^, ^65^, ^66^, ^67^, ^68^, ^69^, ^70^, ^71^, ^72^, ^73^ ^]^

## Conflict of Interests

The authors declare no conflict of interest.

## Supporting information



Supporting Information

Supporting Information

Supporting Information

## Data Availability

The data that support the findings of this study are available in the supplementary material of this article.
