# Supplementary material for: Steric Pruning Unlocks Hierarchical Structuring, Thermochromism, C‐H/O Activation, and 6‐electron Redox Transmetalation in Planar Bismuth Triamides
Source: Angew Chem Int Ed Engl. 2025 Nov 17;65(1):e18367. doi: 10.1002/anie.202518367 (PMC12759222; doi:10.1002/anie.202518367)
Supplement: Supplementary file 1 — Supporting Information [file ANIE-65-e18367-s001.pdf]

Supplementary Information

Table of Contents

Supplementary Information 1

1 Experimental Procedures 2

2 Syntheses and Characterization Data 5

3 Spectral Data for New Compounds 15

3.1 NMR Spectral Data 15

3.2 IR Spectral Data 33

3.3 Mass Spectrometry Data 37

3.4 UV-vis Spectral Data 38

4 Determination of Thermodynamic and Kinetic Parameters of **2a** 41

4.1 Variable Temperature UV-vis spectroscopy 41

4.2 Variable Temperature Nuclear Magnetic Resonance Spectroscopy 46

5 X-ray Crystallography Data 84

5.1 Crystal Structures 85

6 Computational Methods 92

6.1 Determination of Percent Buried Volume 92

6.2 Percent Buried Volume Data 92

6.3 Calculated UV-Vis spectra and assignments 95

6.4 Calculated frontier MOs for **2a** and **2b** 96

6.5 Cartesian coordinates of calculated structures 96

6.6 Description of **2a** Bonding as a Dimer 99

7 References 99

1 Experimental Procedures

General

All manipulations were performed under dry nitrogen atmosphere using standard Schlenk techniques or inside an LC Technology Solutions, Inc. glovebox. Solvents were distilled from Na/benzophenone stills. Methanol was degassed with nitrogen and stored over activated 3 Å sieves. Benzene-*d6* was freeze-pump-thawed twice and stored over activated 3 Å sieves. Tetrahydrofuran-*d8* (THF-*d8*) was vacuum distilled after refluxing over K/benzophenone and stored over activated 3 Å sieves. Glassware used for reactions was baked in an oven at 150 °C for 12 hours before use and assembled inside an air-free glove box or under nitrogen flow. Silanized glass was prepared by treating with trimethylsilyl chloride (Me_3SiCl), rinsing with distilled water, acetone and baked in an oven at 150°C for at least 1 hour. Electro-spray ionization (ESI) and atmospheric pressure chemical ionization (APCI) mass spectra were obtained on a Bruker micrOTOF MS instrument. UV-vis characterization spectra were obtained on a Buker Tensor 27 FT-IR spectrometer. Melting points (m.p.) were measured using a MEL-TEMP II melting point apparatus. Samples were loaded into glass capillaries in a glove box under dry nitrogen atmosphere and sealed from the atmosphere with silicone grease.

Solution NMR

^{1}H, $^{13}C\{^{1}H\}$ and ^{19}F NMR spectra were collected on a Bruker Avance 500 MHz spectrometer equipped with a cryoprobe. $^{31}P\{^{1}H\}$ NMR spectra were collected on a Bruker Avance 400 MHz spectrometer. ^{1}H and ^{13}C NMR spectra are internally referenced to residual solvent, ^{31}P and ^{19}F spectra are referenced to external standards of H_3PO_4 and trichlorofluoromethane, respectively. Variable temperature NMR spectra were obtained on a Bruker Avance 300 MHz spectrometer in sealed J. Young NMR tubes and THF-*d8* solvent.

Starting Materials and Commercial Reagents

S1 and **S2** were sourced from Oakwood Chemicals. $Bi(NMe_2)$,[1] **S3**,[2] **S4**,[2] and **1a**,[3] were synthesized according to literature procedures. $BiCl_3$ and $W(CO)_6$ were obtained from Oakwood Chemicals and purified *via* sublimation under vacuum prior to use. Lithium dimethylamide and potassium *tert*-butoxide were purchased from Sigma-Aldrich and used as delivered. Potassium methoxide was prepared from potassium metal and anhydrous methanol, then dried at 110°C under vacuum for 48 hours. Experiments with potassium methoxide were repeated with material purchased from Sigma-Aldrich and used as delivered. Potassium phenoxide was prepared from potassium hydride and phenol that was sublimed prior to use.

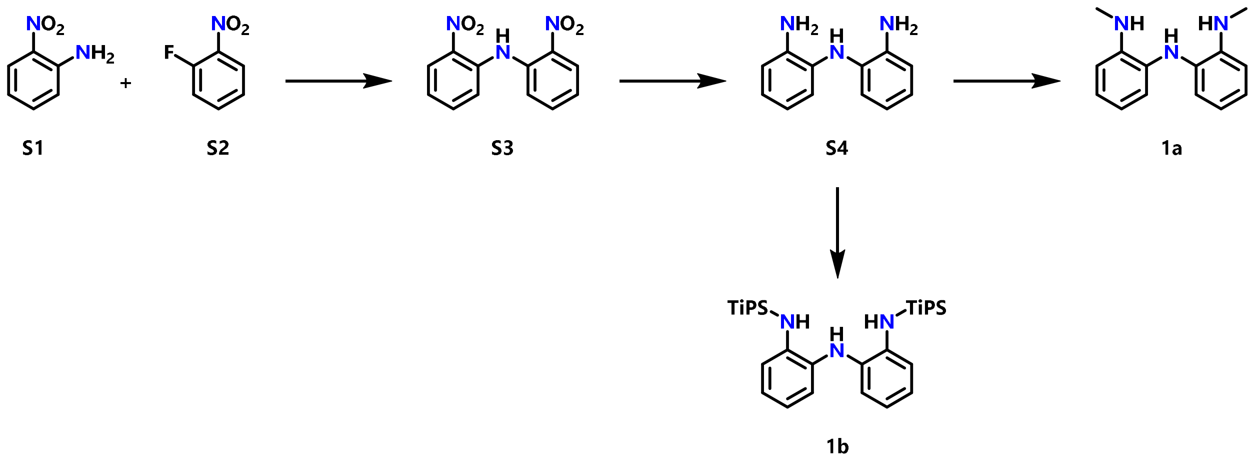

Scheme S1.

Elemental Analysis

Elemental analyses were performed on samples packed into tin weigh boats in air-free glove boxes. Combustion analysis was performed using the CHN/S mode on an Elementar Unicube instrument. Journal requirements of ±0.40% accuracy for all elements have been critically re-evaluated in 2022.[4] Due to the high air-sensitivity of the compounds, elemental analysis results could not be obtained in all cases. However, spectroscopic purity is indicated by clean NMR spectra. Note also that no NMR-silent byproducts are expected in most cases since salt-elimination reactions were not used.

Variable Temperature UV-vis Spectroscopy

All manipulations were carried out under an atmosphere of purified nitrogen using standard glovebox techniques. THF was distilled from sodium/benzophenone and stored over activated 3Å molecular sieves. Toluene was distilled from sodium/benzophenone and stored over a sodium mirror. Variable temperature UV-vis spectroscopy was measured on a HP (Agilent) 8453 single beam spectrometer equipped with a variable temperature cuvette holder and the temperature was controlled with a Lauda Proline RP890 thermostat filled with Julabo Thermal HY as bath fluid. Room temperature UV-vis spectroscopy was measured at a Shimadzu UV-2600 double beam spectrometer with medium scan speed. For all UV-vis experiments, Hellma 117.100F-QS fluorescence cuvettes sealed with Duran GL14 closed caps were used.

X-ray Crystallography

The selected crystal was attached to the tip of a MicroLoop with Paratone-N oil. Measurements were made on a Bruker D8 VENTURE diffractometer equipped with a PHOTON III CMOS detector using monochromated Mo Kα radiation (λ = 0.71073 Å) from an Incoatec micro-focus sealed tube at 150 K. The initial orientation and unit cell were indexed using a least-squares analysis of the reflections collected from a complete 180° phi-scan with 1° per frame. For data collection, a strategy was calculated to maximize data completeness and multiplicity, in a reasonable amount of time, and then

implemented using the Bruker Apex 4 software suite[5, 6, 7]. The crystal to detector distance was set to 4 cm. Data collection, unit cell refinement, data processing and multi-scan absorption correction were applied using the APEX4[5] software package. The structures were solved using SHELXT[8] and all non-hydrogen atoms were refined anisotropically with SHELXL[9] using OLEX2[10] graphical user interface. Unless otherwise noted, all hydrogen atom positions were idealized and ride on the atom to which they were attached. The final refinement included anisotropic displacement factors on all non-hydrogen atoms. For compound **4**, attempts to model disorder for each of the four THF molecules attached to potassium were unsuccessful, thus a series of restraints were applied to give reasonable models for the THF. Compound 6 was found to be a two component, non-merohedral twin using Cell_Now with the second domain rotated 180 degrees. Upon refinement, the ratio of the two components was 95:5. The tridentate ligands were modeled with a two component (50:50) disorder where the disordered ligands are orthogonal to one another. There is also one co-crystallized THF molecule with 50% occupancy that can be associated with one of the twin components; the other twin component is too close in-space for the THF to be present with that component.

2 Syntheses and Characterization Data

*2.1.1 Synthesis of **1b***

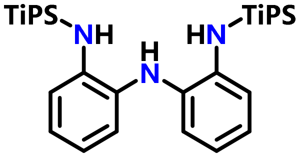

In a 100 mL bomb flask, 3.893 mL (2.1 eq., 14.5 mmol) of triisopropylsilyl triflate were added dropwise to a stirring a mixture of bis(2-aminophenyl)amine (**S4**, 1 eq., 1.374 g, 6.9 mmol) and triethylamine (2.5 eq., 2.405 mL, 17.2 mmol) in 25 mL THF. This was then sealed and left to stir for three days, before drying *in vacuo*, a pentane wash, cannula filtration with pentane and again put under vacuum until dry. Small amounts of impurity can lead to a dark red/purple oil that is pure enough to use in the next step, but the impurity can be removed by repeated filtrations in pentane through celite. Drying the purified product *in vacuo* gave 3.351 grams of pure **1b** as a white powder (6.5 mmol). Yield: 94.9%.

^{1}H NMR (300 MHz, benzene-*d6*, ppm): δ 6.96 (d, *J* = 3.6 Hz, 4H), 6.69 (d, *J* = 2.3 Hz, 4H), 4.61 (s, 1H), 3.69 (s, 2H), 1.15 (d, *J* = 6.3 Hz, 6H), 1.06 (d, *J* = 6.5 Hz, 36H).

^{13}C{^{1}H} NMR (70 MHz, benzene-*d6*, ppm): δ 140.80, 132.50, 124.39, 121.86, 118.92, 116.94, 18.73, 12.86.

EA calc'd for $C_{30}H_{53}N_3Si_2$ [%]: C, 70.38; H, 10.44; N, 8.21; **found [%]**: C, 70.39; H, 10.48; N, 7.82.

*2.1.2 Synthesis of **2a***

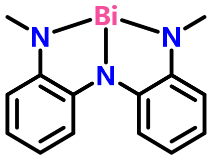

At -30°C, a solution of bismuth tris(dimethylamide) (302.5 mg, 0.89 mmol) in 3 mL of THF was added dropwise to a stirring solution of **1a** (201.5 mg, 0.89 mmol) in 3 mL of THF, then allowed to warm to room temperature and stir overnight. The reaction mixture changed from a red solution to a dark brown suspension over the course of the reaction. This mixture was then dried *in vacuo* and washed with pentane (3x3mL) and dried again to give 305.2 mg (0.70 mmol) of **2a** as a red powder. Yield: 79.5%. Recrystallization in THF yielded crystals suitable for single crystal X-ray diffraction measurement. Crystal data for dimeric **2a** plus solvent ($Bi_2N_6C_{36}H_{44}O_2$): monoclinic, space group $P2_1/c$ (no. 14), a = 7.316(2) Å, b = 20.2186(8) Å, c = 11.3147(5) Å, α = 90°, β = 97.693(2)°, γ = 90°, V = 1658.64(12) Å^3, Z = 2, T = 150.00 K, μ(MoKα) = 10.638^{-1}, D_{calc} = 2.024 g·cm^{-3}, 83356 reflections measured (5.426° ≤ θ ≤ 66.316°). The final R1 was 0.0244 (I > 2σ(I)) and wR2 was 0.0642 (all data). CCDC no. 2459250.

^{1}H NMR (500 MHz, THF-*d8*, ppm): δ 7.75 (d, J = 8.1 Hz, 2H), 6.74 – 6.69 (m, 1H), 6.67 (td, J = 7.4, 1.3 Hz, 1H), 6.58 (ddd, J = 8.4, 6.9, 1.7 Hz, 2H), 4.11 (s, 6H).

^{13}C{^{1}H} NMR (126 MHz, THF-*d8*, ppm): δ 151.09, 148.36, 129.20, 121.14, 118.48, 118.14, 116.80, 35.36.

EA calc'd for $C_{14}H_{14}BiN_3$ [%]: C, 38.81; H, 3.26; N, 9.70; **found [%]**: C, 38.55; H, 3.20; N, 9.48.

IR (cm^{-1}): 729 (s), 777 (w), 809 (w), 838 (w), 902 (vw), 1017 (m), 1034 (w), 1052 (w), 1107 (w), 1119 (w), 1138 (w), 1162 (w), 1189 (w), 1220 (w), 1248 (m), 1259 (m), 1291 (m), 1328 (w), 1417 (w), 1439 (m), 1478 (vs), 1505 (w), 1515 (w), 1557 (vw), 1573 (m), 2789 (w), 2851 (w), 2882 (w), 2914 (vw), 3054 (vw).

Neither ESI-MS nor APCI-MS methods were able to detect the proposed product.

m.p.: 160°C (decomposes).

*2.1.3 Synthesis of **2b***

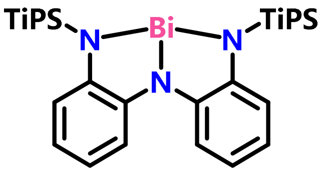

At -30°C, a solution of bismuth tris(dimethylamide) (301.1 mg, 0.59 mmol) in 3 mL of hexanes was added dropwise to a stirring solution of bis(2-(*N*-triisopropylsilyl)aminophenyl)amine (**1b**, 200.3 mg, 0.59 mmol) in 3 mL of hexanes, then allowed to warm to room temperature and stir overnight. The reaction mixture became dark blue immediately upon addition. The solution was dried *in vacuo*, redissolved in pentane, filtered and dried again to give (398.5 mg, 0.56 mmol) of black powder. Yield: 95%. Recrystallization in pentane yielded crystals suitable for single crystal X-ray diffraction measurement. Crystal data for dimeric **2b** ($BiN_3C_{30}H_{50}Si_2$): monoclinic, space group $P2_1/c$ (no. 14), a = 15.7960(5) Å, b = 7.8257(2) Å, c = 25.3258(7) Å, α = 90°, β = 96.0310(10)°, γ = 90°, V = 3113.32(15) Å^3, Z = 4, T = 150.00 K, μ(MoKα) = 5.763^{-1}, D_{calc} = 1.532 g·cm^{-3}, 136828 reflections measured (5.186° ≤ θ ≤ 74.234°). The final R1 was 0.0171 (I > 2σ(I)) and wR2 was 0.0366 (all data). CCDC no. 2459254.

^{1}H NMR (500 MHz, benzene-*d6*, ppm): δ 8.32 (dd, J = 8.5, 1.5 Hz, 2H), 7.50 (dd, J = 8.5, 1.4 Hz, 2H), 6.73 (ddd, J = 8.4, 6.8, 1.4 Hz, 1H), 6.59 (ddd, J = 8.4, 6.8, 1.3 Hz, 1H), 1.63 (h, J = 7.5 Hz, 6H), 1.21 (d, J = 7.6 Hz, 36H).

^{13}C{^{1}H} NMR (126 MHz, benzene-*d6*, ppm): δ 154.67, 152.63, 124.54, 123.49, 123.35, 117.40, 19.24, 19.22, 15.17.

ESI-HRMS (positive ion mode): calculated for [$C_{30}H_{51}BiN_3Si_2$]$^+$ = 718.3420 m/z, **observed** = 718.3437.

m.p.: 162-171°C (decomposes)

2.1.4 Synthesis of **3**

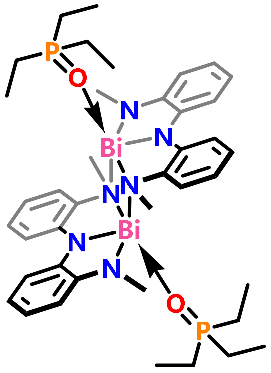

To a solution containing 31.1 mg **2a** (1 eq., 0.07 mmol) in THF was added a solution containing 96.3 mg triethylphosphine oxide (10 eq., 0.72 mmol) in THF and left to react. After 24 hours, the solution was filtered, dried *in vacuo* and washed with pentane. The red powder was then redissolved in THF and recrystallized in a THF/pentane slow vapour diffusion system, giving X-ray quality crystals. The NMR data were gathered before purification, as drying the sample *in vacuo* removes excess triethylphosphine oxide, subsequently reducing the ratio of **2a** to the phosphine oxide upon redissolution and giving a different set of signals. As a result, it is believed that the ^{31}P NMR resonance is drowned out due to the significant excess of triethylphosphine oxide not engaged in coordination. Crystal data for dimeric **3** ($Bi_2N_6C_{40}H_{58}O_2P_2$): monoclinic, space group $P2_1/c$ (no. 14), a = 8.3079(3) Å, b = 19.0371(7) Å, c = 13.0919(4) Å, α = 90°, β = 103.3770(10)°, γ = 90°, V = 2014.42(12) Å^3, Z = 2, T = 150.00 K, μ(MoKα) = 8.846^{-1}, D_{calc} = 1.871 g·cm^{-3}, 51070 reflections measured (5.040° ≤ 2θ ≤ 50.040°). The final R1 was 0.0282 (I > 2σ(I)) and wR2 was 0.0728 (all data).

^{1}H NMR (400 MHz, THF-d_8): δ 7.61 (d, J = 7.7 Hz, 1H), 6.60 (td, J = 7.3, 1.4 Hz, 1H), 6.54 – 6.44 (m, 2H), 3.72 (s, 3H), 1.53 (dq, J = 11.6, 7.7 Hz, tBu), 1.04 (dt, J = 15.5, 7.7 Hz, tBu).

^{31}P NMR (162 MHz, THF-*d8*): δ 45.70.

EA calc'd for $C_{40}H_{58}Bi_2N_6P_2O_2$ [%]: C, 42.34; H, 5.15; N, 7.41; **found [%]**: C, 42.85; H, 5.41; N, 7.14.

Neither ESI-MS nor APCI-MS methods were able to detect the proposed product.

2.1.5 *Synthesis of* ***4***

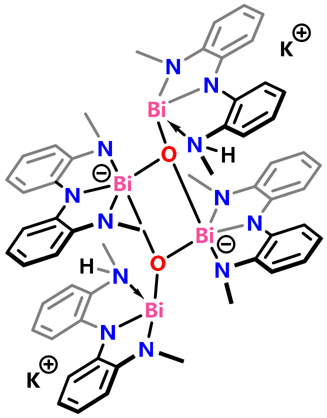

To a stirring mixture of **2a** (117.2 mg, 0.27 mmol) in THF, 13 mg (0.14 mmol) of potassium *tert*-butoxide was added, resulting in an immediate colour change to brown. After letting stir for 15 minutes, the mixture is collected on a glass frit, washed with pentane† and dried *in vacuo* to give 70.2 mg of a red-brown solid. Recrystallization for single crystal X-ray diffraction measurement quality crystals was done by slow vapour diffusion of pentane into THF. Yield: 59%. Crystal data for tetrameric **4** with solvent ($Bi_4N_{12}C_{92}H_{124}K_2O_{11}$): triclinic, space group P-1 (no. 2), a = 11.9318(10) Å, b = 14.6398(13) Å, c = 14.8560(12) Å, α = 77.535(3)°, β = 74.628(3)°, γ = 74.494(3)°, V = 2831.59(4) Å^3, Z = 1, T = 150.00 K, μ(MoKα) = 7.517^{-1}, D_{calc} = 1.735 g·cm^{-3}, 85925 reflections measured (5.10° ≤ 2θ ≤ 56.42°). The final R1 was 0.0565 (I > 2σ(I)) and wR2 was 0.1736 (all data). CCDC no. 2459254.

^{1}H NMR (500 MHz, THF-*d8*, ppm): δ 7.49 (br, 4H), 6.57 (m, 4H), 6.36 (br, 8H), 3.55 (br 16H).

^{13}C{^{1}H} NMR (126 MHz, THF-*d8*): δ 151.51, 144.40, 118.80, 114.17, 111.82, 109.40, 34.35.

EA calc'd for $C_{56}H_{58}Bi_4N_{12}K_2O_2$·3($C_4H_8O$)[%]: C, 38.64; H, 3.75; N, 8.45; **found [%]**: C, 38.14; H, 3.76; N, 8.04. Variable amounts of THF molecules remain adhered to the two potassium ions in this compound.

IR (cm^{-1}): 615 (vw), 737 (vs), 782 (w), 816 (w), 833 (w), 909 (w), 933 (w), 1008 (w), 1034 (s), 1055 (s), 1115 (w), 1158 (m), 1183 (m), 1215 (vw), 1235 (vw), 1279 (vs), 1304 (vw), 1338 (w), 1416 (w), 1440 (m), 1480 (vs), 1506 (m), 1567 (m), 1578 (m), 1600 (w), 2781 (w), 2857 (m), 2961 (w), 3045 (vw).

Neither ESI-MS nor APCI-MS methods were able to detect the proposed product.

†Attempts to isolate **4** from polymeric byproduct *via* washing with pentane, hexane, benzene, toluene, THF, DCM and MeCN were unsuccessful, leaving either decomposed material or failing to extricate the byproduct. Filtration is possible with THF, however this similarly does not remove the byproduct.

*2.1.6 Synthesis of **5***

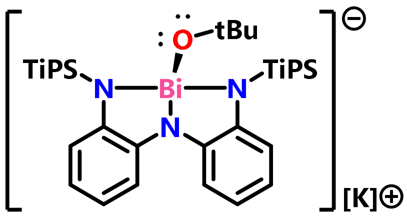

To a stirring mixture of **2b** (37.4 mg, 0.05 mmol) in THF, 5 mg (0.05 mmol) of potassium *tert*-butoxide was added. After letting react over night, the mixture was dried *in vacuo*, redissolved in benzene, filtered and dried again to give 29.1 mg (0.04 mmol) of a brown solid. Recrystallization for single crystal X-ray diffraction measurement quality crystals was done by slow vapour diffusion of pentane into THF. Yield: 69%. Crystal data for tetrameric **5** ($Bi_4N_{12}C_{136}H_{236}Si_8K_4O_4$): orthorhombic, space group $Pca2_1$ (no. 29), a = 31.604(5) Å, b = 14.541(2) Å, c = 33.340(10) Å, α = 90°, β = 90°, γ = 90°, V = 15321.5(6) Å^3, Z = 4, T = 150.00 K, μ(MoKα) = 4.083^{-1}, D_{calc} = 1.439 g·cm^{-3}, 128220 reflections measured (4.48° ≤ 2θ ≤ 52.716°). The final R1 was 0.0303 (I > 2σ(I)) and wR2 was 0.0620 (all data). CCDC no. 2459251.

^{1}H NMR (500 MHz, benzene-*d6*): δ 7.59 (dd, J = 7.8, 1.6 Hz, 2H), 7.11 (dd, J = 7.9, 1.4 Hz, 2H), 6.91 (td, J = 7.6, 1.6 Hz, 2H), 6.73 – 6.68 (m, 2H), 1.52 (h, J = 7.5 Hz, 6H), 1.26 (dd, J = 7.6, 6.1 Hz, 36H), 1.02 (s, 9H).

^{13}C{^{1}H} NMR (126 MHz, benzene-*d6*): δ 148.32, 148.12, 123.07, 119.41, 116.21, 115.75, 19.78, 15.22.

Neither ESI-MS nor APCI-MS methods were able to detect the proposed product.

2.1.7 Synthesis of ***6***

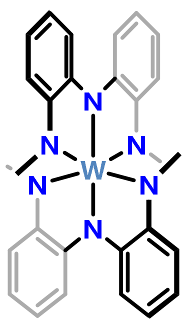

$W(CO)_5(THF)$ was first generated by irradiating a mixture of 21.1 mg (0.06 mmol) tungsten hexacarbonyl in 1 mL of THF in a quartz NMR tube with UV-C light for one hour, giving a clear, yellow solution. This was transferred into a vial containing a mixture of 52.2 mg (0.12 mmol) **2a** in 2.5 mL THF and allowed to stir overnight. No change in colour was detected, although all undissolved **2a** had disappeared as the reaction progressed to give a dark green-brown solution. This solution was filtered, leaving behind fine black solid on the filter paper, then dried *in vacuo* and washed with pentane giving 32.0 mg (0.05 mmol). Yield: 84%. Recrystallization for X-ray diffraction measurement was done by slow vapour diffusion of pentane into THF. Crystal data for dimeric **6** plus solvent ($WN_6C_{28}H_{28}$,C_4H_8O): triclinic, space group P-1 (no. 2), a = 10.7224(6) Å, b = 11.1635(6) Å, c = 12.7589(8) Å, α = 110.933(2)°, β = 101.530(2)°, γ = 92.409(2)°, V = 1386.87(14) Å^3, Z = 2, T = 150.00 K, μ(MoKα) = 4.202^{-1}, D_{calc} = 1.687 g·cm^{-3}, 47963 reflections measured (5.88° ≤ θ ≤ 50.18°). The final R1 was 0.0488 (I > 2σ(I)) and wR2 was 0.1387 (all data). CCDC no. 2459249.

^{1}H NMR (500 MHz, chloroform-*d1*, ppm): 7.76 (dt, J = 7.2, 3.6 Hz, 4H), 6.95 – 6.88 (m, 8H), 6.82 (dd, J = 6.2, 3.4 Hz, 4H), 3.32 (s, 12H).

^{13}C{^{1}H} NMR (126 MHz, chloroform-*d1*, ppm): δ 153.91, 140.89, 124.12, 119.84, 116.81, 110.62, 39.32.

^{1}H NMR (500 MHz, benzene-*d6*, ppm): δ 7.81 (dd, J = 8.2, 1.3 Hz, 4H), 6.89 (ddd, J = 8.4, 7.2, 1.4 Hz, 4H), 6.84 (ddd, J = 8.4, 7.2, 1.3 Hz, 4H), 6.55 (dd, J = 8.0, 1.4 Hz, 4H), 3.06 (s, 12H).

^{13}C{^{1}H} NMR (126 MHz, benzene-*d6*, ppm): δ 154.10, 141.08, 124.39, 120.08, 116.79, 110.84, 38.77.

IR (cm^{-1}): 480 (vw) 517 (vw), 612 (w), 726 (vs), 737 (vs), 783 (w), 817 (w), 892 (w), 1017 (s), 1033 (vs), 1052 (vs), 1112 (m), 1156 (m), 1185 (m), 1279 (vs), 1304 (s), 1344 (m), 1414 (w), 1439 (m), 1479 (vs), 1554 (m), 1574 (m), 1601 (m), 2774 (m), 2837 (m), 2846 (m), 2900 (w), 2974 (w), 3042 (vw).

APCI-HRMS (positive ion mode): calculated for $[C_{28}H_{29}N_6W]^+$ = 633.1958 m/z, **observed** = 633.1959.

m.p.: >260°C

Elemental Analysis could not be performed accurately due to the formation of tungsten nitrides and/or carbides during the combustion process.

*2.1.8 Attempted Synthesis of **2b-W(CO)$_5$***

The same procedure as for generating **6** with **2a** was used, however after 24 hours, no evidence supporting a transformation was found by NMR. Refluxing this mixture for 72 hours resulted in small amounts of decomposition to unknown products.

*2.1.9 Attempt to Detect Isobutene in Reaction Mixture of **4***

To a silanized J. Young NMR tube containing 10.0 mg (0.023 mmol) **2a** and 1.3 mg (0.012 mmol) KOtBu was added 0.75 mL of THF-*d8*, after which the tube was immediately sealed and inverted. ^{1}H NMR spectra of the sample were then gathered, showing full conversion to the byproduct mentioned above in the synthesis of **4** had already occurred and no trace of free isobutene. However, broad signals for polyisobutylene were detected and this polymer was also observed by mass spectrometry.

*2.1.10 Synthesis of [K(THF)$_2$]$_2$[**2a'**(OMe)$_2$] (**7**)*

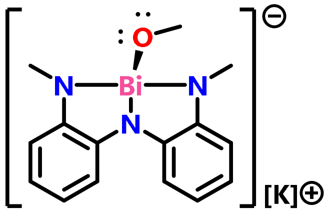

To a stirring mixture of **2a** (12.3 mg, 0.028 mmol) in THF-*d8*, 2.0 mg (0.028 mmol) of potassium methoxide was added at room temperature. After letting react for 15 minutes, the dark brown-green mixture became a clear, orange solution. Allowing the reaction to continue for one hour resulted in the mixture turning yellow and the formation of a precipitate. Filtration of the mixture resulted in decomposition of material in the solution phase to unknown products, observed by ^{1}H NMR. The precipitate was unable to be redissolved in THF. NMR data given is of the pre-filtration reaction mixture. Recrystallization for single crystal X-ray diffraction measurement quality crystals was done by slow vapour diffusion of pentane into THF. Crystal data for **7** ($BiN_3C_{22}H_{33}KO_3$): monoclinic, space group $P2_1/n$ (no. 14), a = 13.367(5) Å, b = 9.742(4) Å, c = 18.652(6) Å, α = 90°, β = 98.394(13)°, γ = 90°, V = 2402.87(15) Å^3, Z = 4, T = 150.00 K, μ(MoKα) = 7.540^{-1}, D_{calc} = 1.790 g·cm^{-3}, 54074 reflections measured (4.73° ≤ 2θ ≤ 51.70°). The final R1 was 0.0337 (I > 2σ(I)) and wR2 was 0.0922 (all data). CCDC no. 2495260.

^{1}H NMR (500 MHz, THF-*d8*, ppm): δ 7.439 (d, J = 7.6 Hz, 2H), 6.50 (t, J = 7.7 Hz, 2H), 6.16 (t, J = 7.3 Hz, 4H), 3.66 (br, 3H), 3.56 (s, 6H).

^{13}C{^{1}H} NMR (126 MHz, THF-*d8*): δ 151.51, 144.40, 118.80, 114.17, 111.82, 109.40, 34.35.

IR (cm^{-1}): 480 (vw) 517 (vw), 612 (w), 726 (vs), 737 (vs), 783 (w), 817 (w), 892 (w), 1017 (s), 1033 (vs), 1052 (vs), 1112 (m), 1156 (m), 1185 (m), 1279 (vs), 1304 (s), 1344 (m), 1414 (w), 1439 (m), 1479 (vs), 1554 (m), 1574 (m), 1601 (m), 2774 (m), 2837 (m), 2846 (m), 2900 (w), 2974 (w), 3042 (vw).

Neither ESI-MS nor APCI-MS methods were able to detect the proposed product.

*2.1.11 Synthesis of **8***

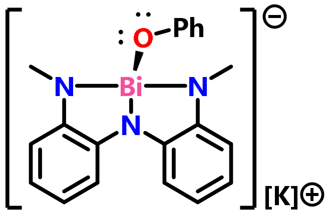

To a stirring mixture of **2a** (40.3 mg, 0.093 mmol) in THF-*d8*, 12.3 mg (0.093 mmol) of potassium phenoxide was added at room temperature. The dark brown-green mixture immediately became a clear, orange solution. Drying the product *in vacuo* resulted in its decomposition. It is, however, stable in solution for multiple days when kept at -30°C in a sealed J. Young NMR tube. We were unable to acquire single crystals suitable for X-ray diffraction.

^{1}H NMR (500 MHz, THF-*d8*, ppm): δ 7.439 (d, J = 7.6 Hz, 2H), 6.50 (t, J = 7.7 Hz, 2H), 6.16 (t, J = 7.3 Hz, 4H), 3.66 (br, 3H), 3.56 (s, 6H).

^{13}C{^{1}H} NMR (126 MHz, THF-*d8*): δ 151.51, 144.40, 118.80, 114.17, 111.82, 109.40, 34.35.

IR (cm^{-1}): 613 (vw), 699 (w), 740 (m), 822 (w), 884 (vw), 884 (vw), 983 (vw), 1033 (m), 1050 (m), 1110 (w), 1165 (w), 1181 (vw), 1279 (m), 1310 (m), 1479 (s), 1517 (w), 1556 (vw), 1585 (m), 2774 (vw), 2854 (vw), 3048 (vw).

Neither ESI-MS nor APCI-MS methods were able to detect the proposed product.

3 Spectral Data for New Compounds

3.1 NMR Spectral Data

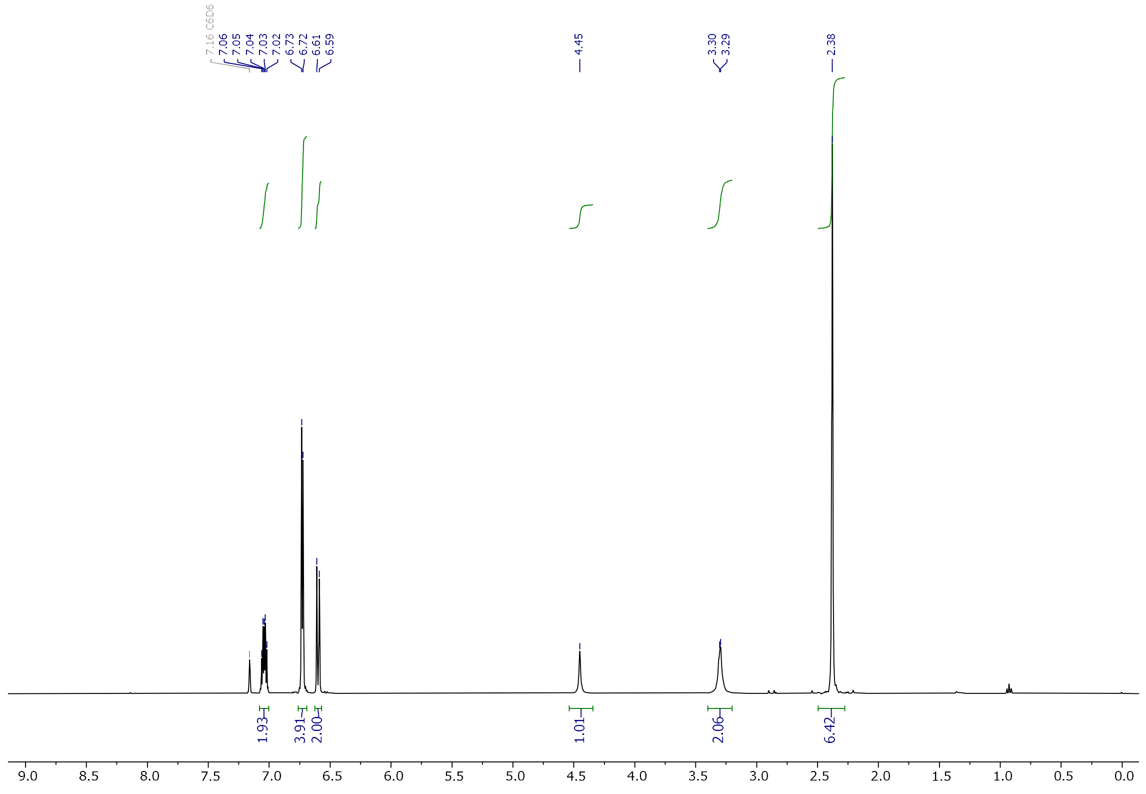

Figure S1. *^{1}H NMR spectrum (benzene-d6, 500 MHz) of* ***1a****. The peaks at 4.45 ppm and 3.30 are assigned to the central amine and terminal amine protons, respectively.*

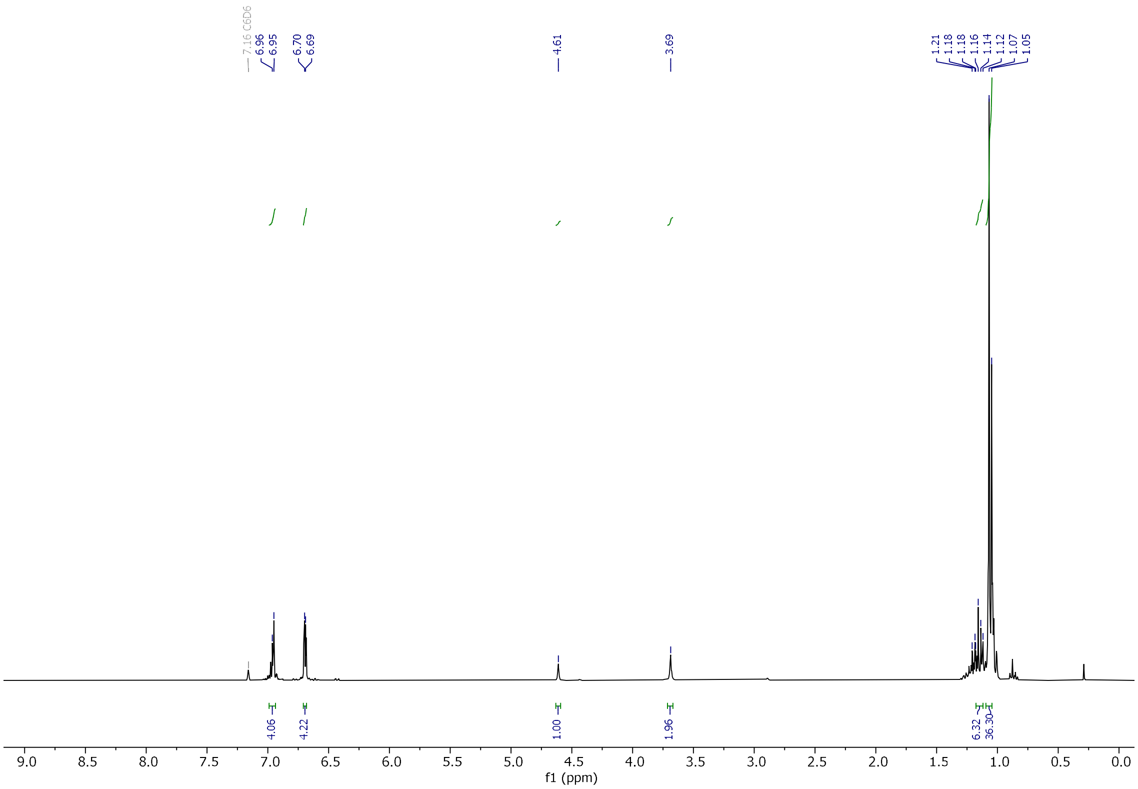

Figure S2. *^{1}H NMR spectrum (benzene-d6, 300 MHz) of* ***1b***.

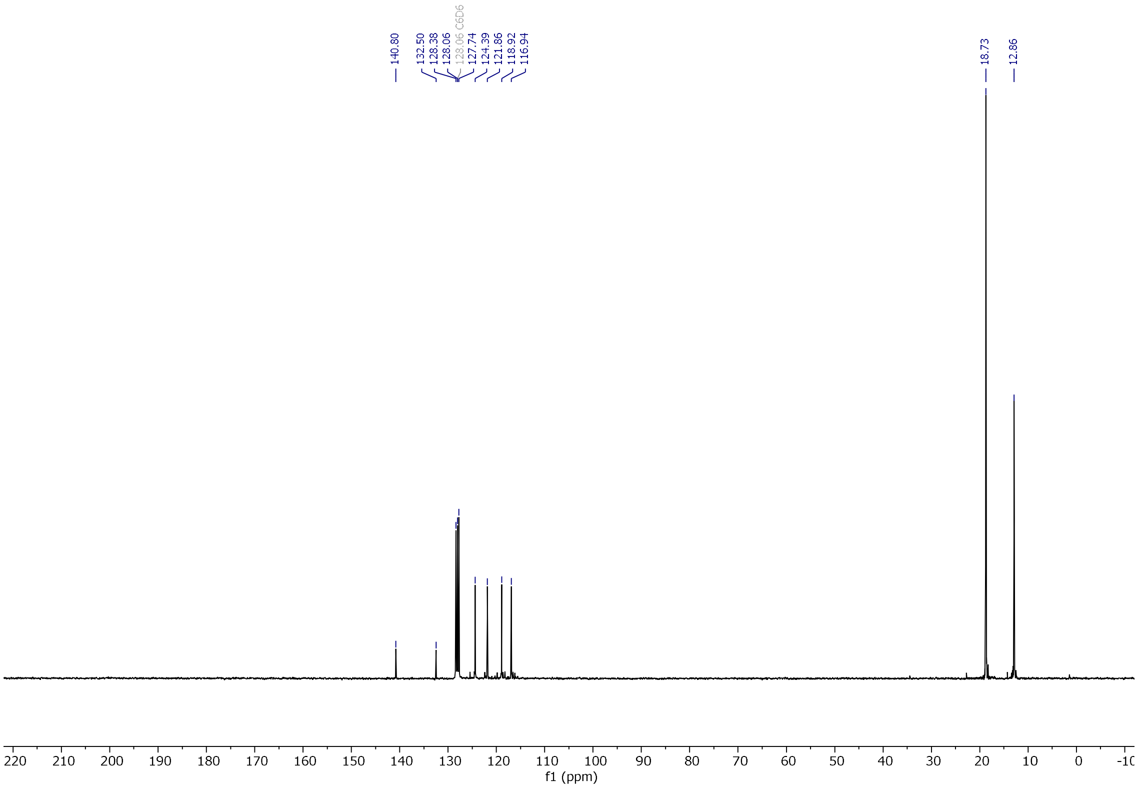

Figure S3. *^{13}C{^{1}H} NMR spectrum (benzene-d6, 70 MHz) of* ***1b***.

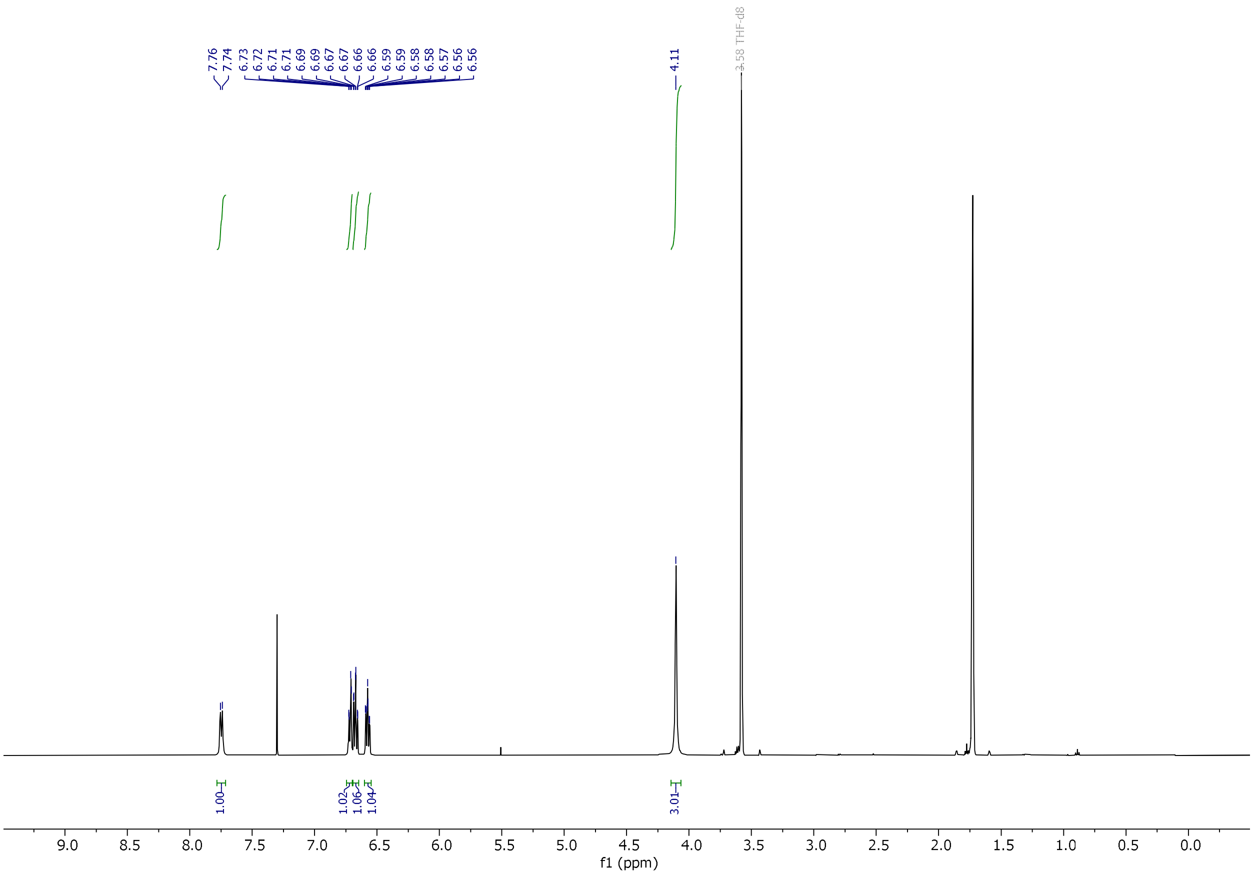

Figure S4. *^{1}H NMR spectrum (THF-d8, 500 MHz) of* ***2a***. *The peak visible at 7.30 ppm is identified as benzene impurity resulting from Na/benzophenone still used to drying of THF-d8.*

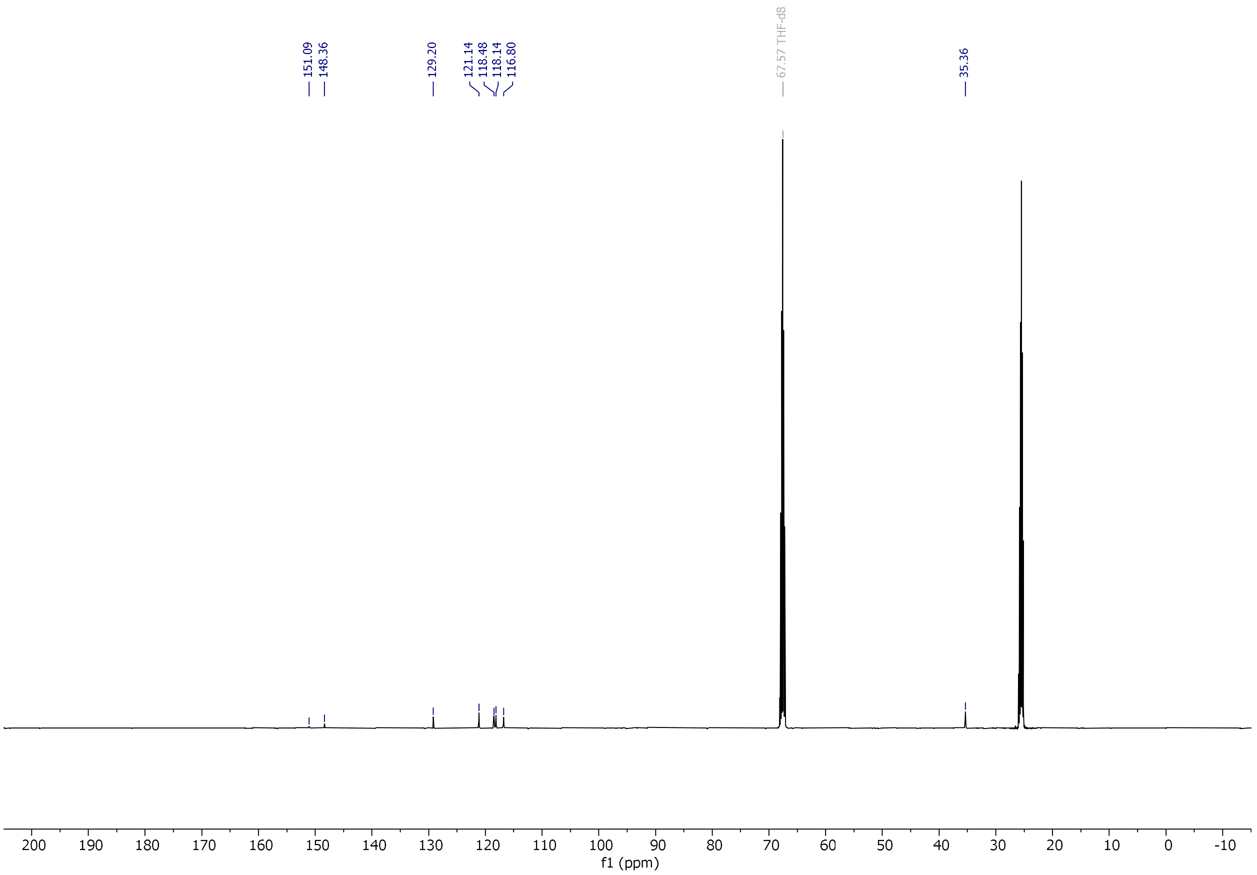

Figure S5. *$^{13}C\{^1H\}$ NMR spectrum (THF-d8, 126 MHz) of **2a**. Due to the very sparing solubility of **2a** in all solvents, <u>13 hours of scans were collected to capture this spectrum under saturated conditions</u>. The signal observed at 129.20 ppm is identified as benzene impurity.*

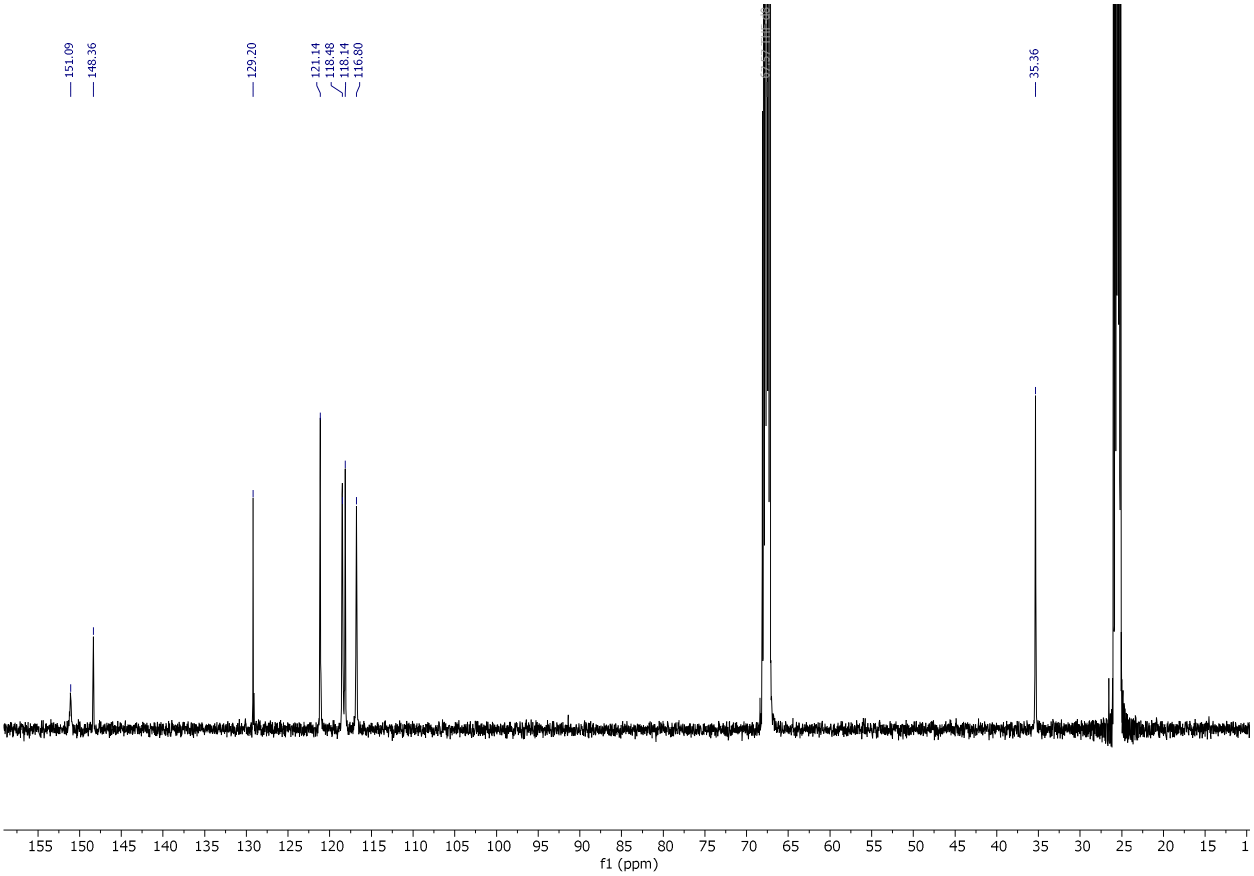

Figure S6. *Zoomed in $^{13}C\{^{1}H\}$ NMR spectrum (THF-d8, 126 MHz) of* ***2a***.

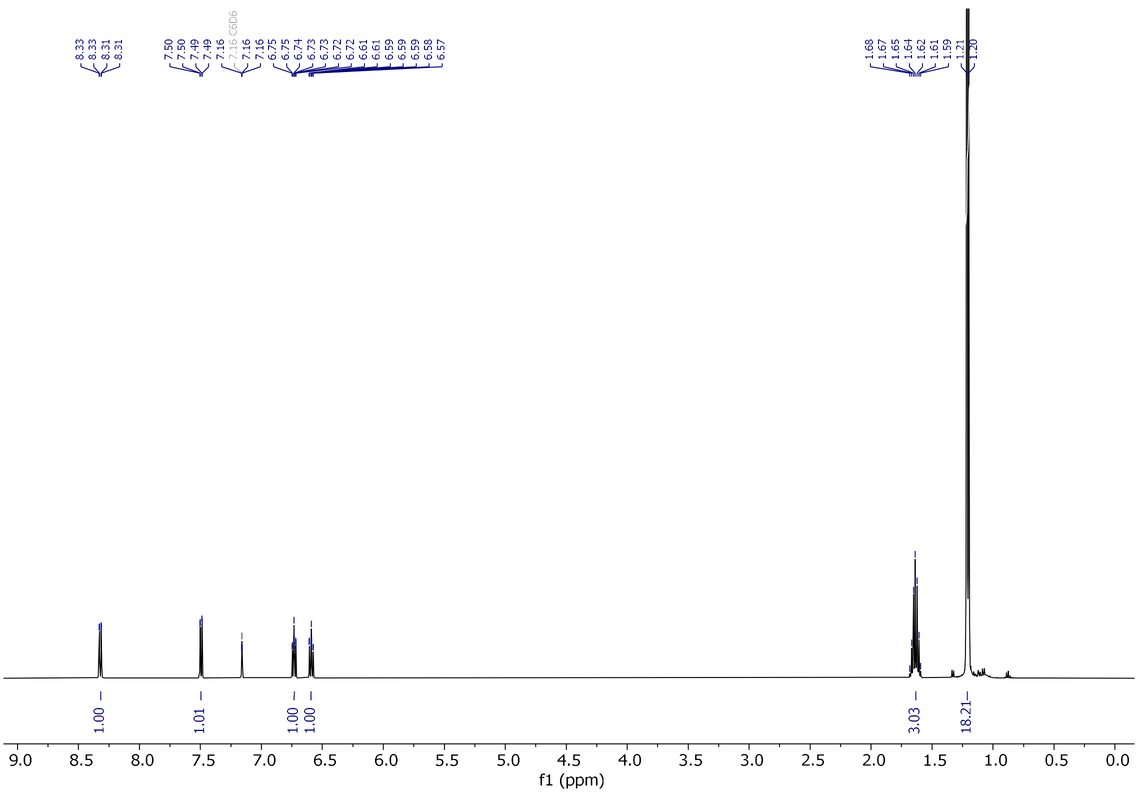

Figure S7. ^{1}H NMR spectrum (benzene-d6, 500 MHz) of **2b**.

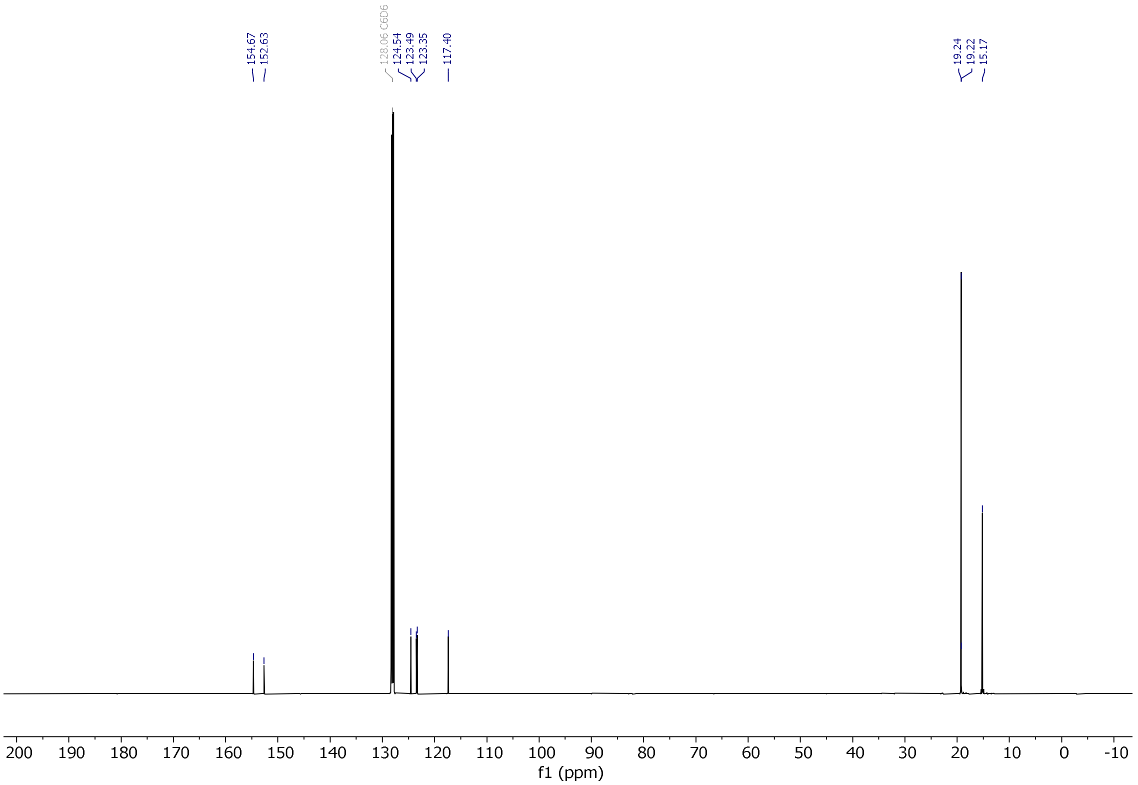

Figure S8. ^{13}C{^{1}H} NMR spectrum (benzene-d6, 126 MHz) of **2b**.

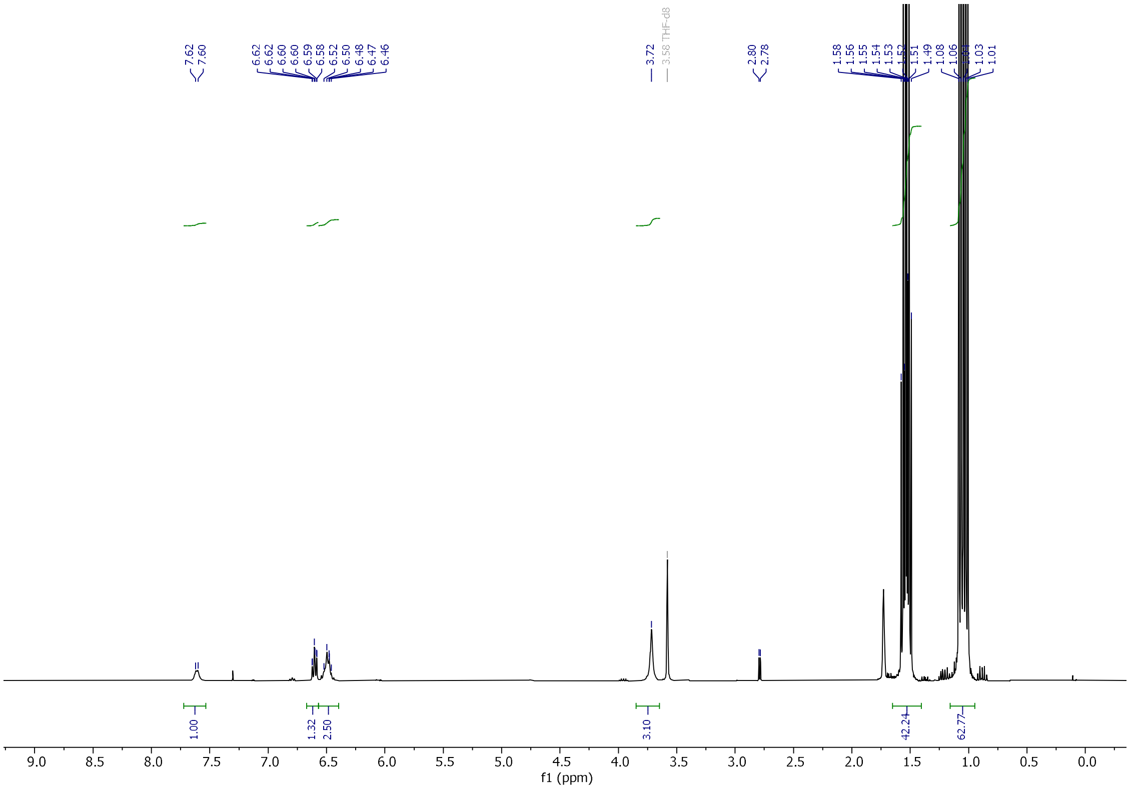

Figure S9. ^{1}*H NMR spectrum (THF-d8, 400 MHz) of* ***3***.

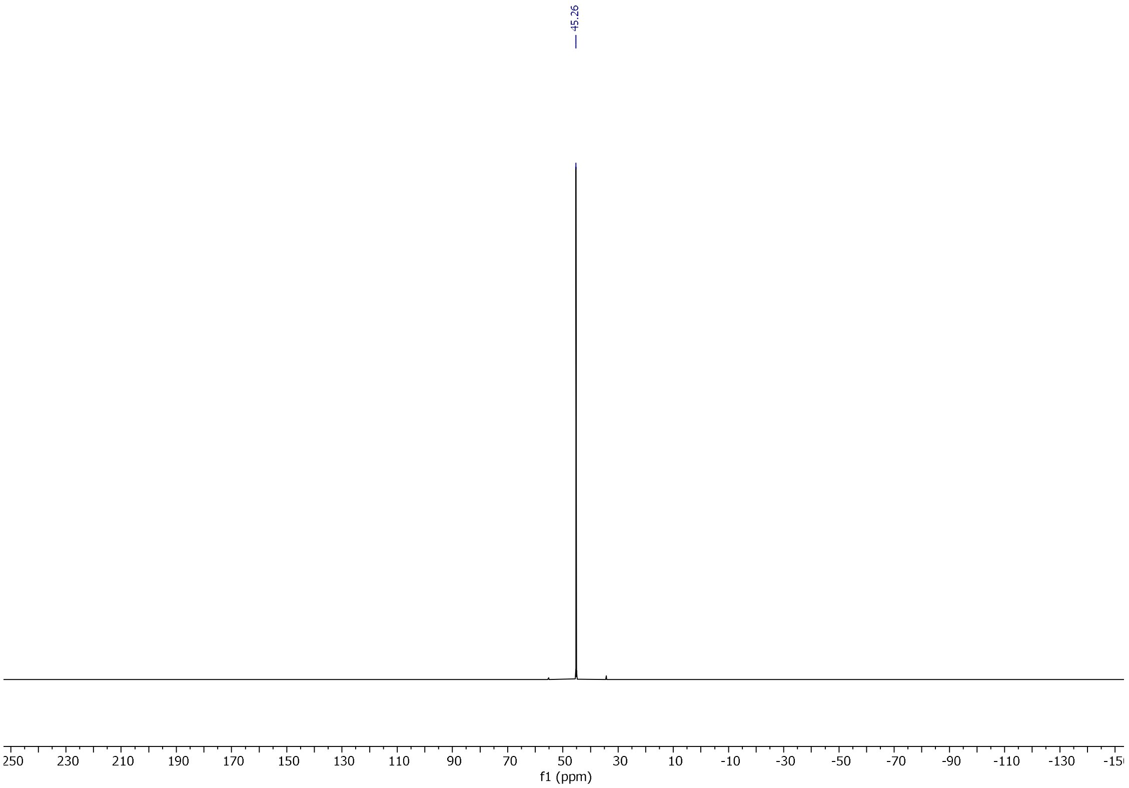

Figure S10. ^{31}*P NMR spectrum (THF-d8, 162 MHz) of* ***3***.

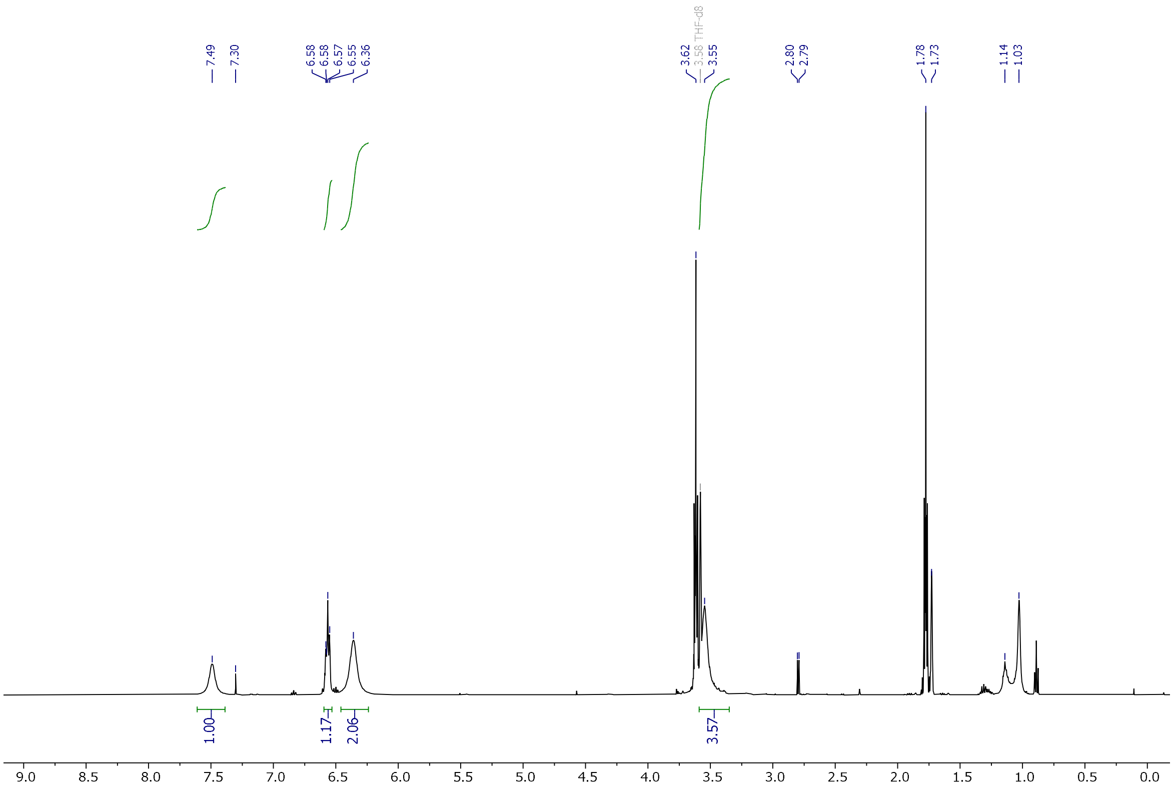

Figure S11. ^{1}H *NMR spectrum (THF-d8, 500 MHz) of **4**. The doublet at 2.80/2.79 ppm is from the NMe signal on the protio ligand. The signals at 3.58/1.73 ppm are from residual protio THF in the deuterated solvent. The signals at 3.62/1.78 ppm are from THF coordinated to potassium ions. The broad signals at 1.14/1.03 ppm are assigned to polyisobutylene formed from isobutene after its initial evolution. This polymer cannot be removed without destroying **4**, likely because growing polymer chains remains attached at one end to discrete bismuth centres, as expected for Lewis acid-initiated polymerization.[11] As bismuth centres containing the polymer strand can easily exchange with those not containing it in **4**, separation is not possible without quenching the whole sample with acids or alcohols.*

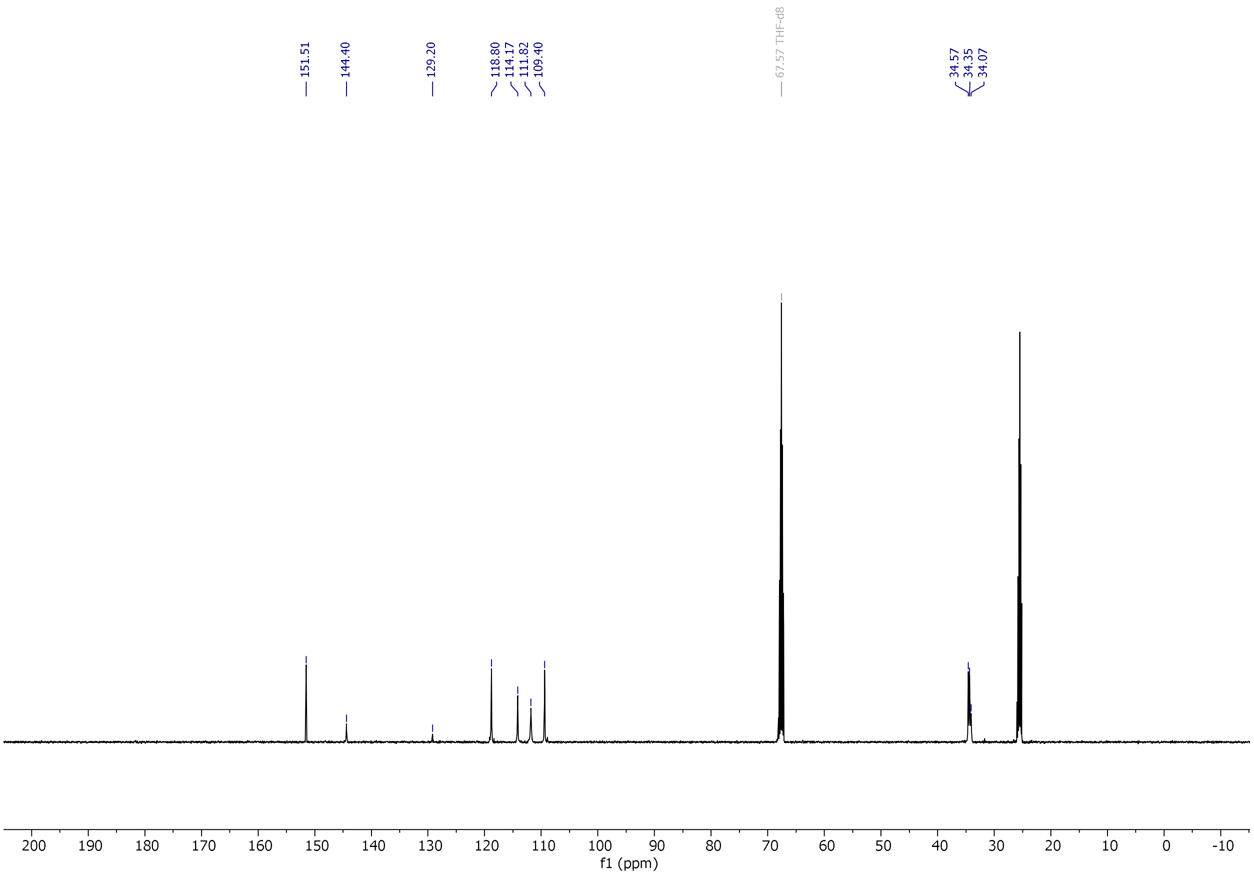

Figure S12. *$^{13}C\{^{1}H\}$ NMR spectrum (THF-d8, 126 MHz) of **4**. Signals at 34.57 ppm and 34.07 ppm corresponds to polyisobutylene, which is also detected by ^{1}H NMR and mass spectrometry. The signal at 129.20 ppm is assigned as benzene.*

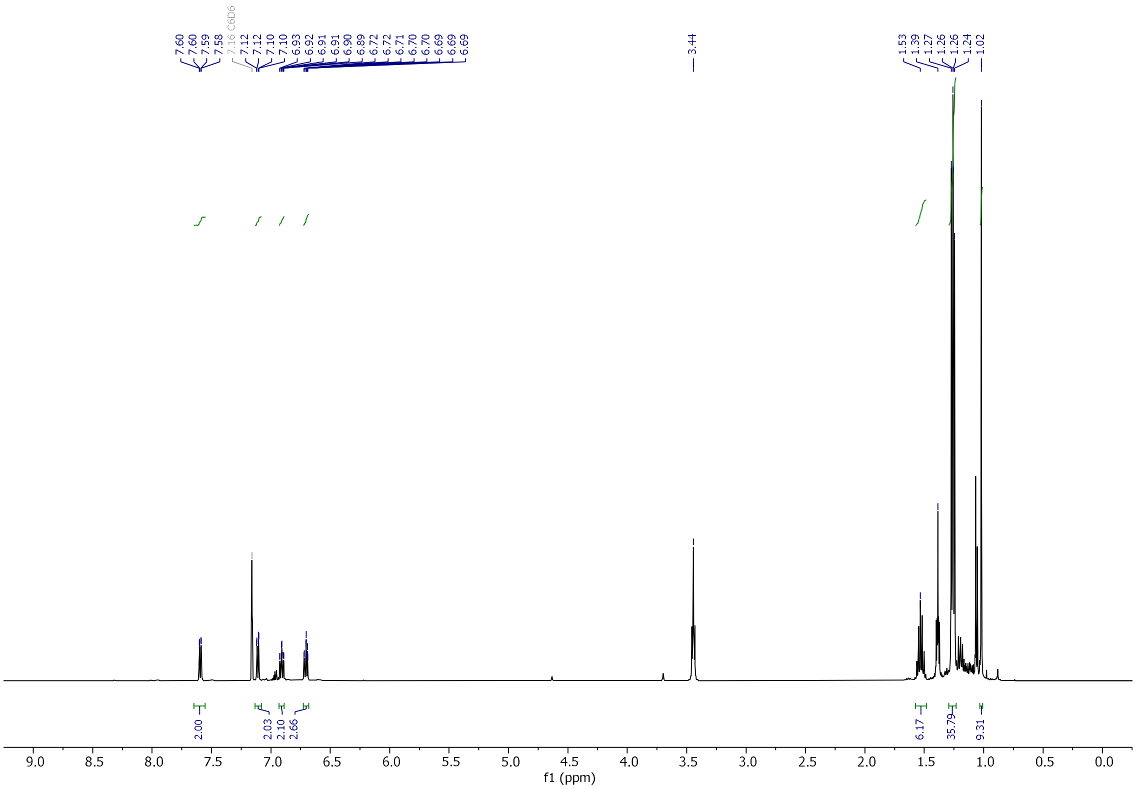

Figure S13. *^{1}H NMR spectrum (benzene-d6, 400 MHz) of* ***5***. *The signals at 3.44 ppm and 1.39 ppm correspond to residual tetrahydrofuran trapped during recrystallization.*

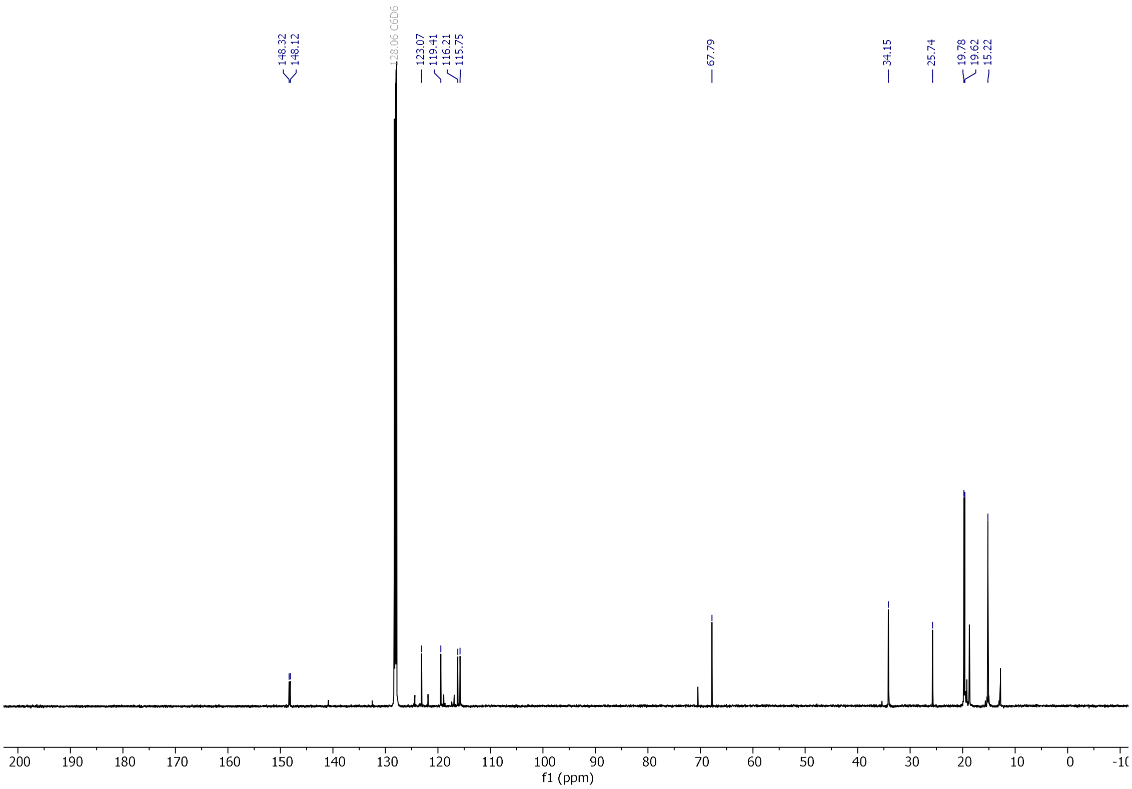

Figure S14. *$^{13}C\{^1H\}$ NMR spectrum (benzene-d6, 126 MHz) of* ***5***. *Peaks at 67.79 and 25.74 ppm are from residual THF. Peaks at 70.45 and 34.15 ppm are from unreacted KOtBu.*

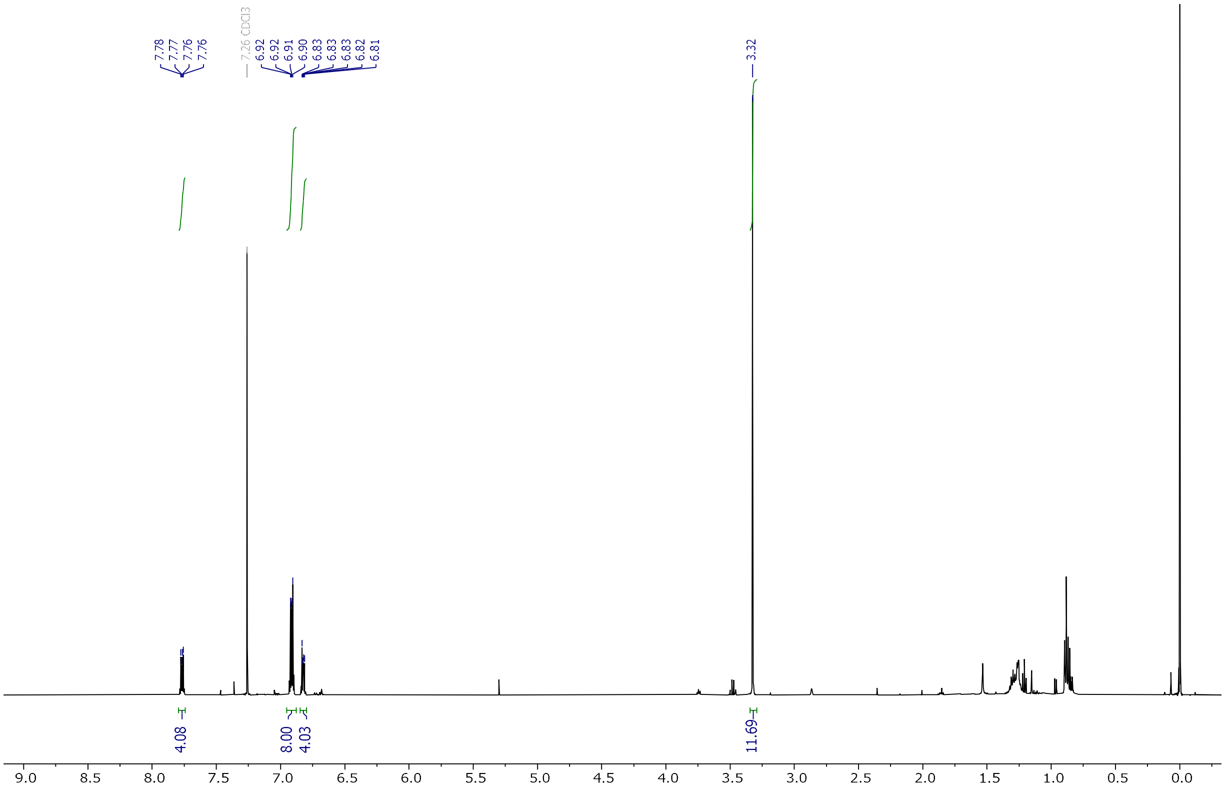

***Figure S15**. ^{1}H NMR spectrum (chloroform-d1, 500 MHz) of **6**.*

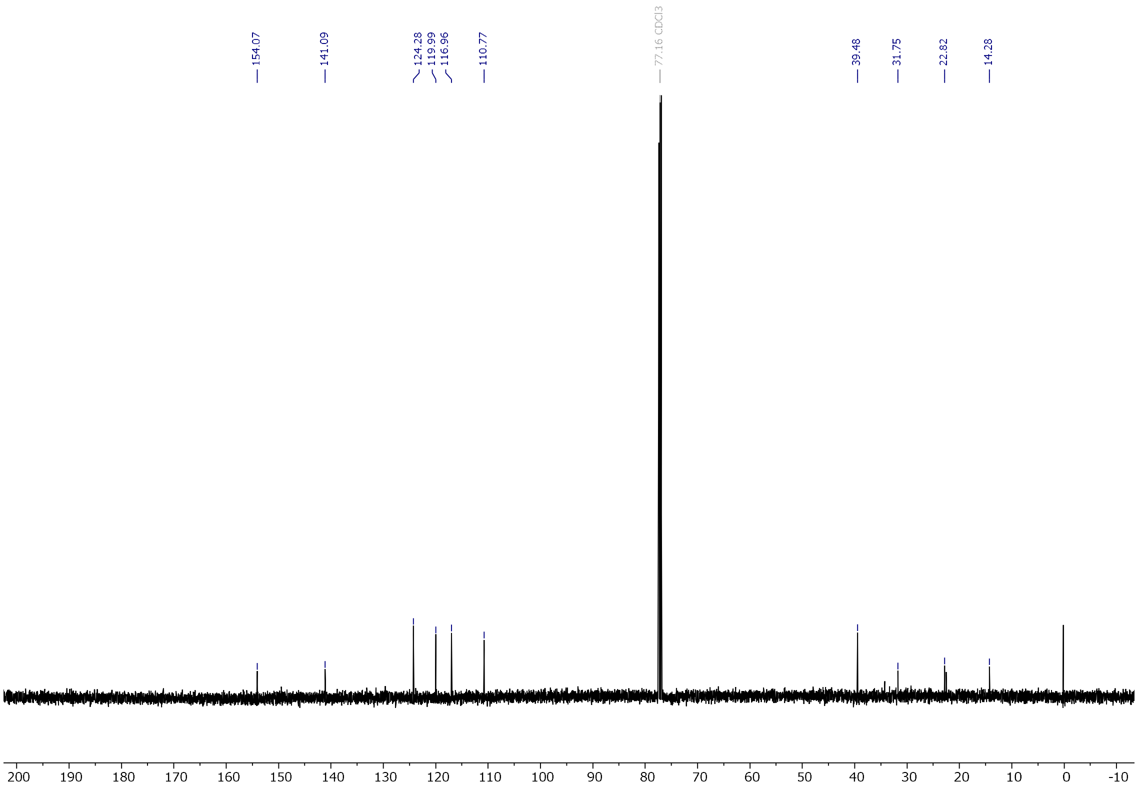

Figure S16. $^{13}C\{^{1}H\}$ *NMR spectrum (chloroform-d1, 126 MHz) of* ***6****. The peaks at 31.75 ppm, 22.2 ppm and 14.28 ppm are from residual n-hexane.*

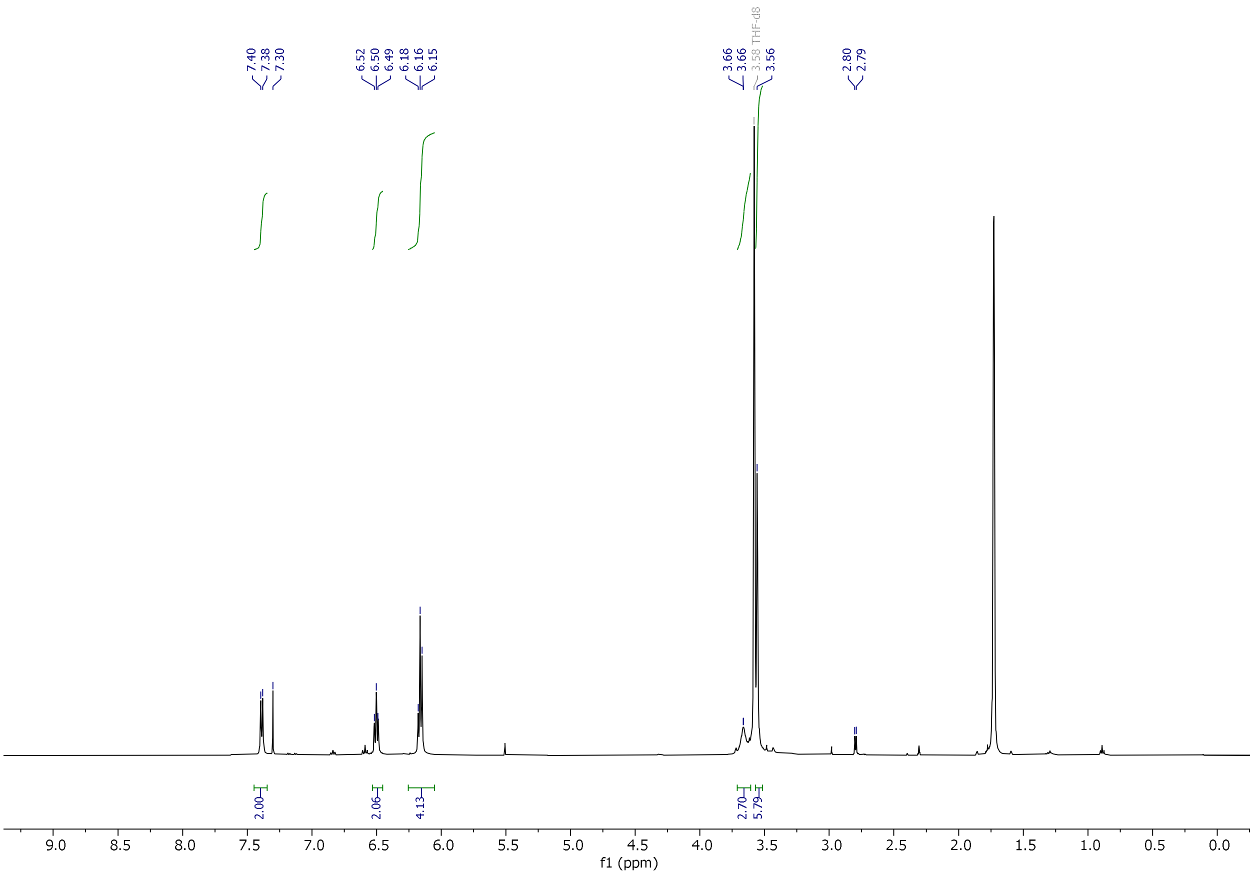

***Figure S17**. ^{1}H NMR spectrum (THF-d8, 500 MHz) of crude **7**. The peak visible at 7.30 ppm is identified as benzene impurity and the doublet signal at 2.80 ppm is the N-CH_3 proton signal from protonated ligand **1a**.*

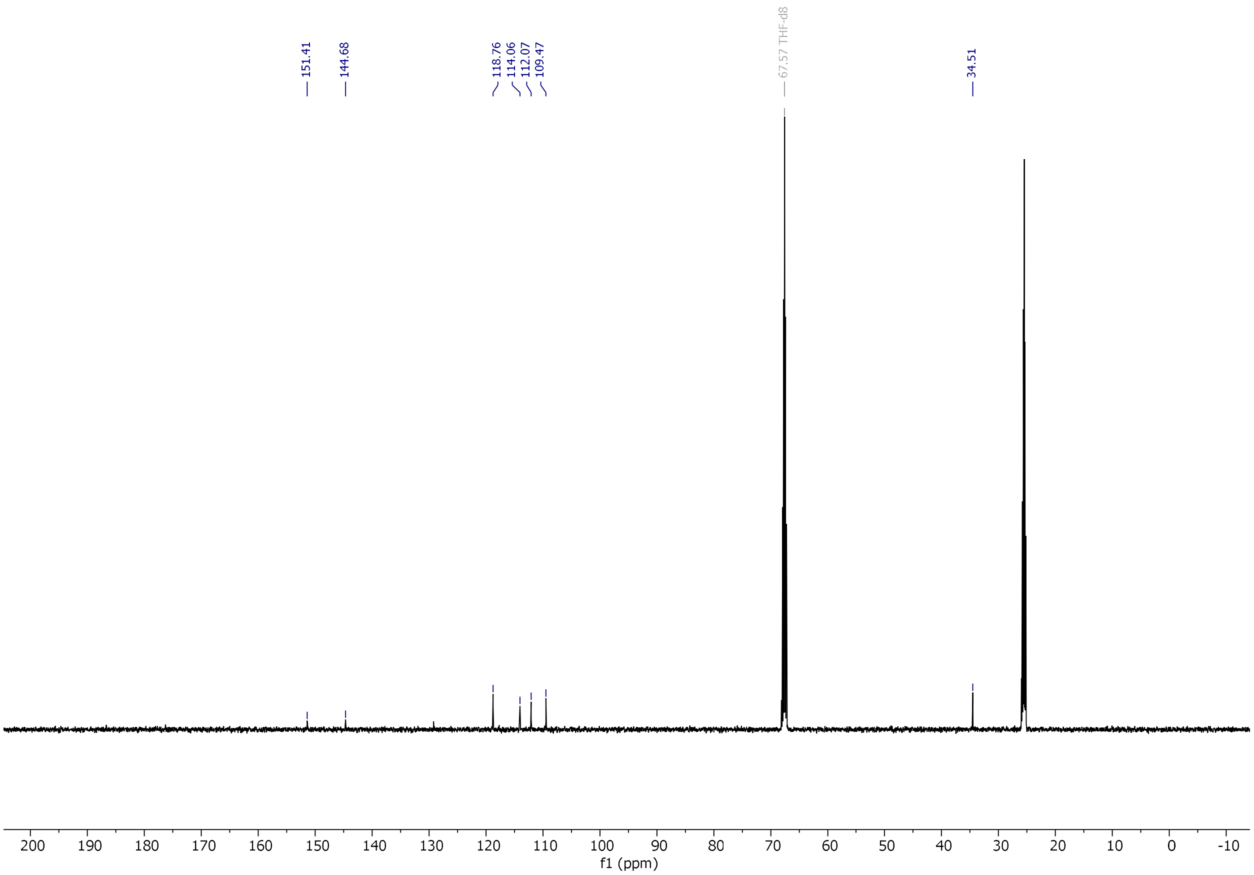

Figure S18. *$^{13}C\{^1H\}$ NMR spectrum (THF-d8, 126 MHz) of crude **7**. The methoxide peak was unable to be detected, and increasing the number of scans resulted in the detection of significant amounts of free ligand (decomposition).*

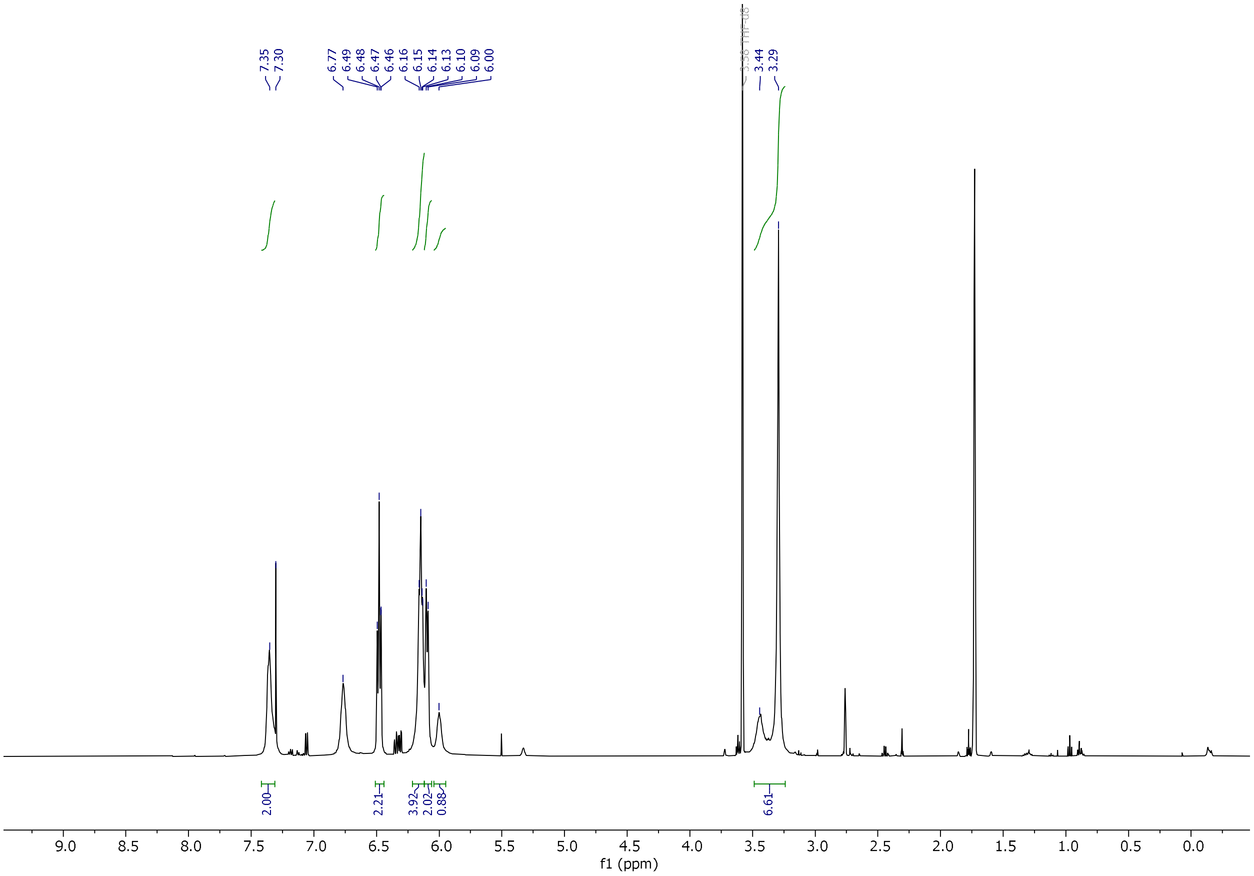

Figure S19. ^{1}H NMR spectrum (THF-d8, 500 MHz) of crude ***8***. Due to the significant broadness of these peaks assigned to ***8***, VT NMR was performed (see ***Figure S47*** and ***Section 4.2.4***).

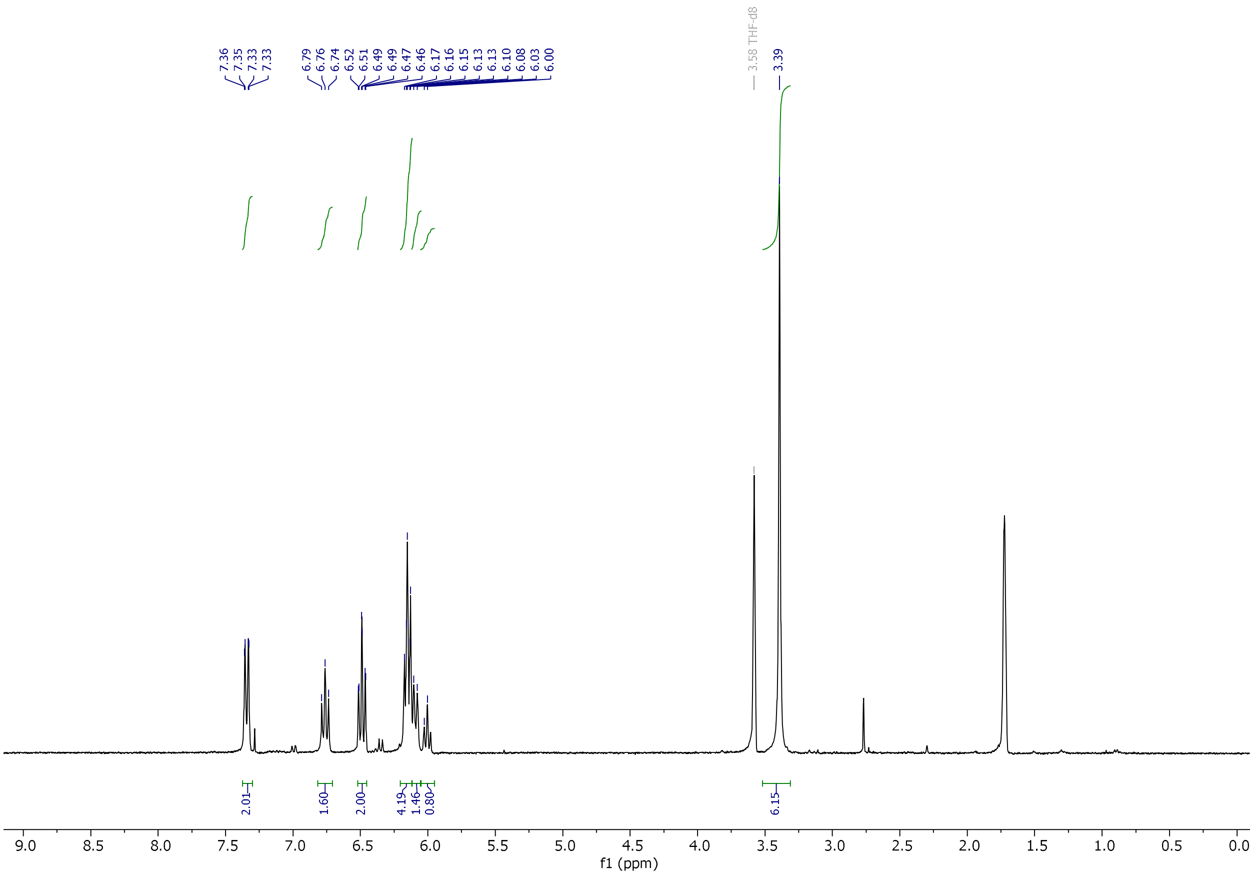

Figure S20. *^{1}H NMR spectrum (THF-d8, 500 MHz) of crude **8** at the high temperature limit (340 K).*

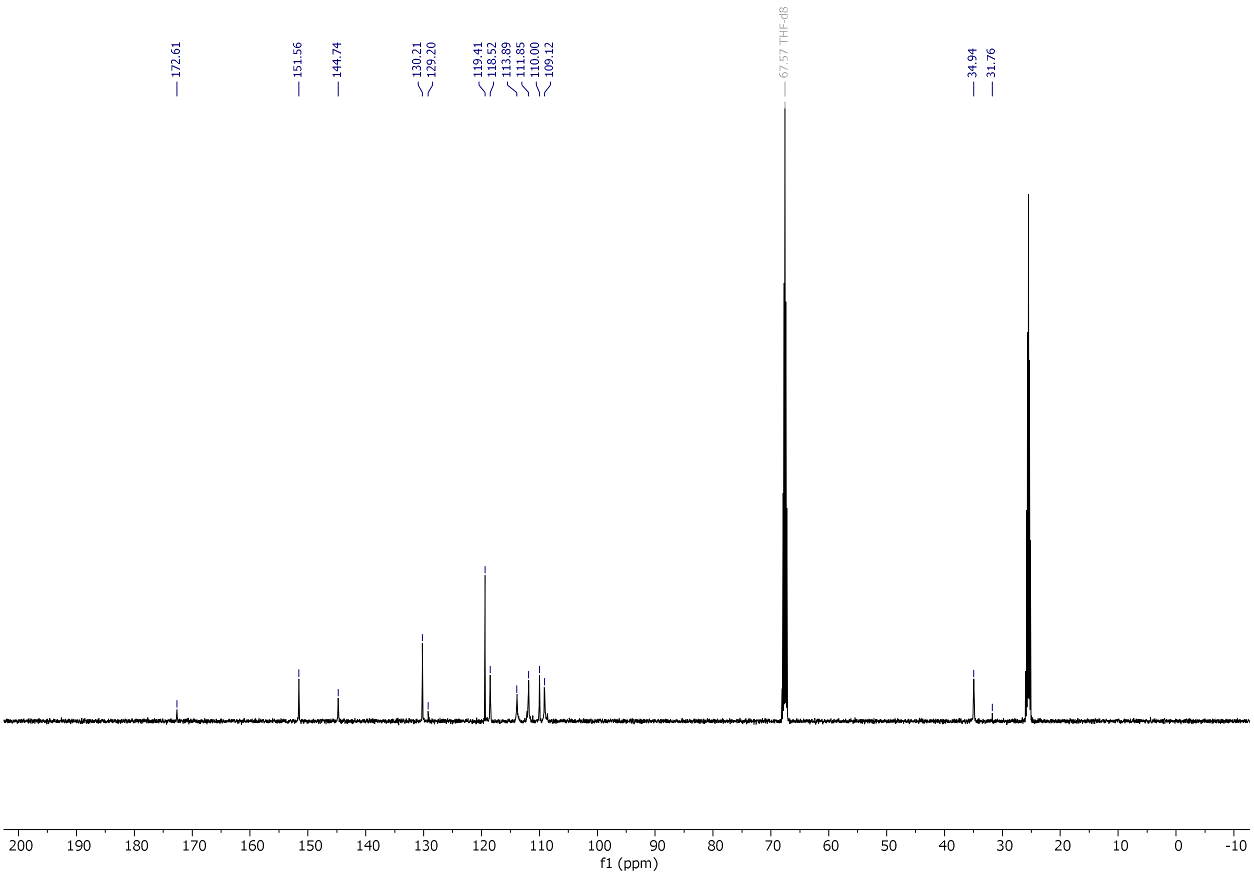

Figure S21. $^{13}C\{^{1}H\}$ *NMR spectrum (THF-d8, 126 MHz) of crude* ***8***. *Note presence of free ligand impurity at 34.94 ppm (N–CH_3) and benzene at 129.20 ppm*

3.2 IR Spectral Data

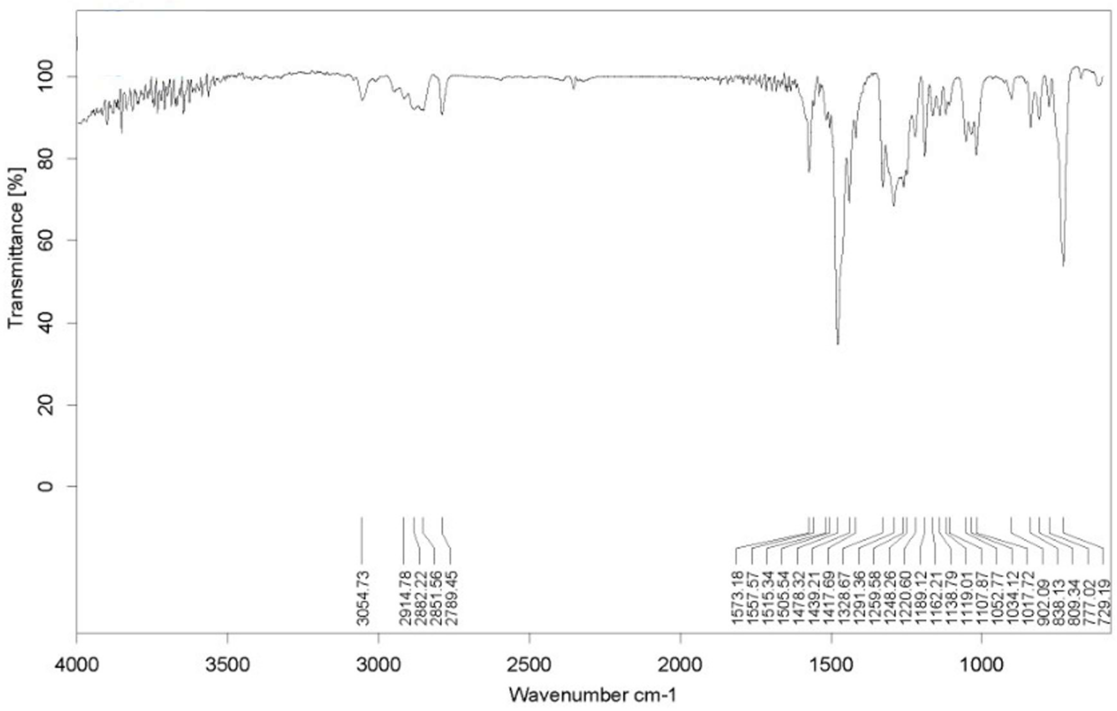

***Figure S22**. IR spectrum of compound **2a**.*

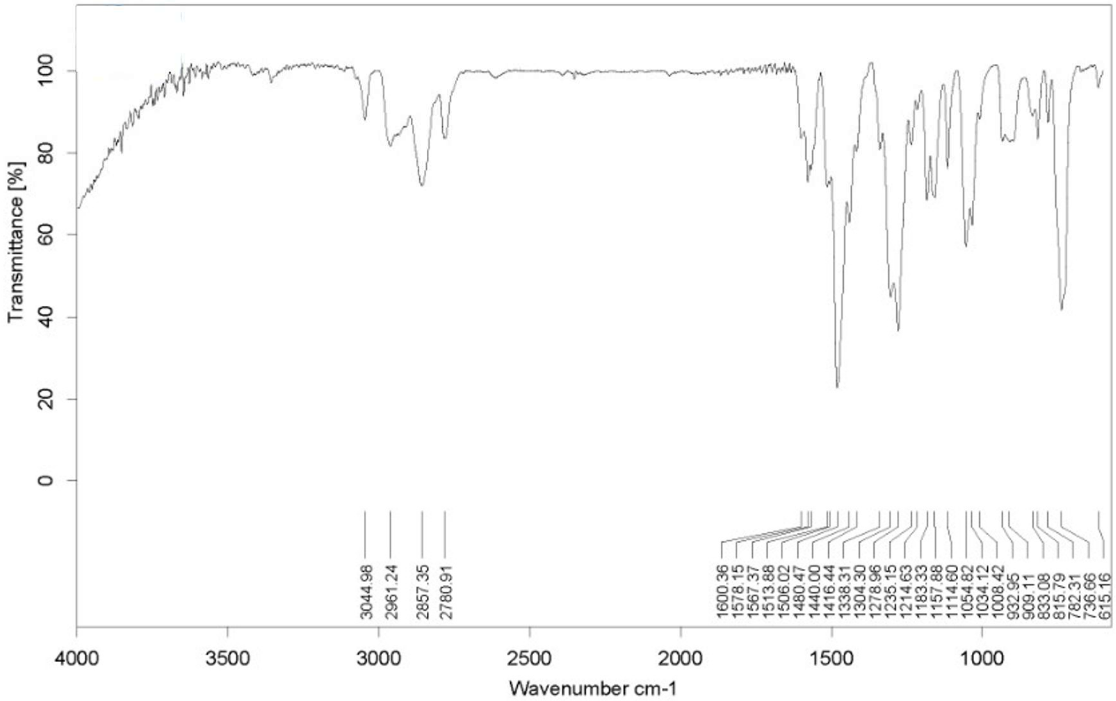

***Figure S23**. IR spectrum of compound **4**.*

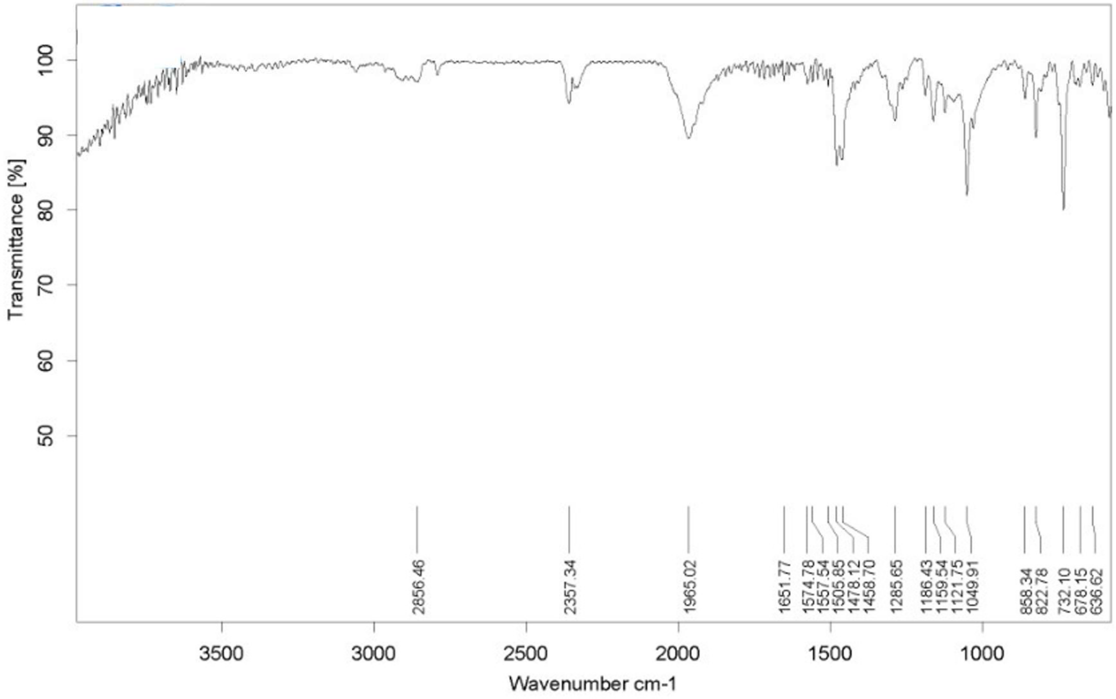

***Figure S24**. IR spectrum for compound **6**.*

`

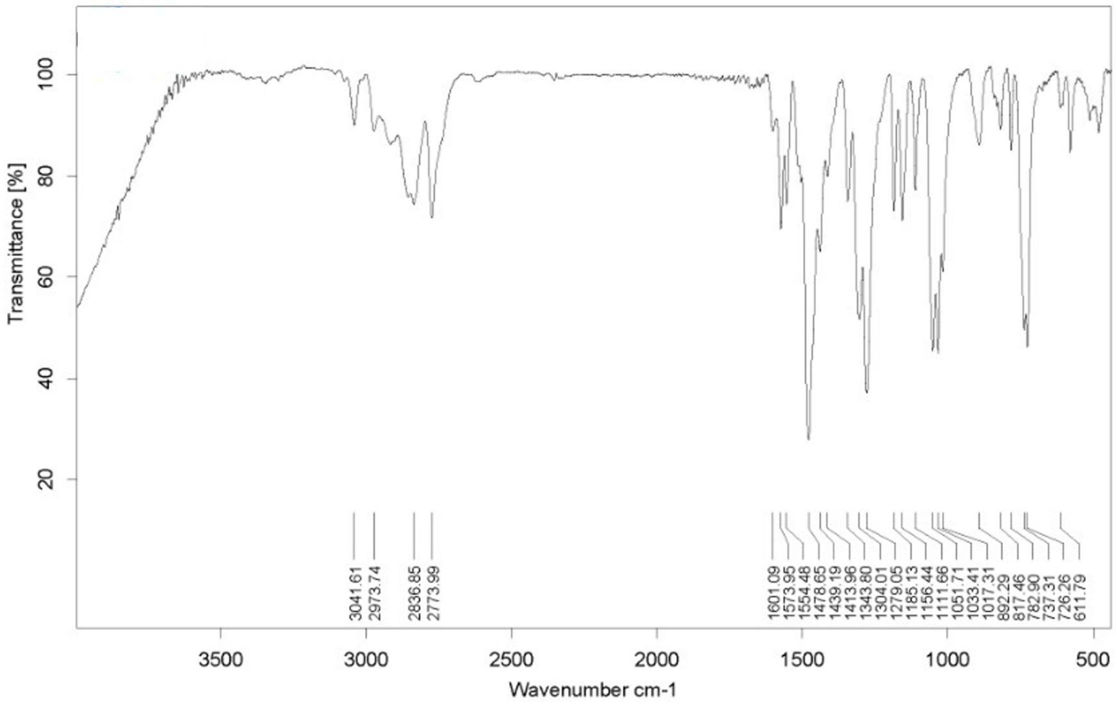

Figure S25. IR spectrum of compound **7**. The peak at 611 cm^{-1}, not present in **2a**, is indicative of a Bi–O bond.[12]

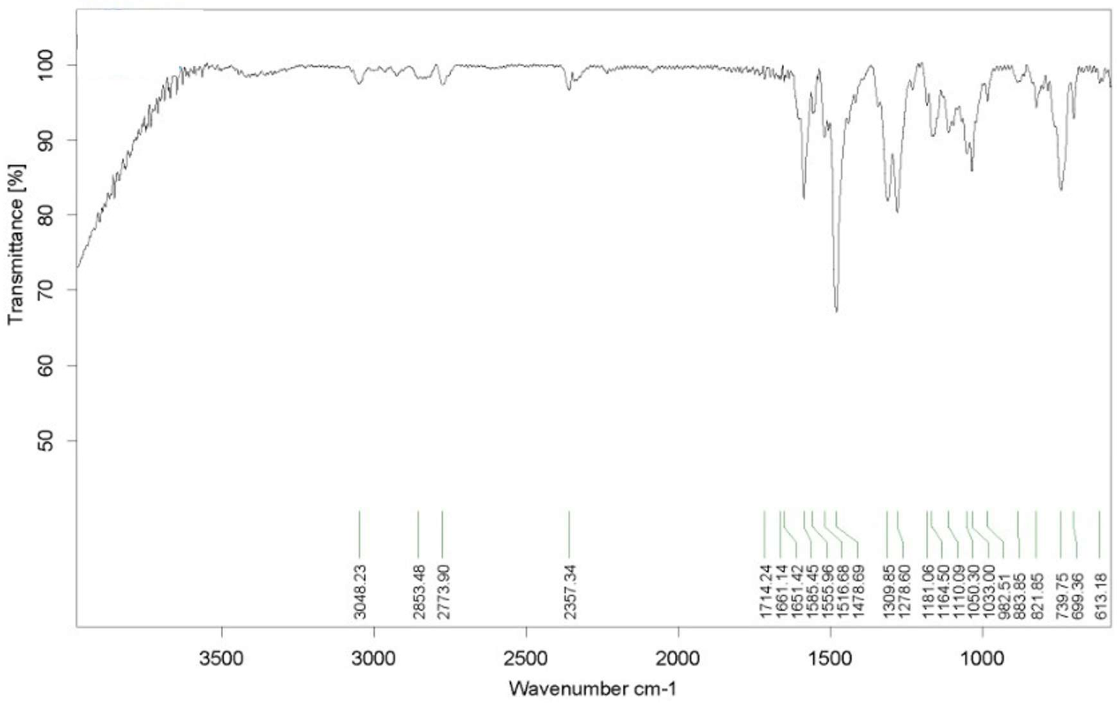

***Figure S26**. IR spectrum of compound **8**. The peak at 613 cm^{-1}, not present in **2a**, is indicative of a Bi–O bond.*[12]

3.3 Mass Spectrometry Data

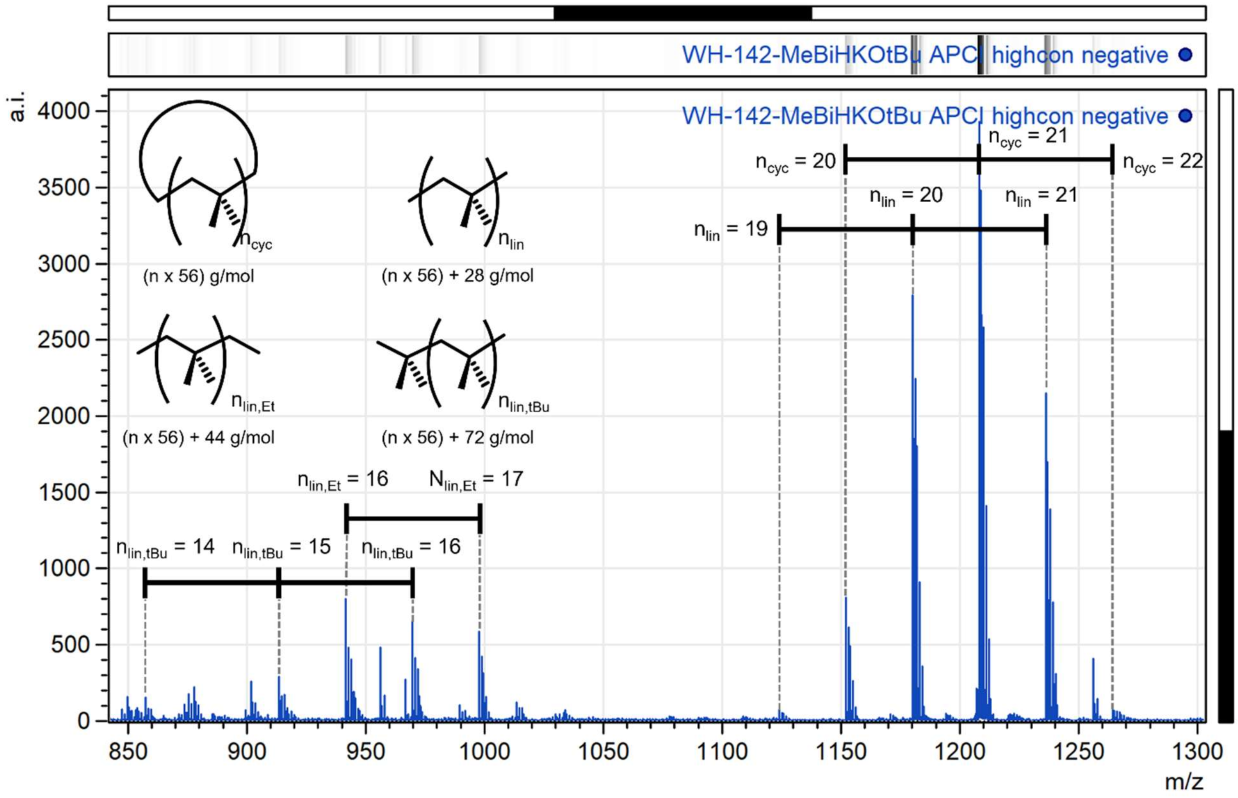

Figure S27. *850 to 1300 m/z region of mass spectrum obtained for **4**. Repeat units of polyisobutylene (monomer molecular weight = 56.06 g/mol) were detected. APCI-MS, negative ion mode.*

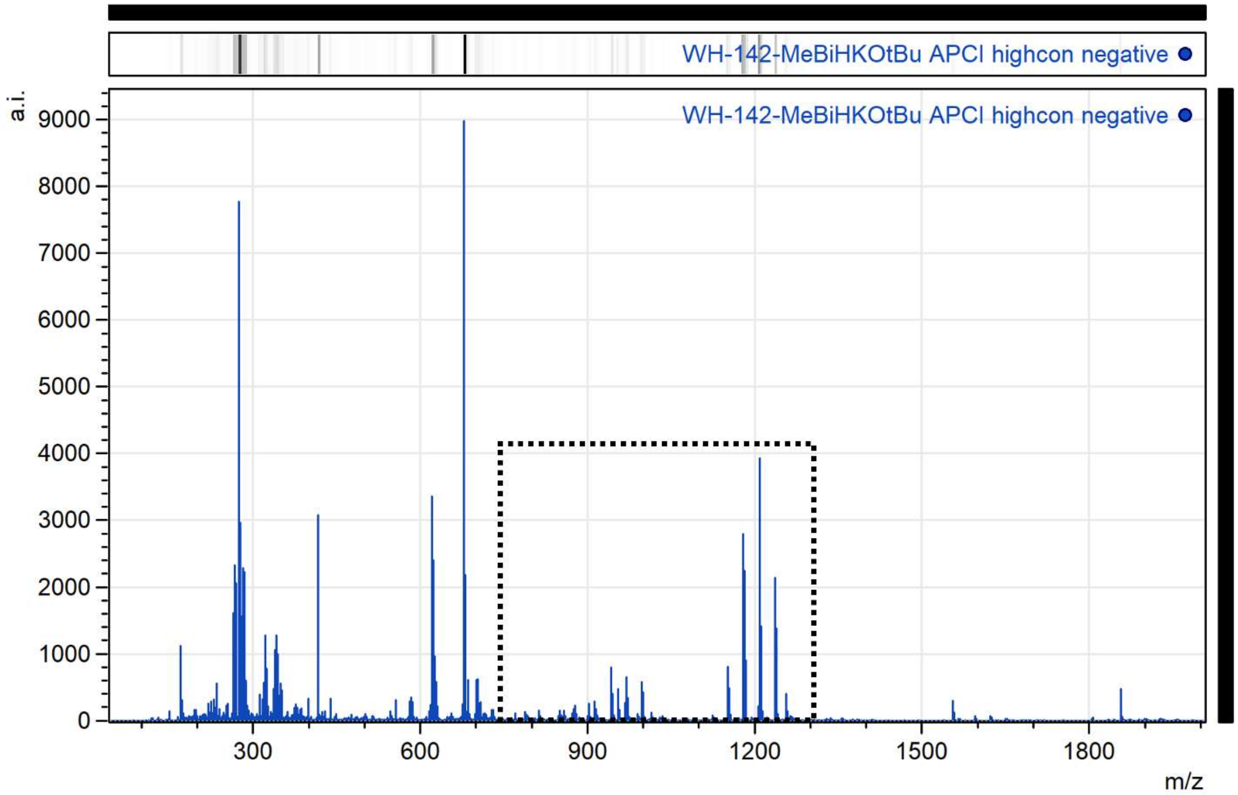

***Figure S28**. Full mass spectrum obtained for **4**. APCI-MS, negative ion mode. The target complex, **4**, was not detected by mass spectrometry, however polyisobutylene is detected as the major product (see boxed region, and expansion in **Figure S27**).*

3.4 UV-vis Spectral Data

3.4.1 Room Temperature UV-vis spectroscopy

Samples for room temperature UV-vis measurements were made by preparing 1 or 2 stock solutions. The concentration of the **2a** or **2b** in the cuvette was after measuring each UV-vis spectrum altered by addition of more solvent or stock solution to obtain the spectra at various concentrations.

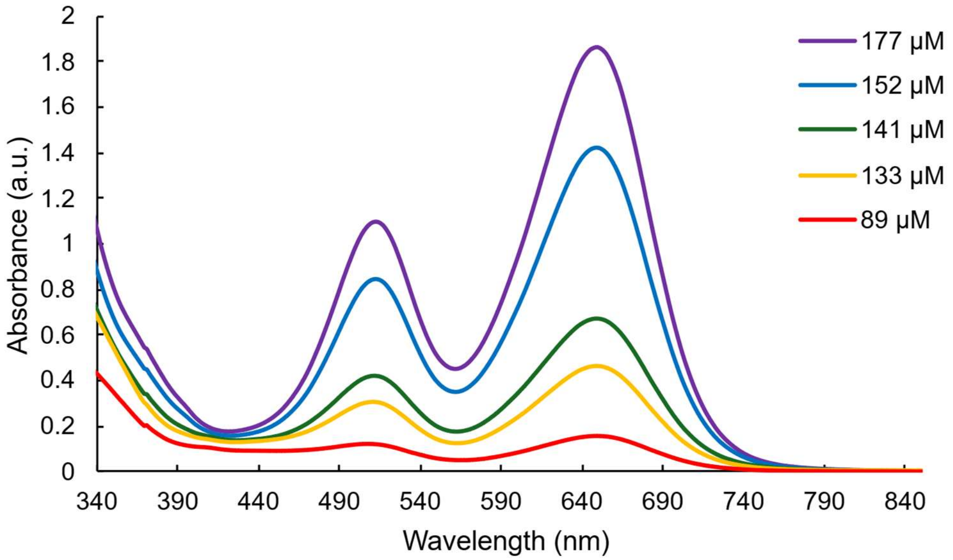

Figure S29. *Room temperature UV-vis spectra of* ***2a*** *in THF with various conditions.*

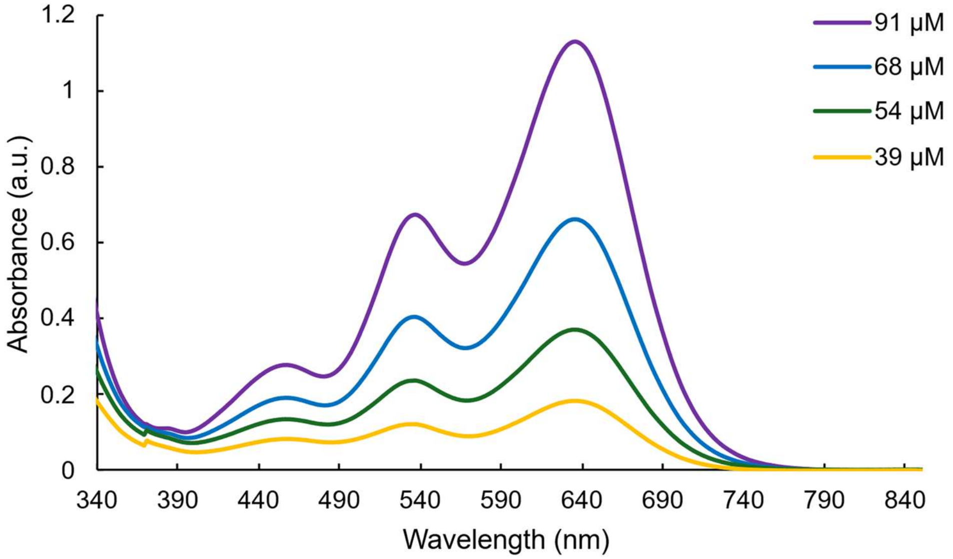

Figure S30. *Room temperature UV-vis spectra of* ***2b*** *in THF with various conditions.*

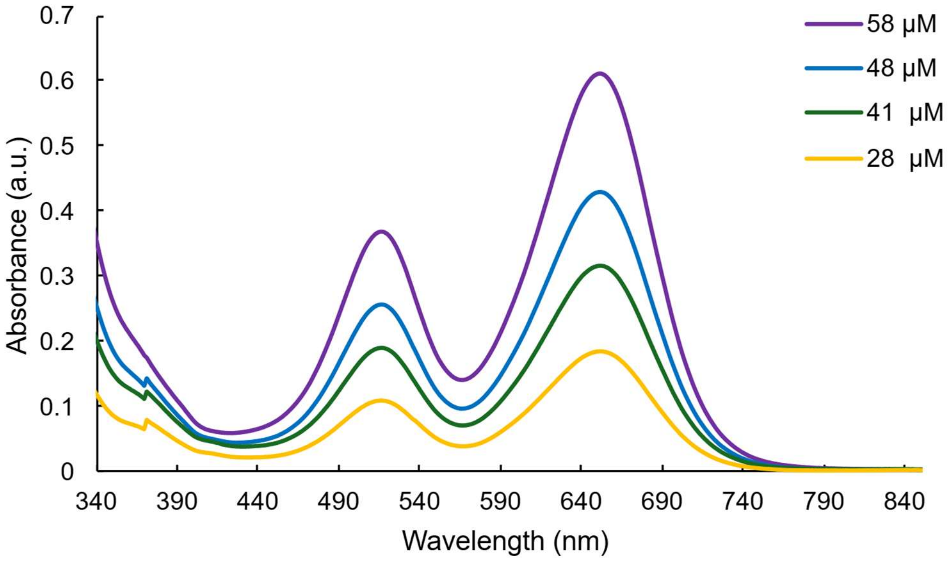

Figure S31. *Room temperature UV-vis spectra of* ***2a*** *in toluene with various conditions.*

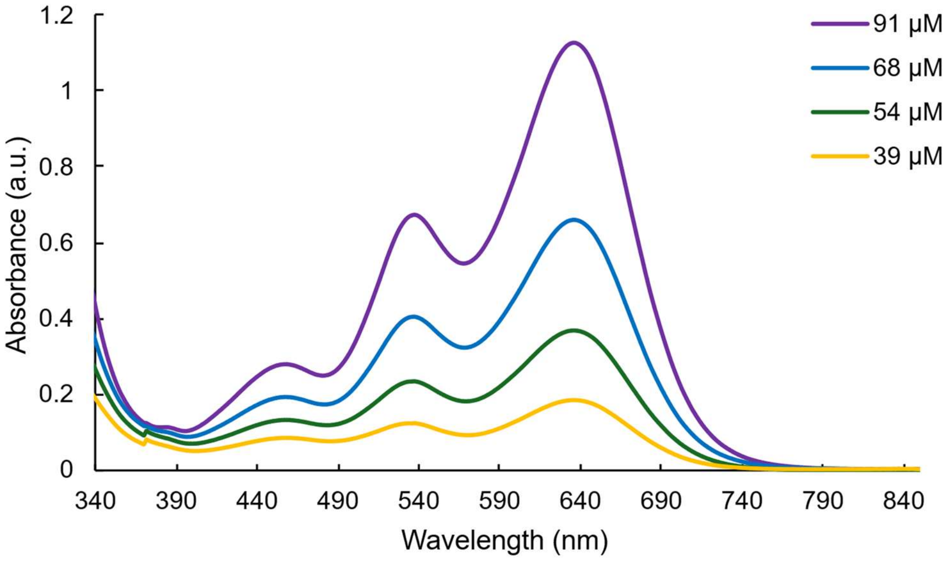

Figure S32. *Room temperature UV-vis spectra of* ***2b*** *in toluene with various conditions.*

4 Determination of Thermodynamic and Kinetic Parameters of **2a**

4.1 Variable Temperature UV-vis spectroscopy

The setup for measuring the variable temperature UV-vis spectroscopy consisted of the variable temperature cuvette holder positioned in the UV-vis spectrometer and directly connected to the thermostat (**Figure S33**). To prevent condensation and ice formation on the windows of the cuvette, a constant stream of nitrogen gas was blown at both sides of the cuvette during the whole time. For the measurements, only the tungsten lamp was used.

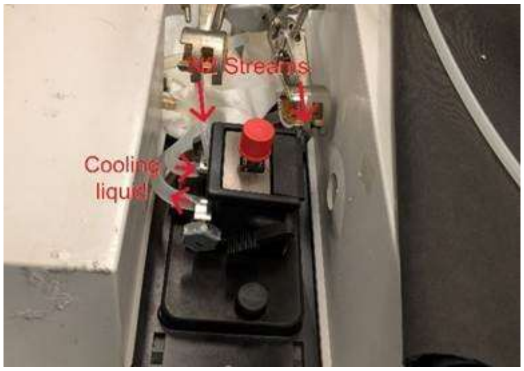

Figure S33. *Setup of the cuvette holder in the UV-vis spectrometer.*

For each measurement, the cuvette filled with 2.0 mL solution was placed in the spectrometer with the internal bath temperature of the thermostat set to 20 °C. After an equilibration time of 20 minutes, the internal bath temperature was set to decrease by 1 °C per minute, while every 5 minutes a spectrum was measured. Initial calibration showed that while the temperature was dependent on the positioning of the equipment, the temperature showed to be reproducible (maximum standard deviation of 0.36 °C over three runs up to and including 60 minutes) within the same experimental setup over three different runs while repositioning the thermal couple (calibrated using an ice bath at 0 °C) inside the cuvette (**Figure S34**). Therefore, all measurements were taken at a single day without altering the setup and determining the temperature of the solution once at the same day using a blank cuvette filled with 2.0 mL THF.

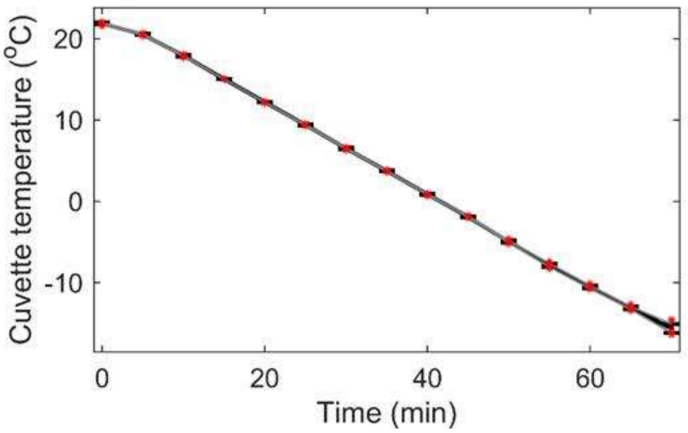

***Figure S34**. In triplo calibration of the temperature inside the cuvette filed with 2.0 mL THF over time after initial equilibration and showing the standard deviation in grey.*

The temperature dependent UV-vis spectra were measured in triplicate, with each time a new sample prepared with a concentration of 0.126 mM (**Figure S35**). After finishing the measurements, the cuvette was warmed up back to the starting conditions. The ratios at both the isosbestic point and peak maxima indicate the decomposition to be ±5% in all cases (**Figure S36**). Since it is unclear when during the measurements the decomposition occurred (e.g. is the monomer **2a** or dimer **2a'** more stable) and the amount of decomposition in all cases small, the initial concentration was used during the peak fitting process.

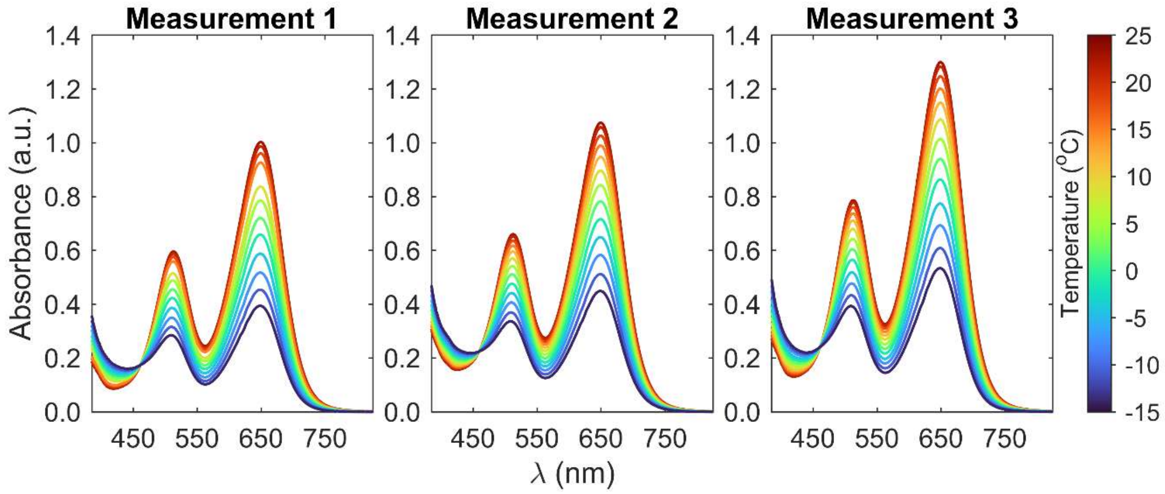

***Figure S35**. Temperature dependent UV-vis spectra of **2a** in THF (all 0.126 mM).*

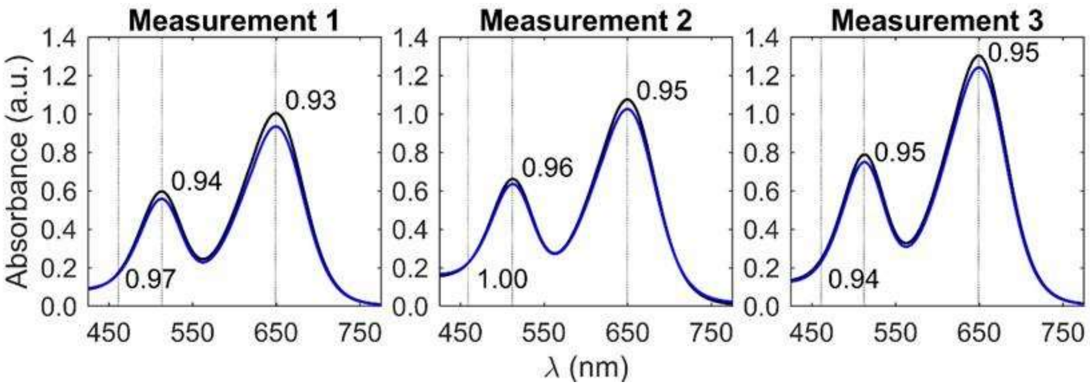

***Figure S36**. Estimation of the stability of the UV-vis sample of **2a** in THF (0.126 mM) by comparing the UV-vis spectrum before (black) and after (blue) the measurements under the starting conditions. The reported numbers indicate the ratio of absorbance at the isosbestic point and the two maxima between the two spectra.*

To obtain the thermodynamic parameters of the monomer–dimer equilibrium, the peak maxima of each measurement were fitted with $A = \epsilon(\lambda) * c_{monomer} = \epsilon(\lambda) * \frac{-1+\sqrt{1+8*K*c_0}}{4*K}$ with c_0 being the starting concentration of the monomer and the equilibrium constant $K = e^{-(\Delta H - T\Delta S)/(RT)}$ using Origin 2018.[13] For the fitting, absorption at the peak maxima at 512 (513 for measurement 1) and 649 nm were taken. For the data shown in **Table S1**, results of the fitting curves of the peak maxima shown in **Figure S37**, the error for each measurement is the fitting error given by Origin. For the average and total columns, the error is the standard deviation of the three and six independent measurements, respectively. Since the wavelength of maximum absorption at 512 nm shifted slightly to longer wavelengths due to interference of the absorption of the dimer **2a'**, the results at 649 nm are more reliable and therefore used in the manuscript.

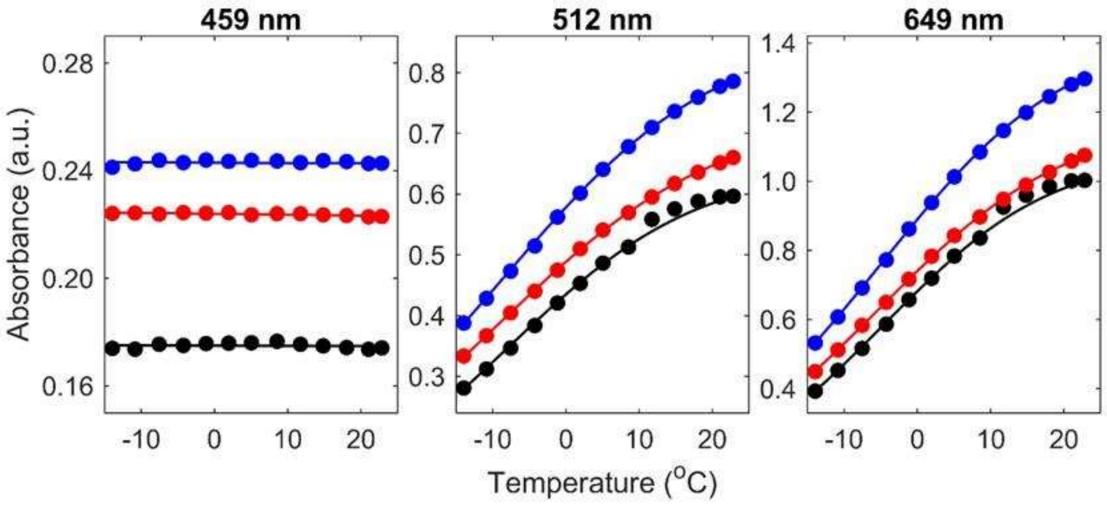

***Figure S37**. Fitting of the isosbestic point and absorption maxima in the temperature dependent UV-vis spectra of **Figure S35**. Measurement 1: black; Measurement 2: red; Measurement 3: blue.*

***Table S1**. Results of the fitting curves of the peak maxima shown in **Figure S37**.*

**Parameter**	**Measurement 1**		**Measurement 2**		**Measurement 3**		**Average**		**Total**
	513 nm	649 nm	512 nm	649 nm	512 nm	649 nm	512 nm	649 nm	
**ε [M^{-1} cm^{-1}]**	5139 ± 34	8589 ± 13	5815 ± 26	9371 ± 47	6914 ± 41	11332 ± 40	5956 ± 896	9764 ± 1413	
**ΔH [kJ mol^{-1}]**	–60.51 ± 0.58	–69.43 ± 0.41	–53.48 ± 0.35	–63.08 ± 0.37	–55.05 ± 0.46	–64.75 ± 0.26	–56.35 ± 3.69	–65.75 ± 3.29	–61.1 ± 6.0
**ΔS [J mol^{-1} K^{-1}]**	–155.3 ± 2.3	–185.9 ± 1.7	–129.3 ± 1.4	–162.5 ± 1.5	–134.9 ± 1.9	–168.4 ± 1.1	–139.8 ± 13.7	–172.3 ± 12.2	–156.1 ± 21.2
**$\Delta G_{293\,K}$ [kJ mol^{-1}]**	–15.01	–14.96	–15.60	–15.47	–15.52	–15.41	–15.38 ± 0.32	–15.28 ± 0.28	–15.33 ± 0.27
**$\Delta G_{298\,K}$ [kJ mol^{-1}]**	–14.23	–14.03	–14.95	–14.66	–14.85	–14.57	–14.68 ± 0.39	–14.42 ± 0.34	–14.55 ± 0.36

4.1.1 Variable Temperature UV-vis spectroscopy in Toluene

For the temperature dependent UV-vis measurements of **2a** in toluene, samples close to the maximum solubility of **2a** in toluene. Since this concentration is very small (<1 mg per

20 mL of toluene), accurate concentrations are difficult to obtain. Furthermore, for two out of three measurements significant decomposition of the complex was observed (15 % and 25 %), indicating the potential of the concentration decreasing over time. Together, these two points of concern resulted in significantly larger discrepancies between the three measurements compared to the measurements in THF. The corresponding larger error margins make it impossible to observe any distinct differences between the measurements in toluene and THF.

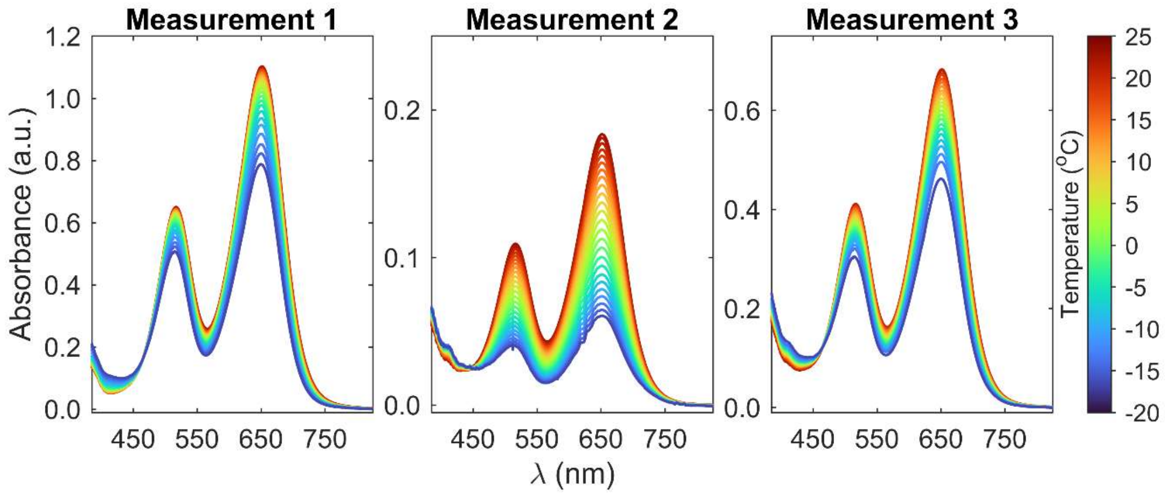

Figure S38. *Temperature dependent UV-vis spectra of* ***2a*** *in Toluene (92, 66 and 69 μM, respectively).*

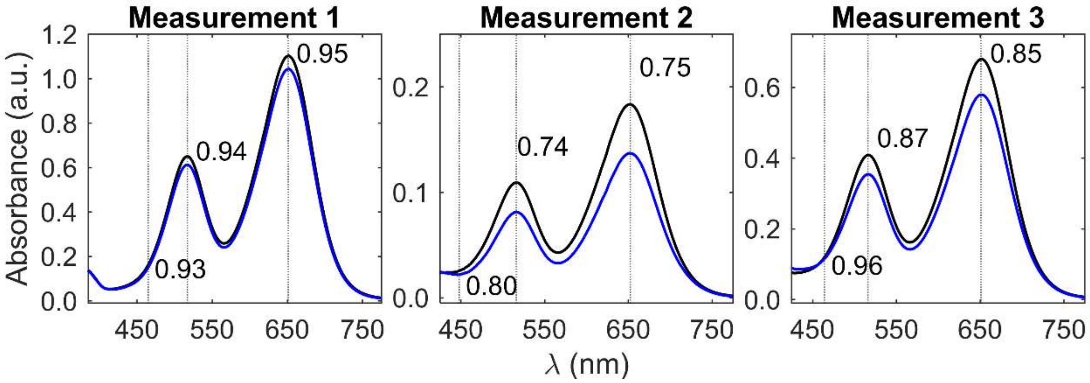

Figure S39. *Estimation of the stability of the UV-vis sample of* ***2a*** *in Toluene (92, 66 and 69 μM, respectively) by comparing the UV-vis spectrum before (black) and after (blue) the measurements under the starting conditions. The reported numbers indicate the ratio of absorbance at the isosbestic point and the two maxima between the two.*

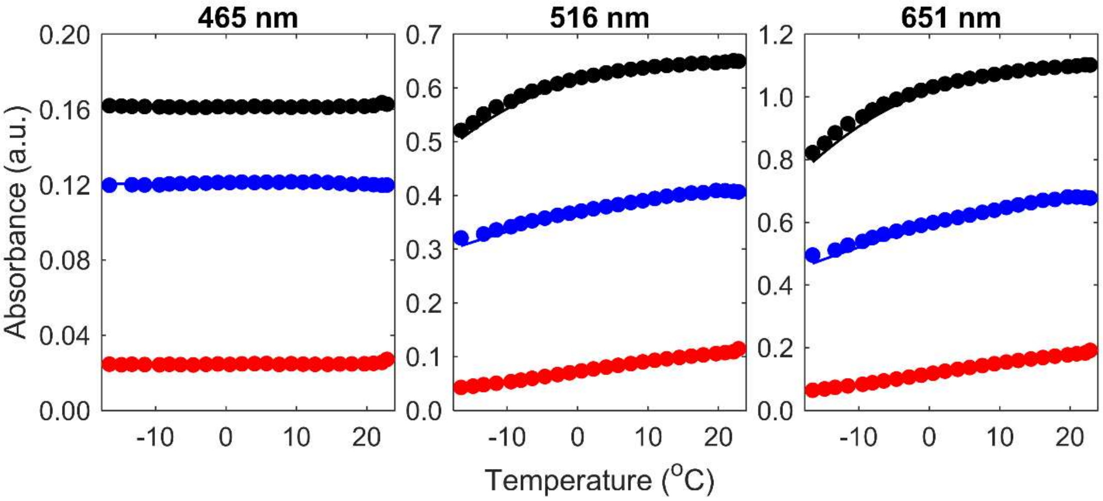

Figure S40. *Fitting of the isosbestic point and absorption maxima in the temperature dependent UV-vis spectra of **2a** in Toluene from **Figure S38**. Measurement 1: black; Measurement 2: red; Measurement 3: blue.*

Table S2. *Results of the fitting curves of the peak maxima shown of **2a** in Toluene in **Figure S40**.*

**Parameter**	**Measurement 1**		**Measurement 2**		**Measurement 3**		**Average**		**Total**
	517 nm	651 nm	516 nm	652 nm	516 nm	651 nm	516 nm	651 nm	
**ε** [M^{-1} cm^{-1}]	7092 ± 5	12043 ± 16	3480 ± 45	2088 ± 34	6397 ± 47	10798 ± 115	5656 ± 1917	8310 ± 5424	
**ΔH** [kJ mol^{-1}]	–58.29 ± 0.35	–60.81 ± 0.45	–56.66 ± 0.49	–51.99 ± 0.61	–32.81 ± 0.97	–35.54 ± 1.13	–49.25 ± 14.26	–49.45 ± 12.83	–115.6 ± 47.6
**ΔS** [J mol^{-1} K^{-1}]	–163.7 ± 1.3	–170.2 ± 1.7	–126.6 ± 2.1	–110.4 ± 2.7	–57.7 ± 3.9	–65.2 ± 4.6	–116.0 ± 53.8	–115.3 ± 52.7	–49.4 ± 12.1
**$\Delta G_{293 K}$** [kJ mol^{-1}]	–10.33	–10.93	–19.63	–19.58	–15.90	–16.45	–15.27 ± 4.65	–15.67 ± 4.40	–15.47 ± 4.06
**$\Delta G_{298 K}$** [kJ mol^{-1}]	–9.51	–10.08	–19.08	–18.94	–15.61	–16.12	–14.69 ± 4.78	–15.10 ± 4.58	–14.89 ± 4.20

4.2 Variable Temperature Nuclear Magnetic Resonance Spectroscopy

Below 233 K, the ^{1}H NMR resonance for N–CH_3 ceases to change in response to temperature. Using the temperature exhibiting the greatest separation as the low T limit (188 K), the rate of exchange for the dimer shuffling process was calculated from the following piecewise function:

$$k = \begin{cases} \pi(\delta v)^2 (w - w_f)^{-1} (2)^{-1/2} & if\ T > T_c \\ \pi(\delta v)(2)^{-1/2} & if\ T = T_c \\ \pi(w - w_0) & if\ T < T_c \end{cases}$$

Where T_c is the coalescence temperature (208 K), δv is the peak separation at the low T limit (35.8 Hz), w is the full-width at half-height of the resonance at T, w_0 is the full-width at half-height for the resonance at the low T limit (9.74 Hz) and w_f is the full-width at half-

height of the resonance at the high T limit (5.09 for T ≥ 233 K). At 203 K the peak widths cannot be accurately measured due to broadening and so it cannot be used for this calculation. This broadening of peak widths is also present at 208 K, however, as T_c = 208 K, peak widths are not required for the rate calculation at this temperature.

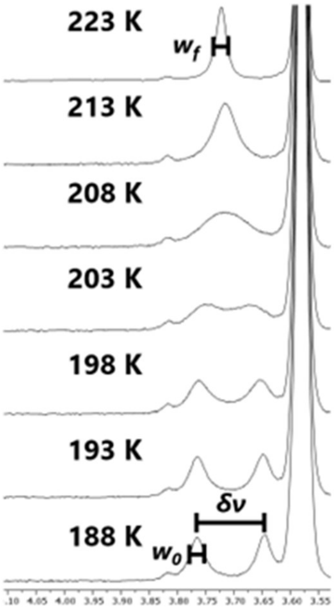

***Figure S41**. Variable temperature ^{1}H NMR data for **2a** in THF-d8. The fast exchange (223 K), coalescence (208 K) and slow exchange (188 K) regimes for the dimer shuffling process are visible at the stated temperatures. For full spectra see **Figure S44** and **Section 4.2.2**.*

***Table S3**.*

Temperature / K	*w / Hz*	*k*
233	5.09	$1.1x10^4$
223	7.07	$1.3x10^3$
213	14.45	$3.0x10^2$
208	-	$8.0x10^1$
198	14.66	$1.5x10^1$
193	10.56	2.6
188	9.74	0

The rate of exchange at 188 K is calculated to be 0 due to this being the lowest temperature reachable with the NMR spectrometers available. This datum was excluded from the Eyring plot as the ln(*k*) of 0 is undefined.

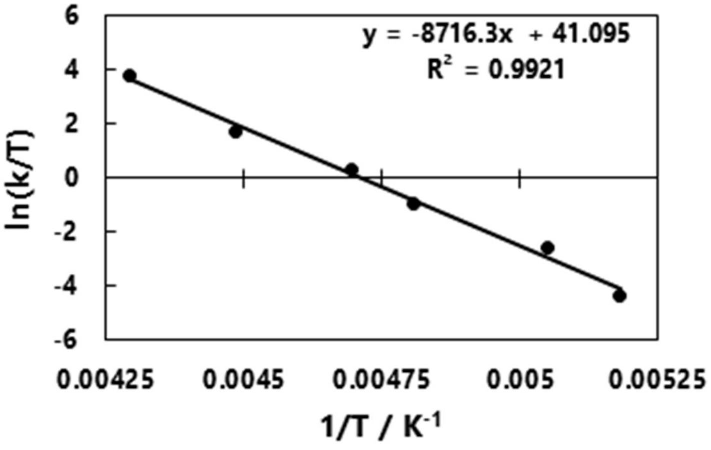

***Figure S42**. Eyring plot generated from VT NMR data of **2a**.*

The Eyring plot calculated using these data gives a linear relationship which can be expressed in the form:

$$ln\frac{k}{T} = \frac{-\Delta H^{\ddagger}}{RT} + ln\frac{\kappa k_b}{h} + \frac{\Delta S^{\ddagger}}{R}$$

allowing for the extraction of enthalpy ($\Delta H^{\ddagger}$ = 72.1±3.2 kJ·mol^{-1}) and entropy ($\Delta S^{\ddagger}$ = 0.144±0.015 kJ·mol^{-1}).

Error propagation was employed using the following equations:

$$\sigma_{\Delta H} = \frac{R \cdot \sigma_{slope}}{(1000J \cdot kJ^{-1})}$$

$$\sigma_{\Delta H} = \frac{R \cdot \sigma_{y-intercept}}{(1000J \cdot kJ^{-1})}$$

$$\sigma_{\Delta G} = \sqrt{\sigma_{\Delta H}{}^2 + \sigma_{\Delta H}{}^2}$$

4.2.1 Stacked VT-NMR Spectral Data

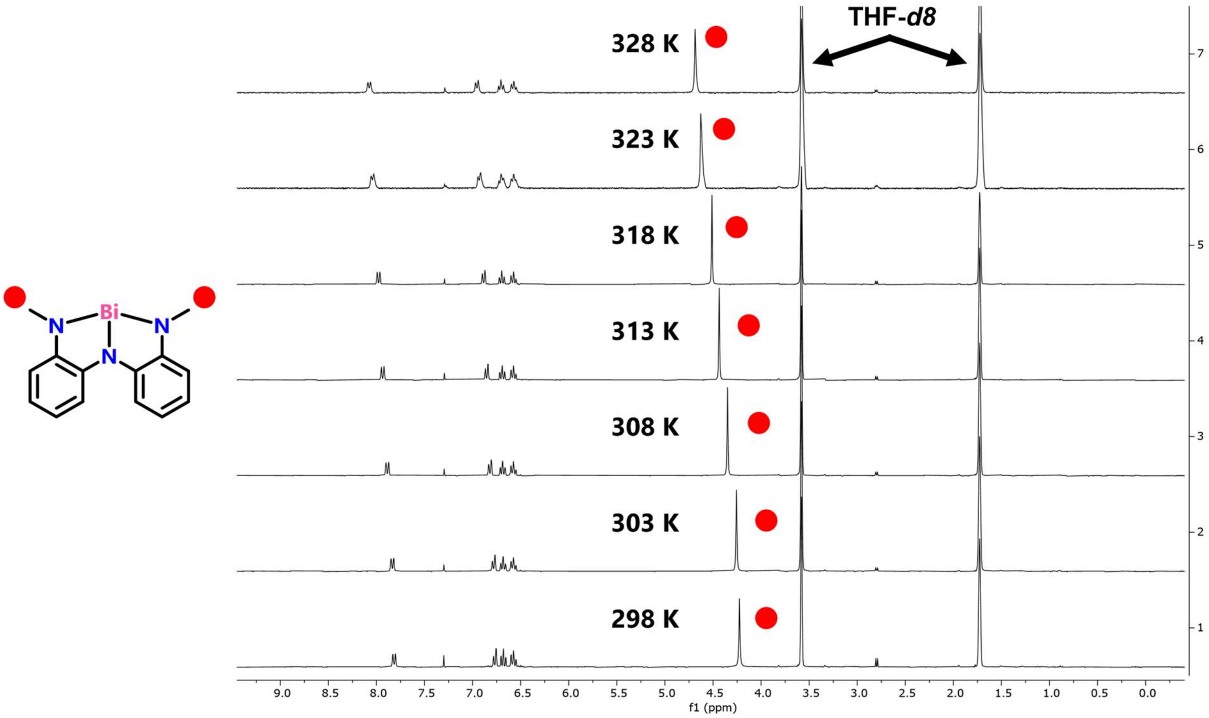

Figure S43. *Stacked VT-NMR spectra of compound* ***2a*** *(tetrahydrofuran-d8, 300 MHz). Temperatures are ordered from highest (7 on the vertical axis – 328K) to lowest (1 – 298 K) in 5 K increments. Note the downfield drift of the* $N–CH_3$ *signal from 4.48 ppm (298 K) to 4.68 ppm (328 K). See below (**Figure S49** to **Figure S55** in **Section 4.2.2**) for individual spectra.*

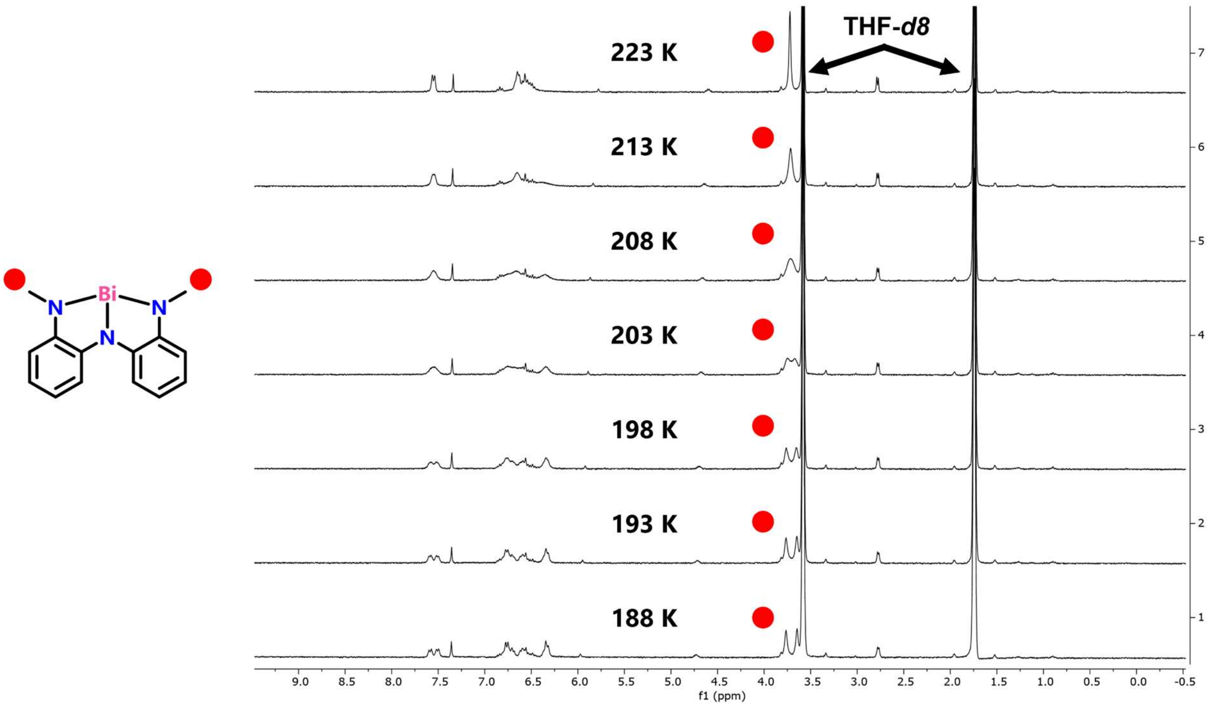

Figure S44. *Stacked VT-NMR spectra of compound* ***2a*** *(tetrahydrofuran-d8, 300 MHz). Temperatures are ordered from highest (7 on the vertical axis – 223K) to lowest (1 – 188 K) in 5 K increments. Protio ligand impurity is responsible for peaks observed at 2.80 ppm, 5.97-5.78 ppm, and 4.72-4.61 ppm (N–CH_3, central amine and terminal amines, respectively). See below (**Figure S63** to **Figure S69** in **Section 4.2.2**) for individual spectra.*

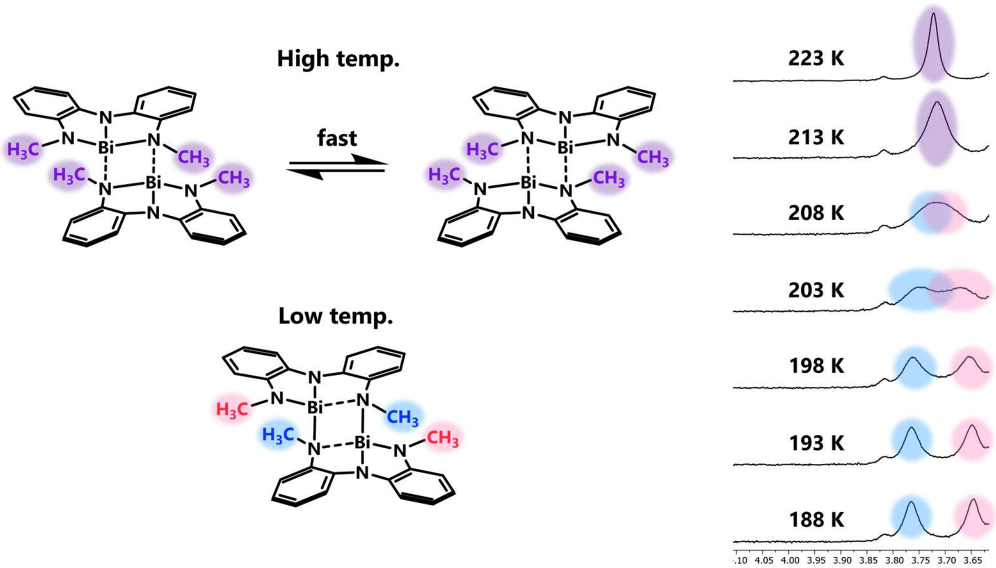

Figure S45. *Blown-up region (4.10 ppm to 3.60 ppm) of* ***Figure S44***. *Above the coalescence temperature, 208 K, the dimer side-to-side/shuffling motion is faster than the NMR time scale, and a single N–CH_3 peak is observed via ^{1}H-NMR. Loss of symmetry in NMR is observed at the ligand N–CH_3 protons upon cooling below 208 K.*

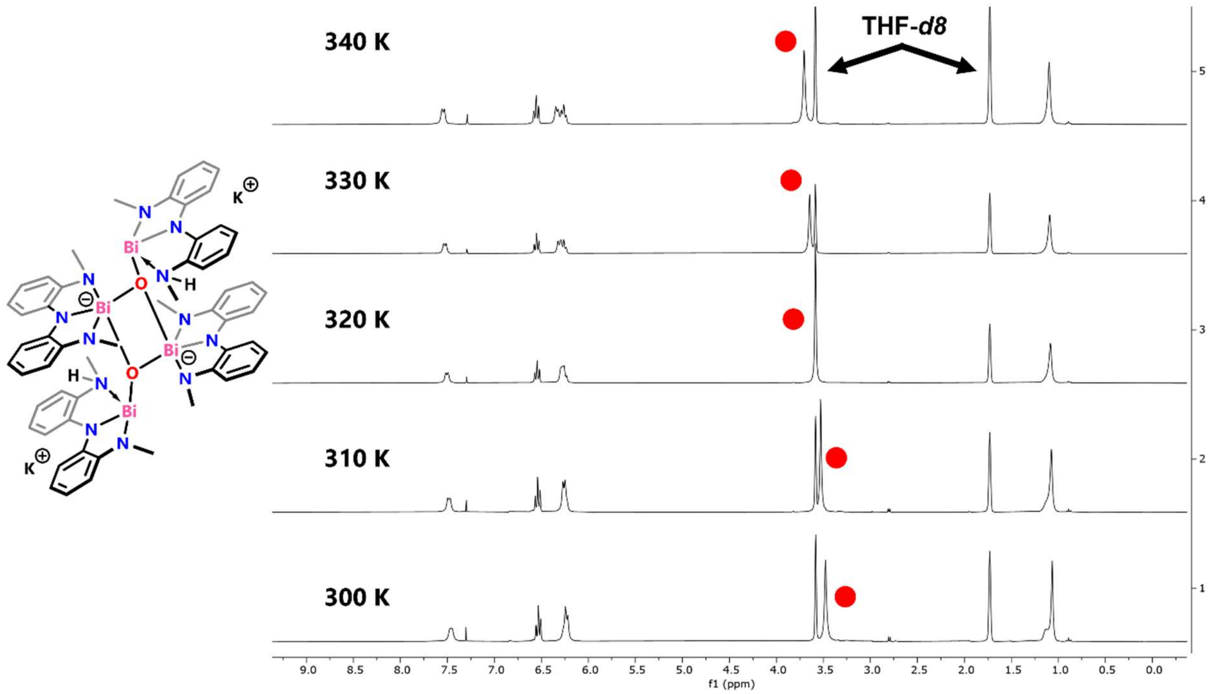

Figure S46. *Stacked VT-NMR spectra of compound **4** (tetrahydrofuran-d8, 300 MHz). Note the downfield drift of the N–CH_3 signal from 3.48 ppm (300 K) to 3.71 ppm (340 K). The red circles denote resonances associated with the ligand N–CH_3 protons. See below (**Section 4.2.3**) for full spectra.*

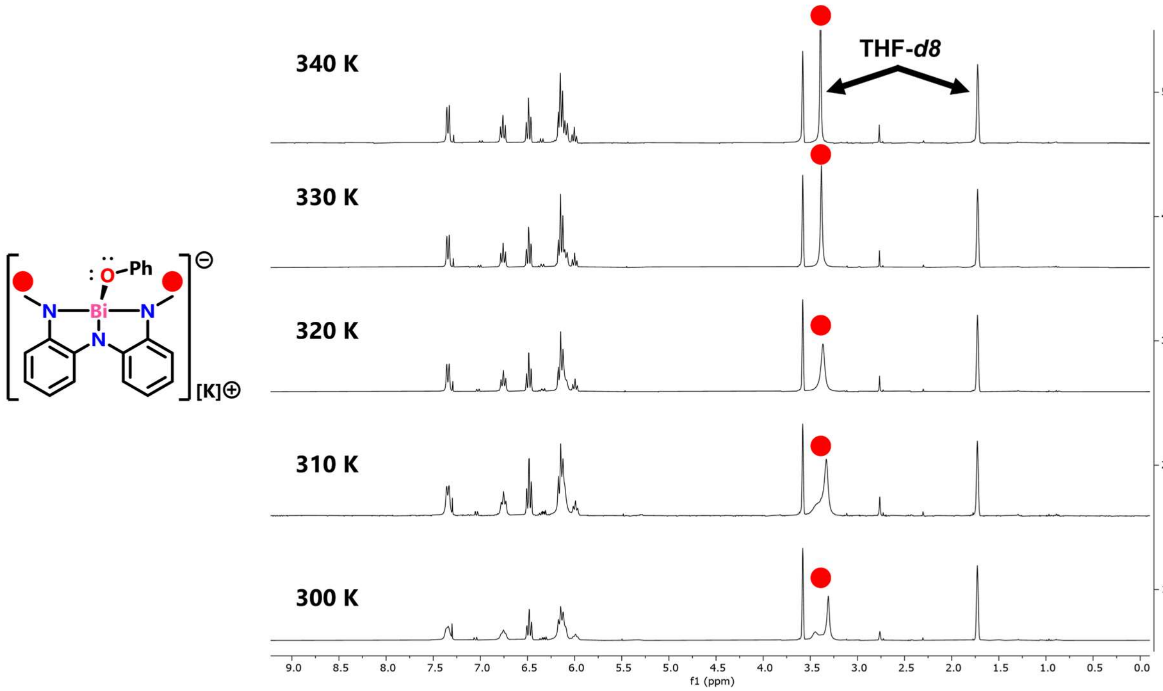

Figure S47. *Stacked ^1H VT-NMR spectra (THF-d8, 300 MHz) of **8** from 300 K to 340 K. The red circles denote protons associated with the N-CH_3 groups. Note that the N–CH_3 signal does not*

*drift downfield past the THF-d8 peak, unlike in ^{1}H VT-NMR spectra gathered for **2a** and **4**. See below (**Section 4.2.4**) for full spectra.*

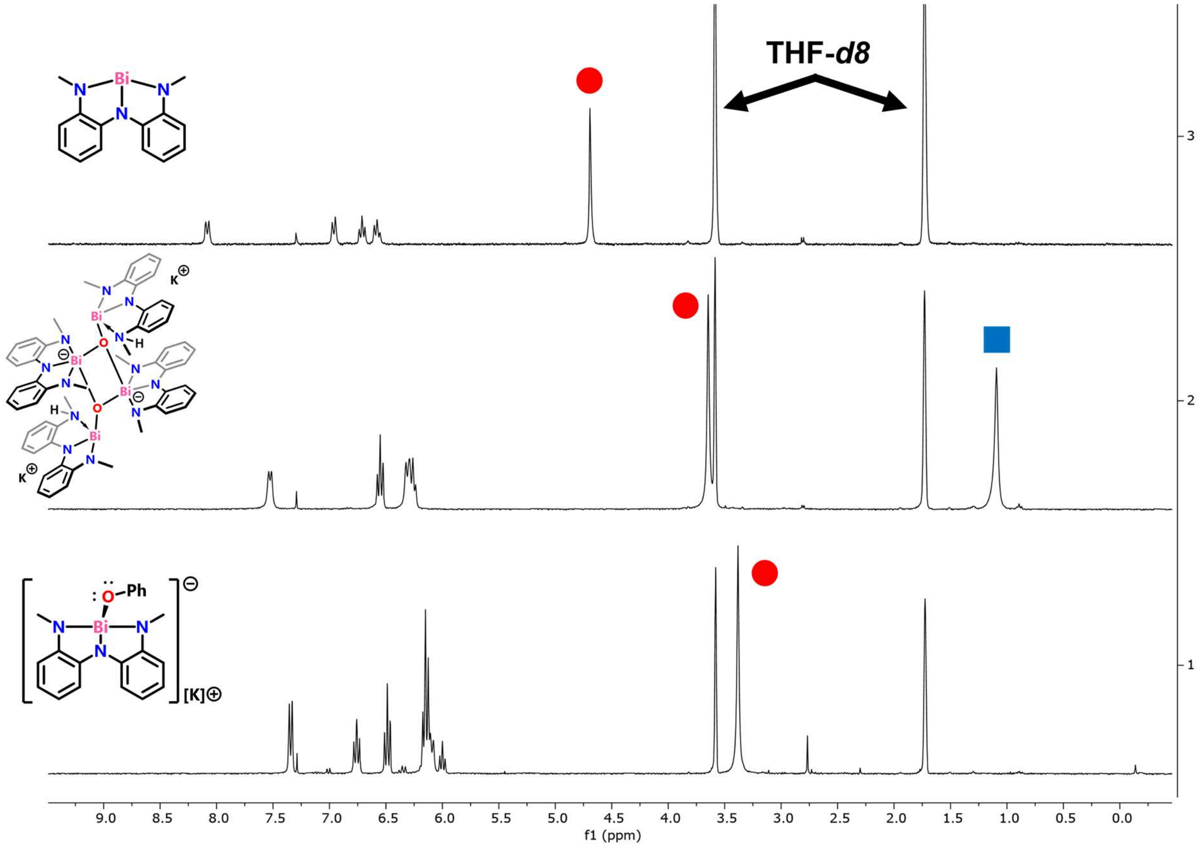

***Figure S48**. Stacked ^{1}H VT-NMR spectra (THF-d8, 300 MHz) for **2a** (top), **4** (middle) and **8** at 330 K. The red circles denote resonances associated with the ligand N–CH_3 protons. The blue square represents resonances due to polyisobutylene.*

4.2.2 Individual VT-NMR Spectra for **2a**

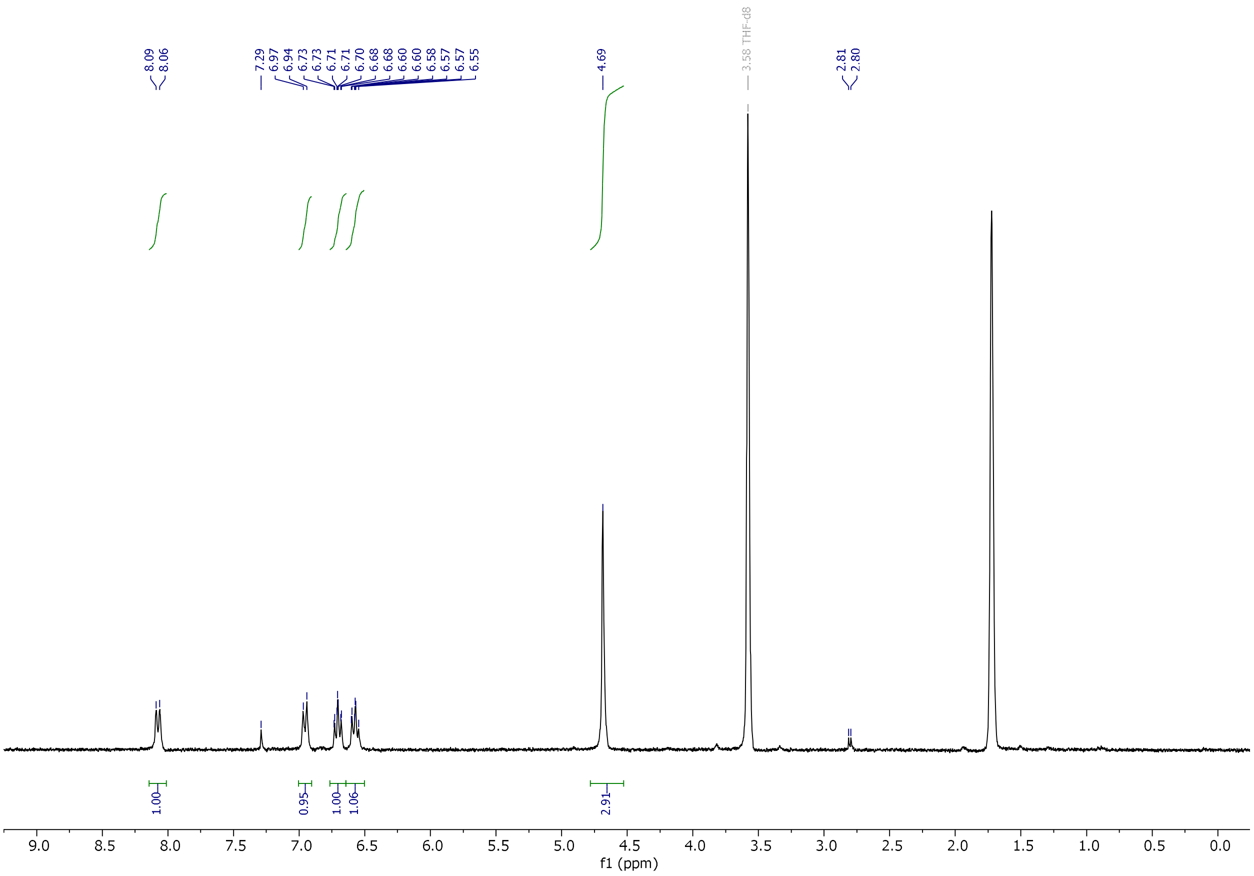

***Figure S49**. ^{1}H NMR spectrum (THF-d8, 300 MHz) of **2a** at 328 K. The doublet at 2.80 ppm is identified as the free ligand N–CH_3 proton signal.*

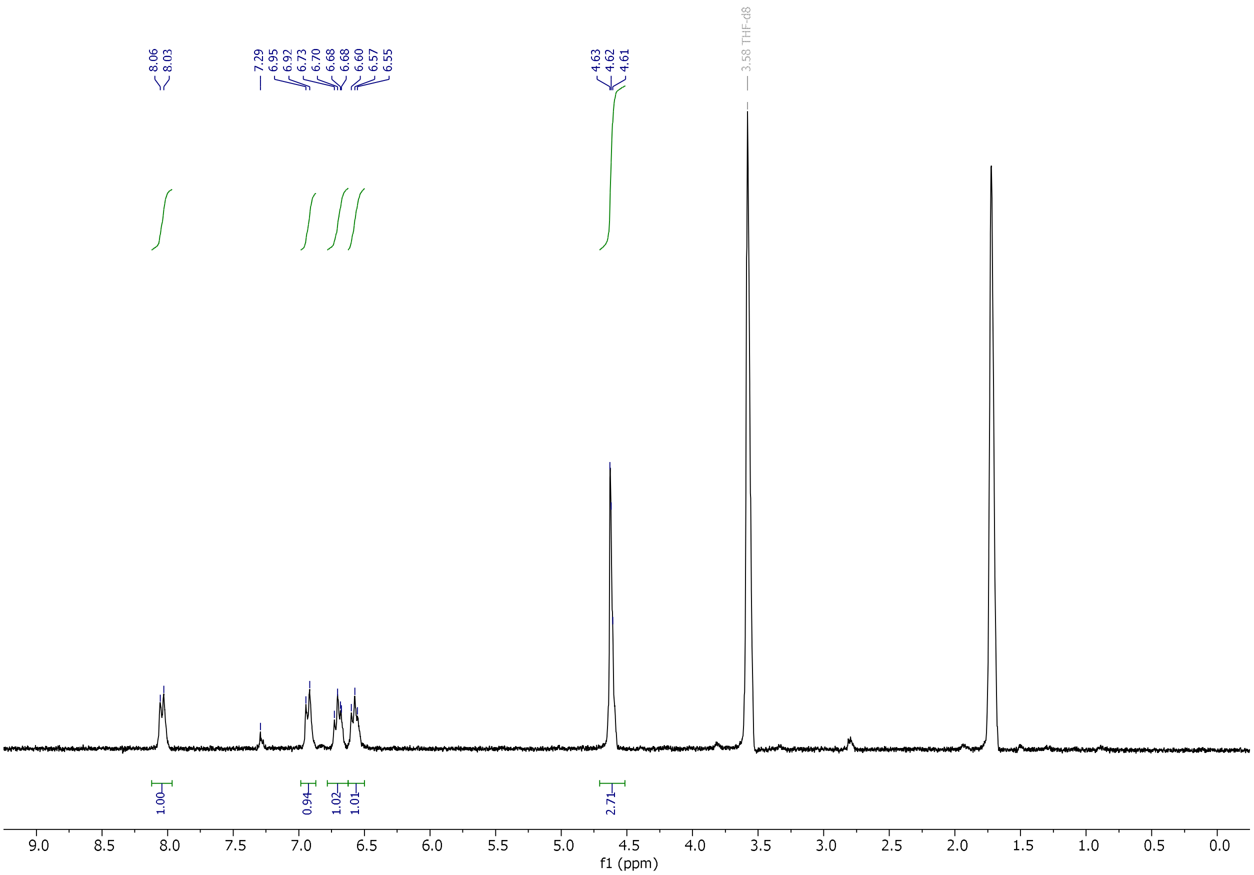

Figure S50. *^{1}H NMR spectrum (THF-d8, 300 MHz) of **2a** at 323 K. The doublet at 2.80 ppm is identified as the free ligand N–CH_3 proton signal.*

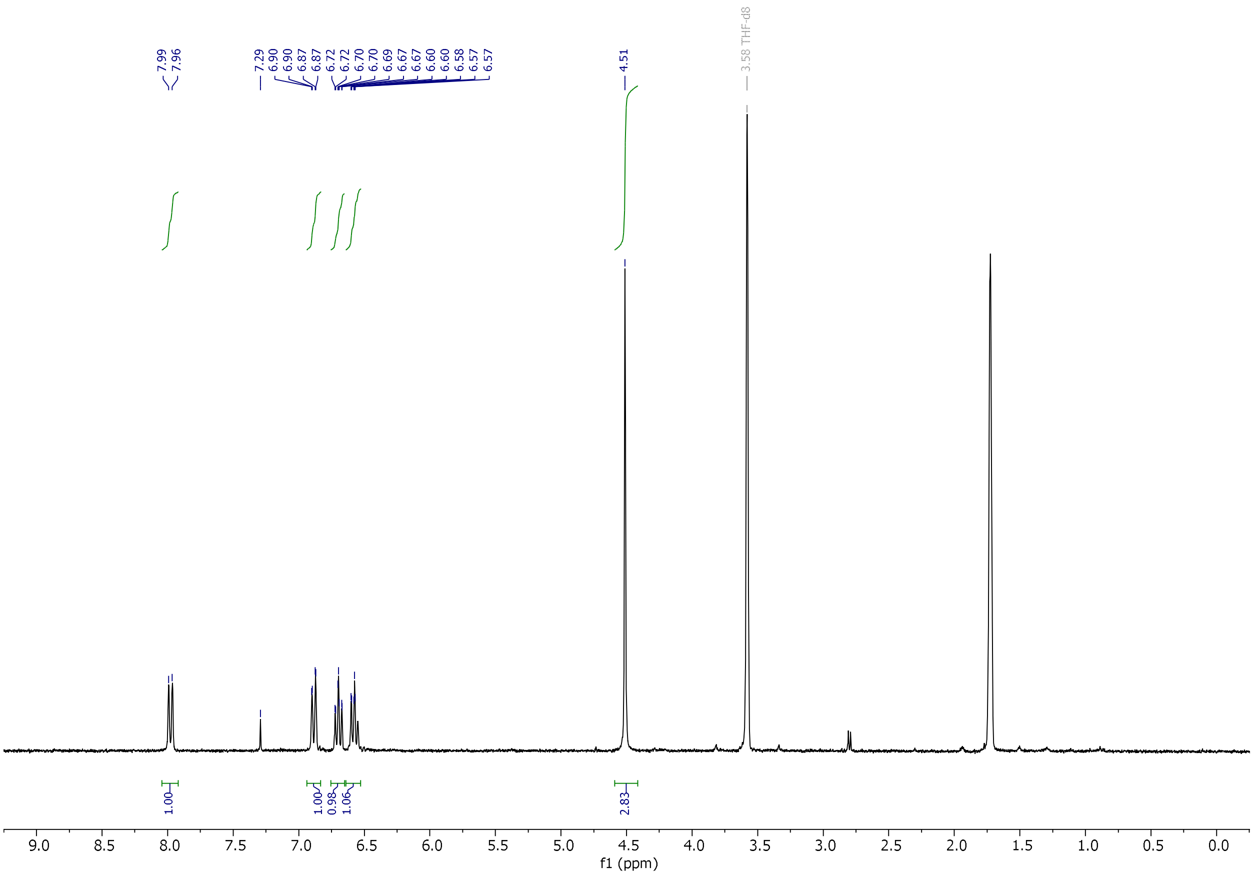

Figure S51. *^{1}H NMR spectrum (THF-d8, 300 MHz) of* ***2a*** *at 318 K. The doublet at 2.80 ppm is identified as the free ligand N–CH_3 proton signal.*

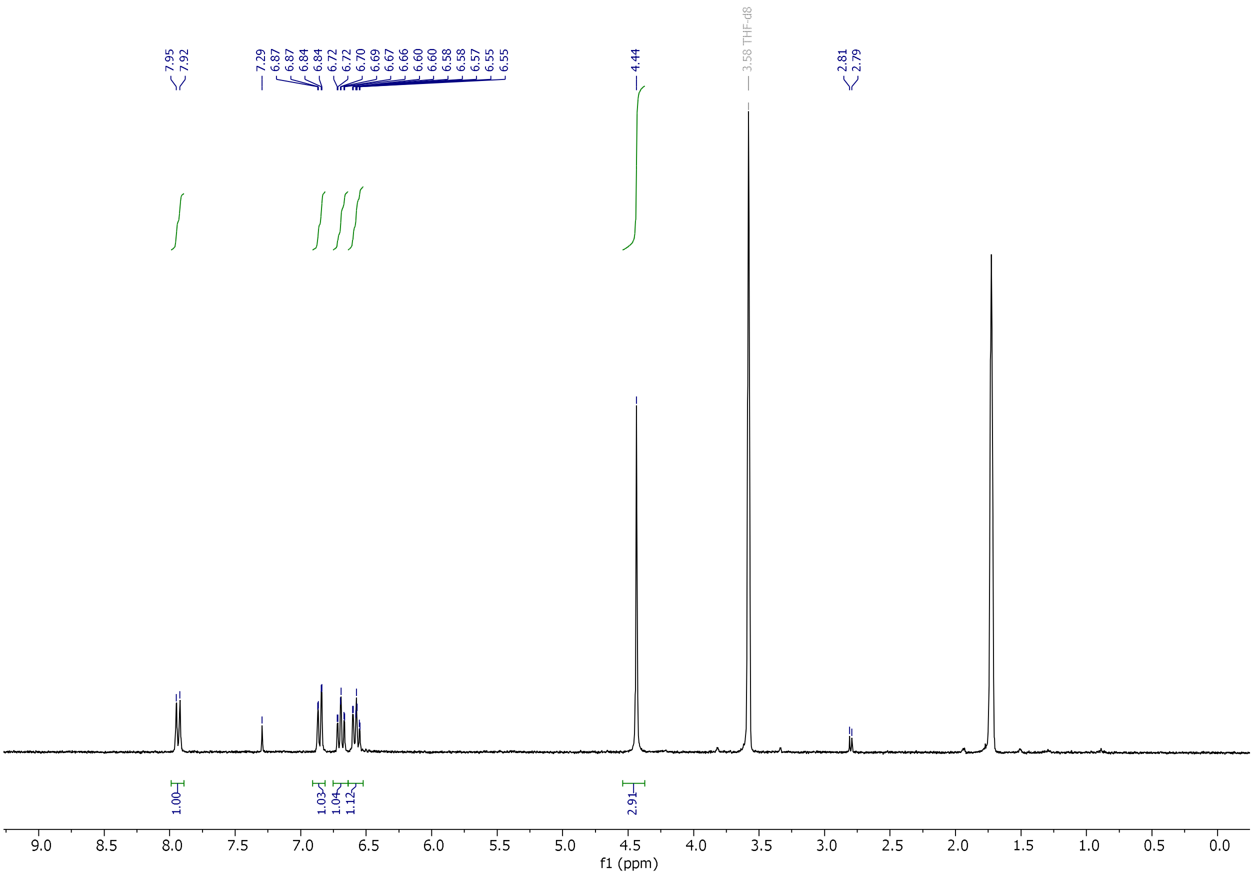

Figure S52. *^{1}H NMR spectrum (THF-d8, 300 MHz) of* ***2a*** *at 313 K. The doublet at 2.80 ppm is identified as the free ligand N–CH_3 proton signal.*

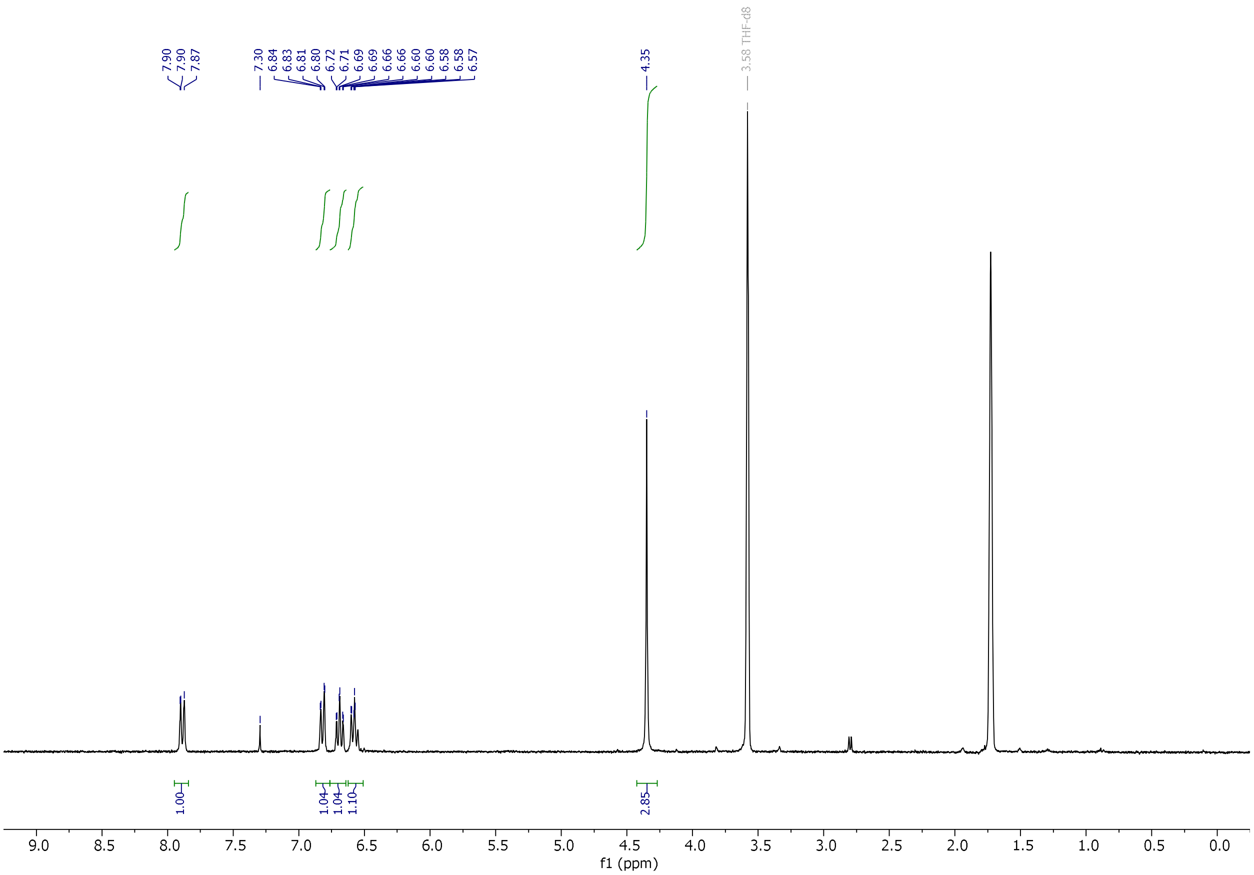

Figure S53. *^{1}H NMR spectrum (THF-d8, 300 MHz) of* ***2a*** *at 308 K. The doublet at 2.80 ppm is identified as the free ligand N–CH_3 proton signal.*

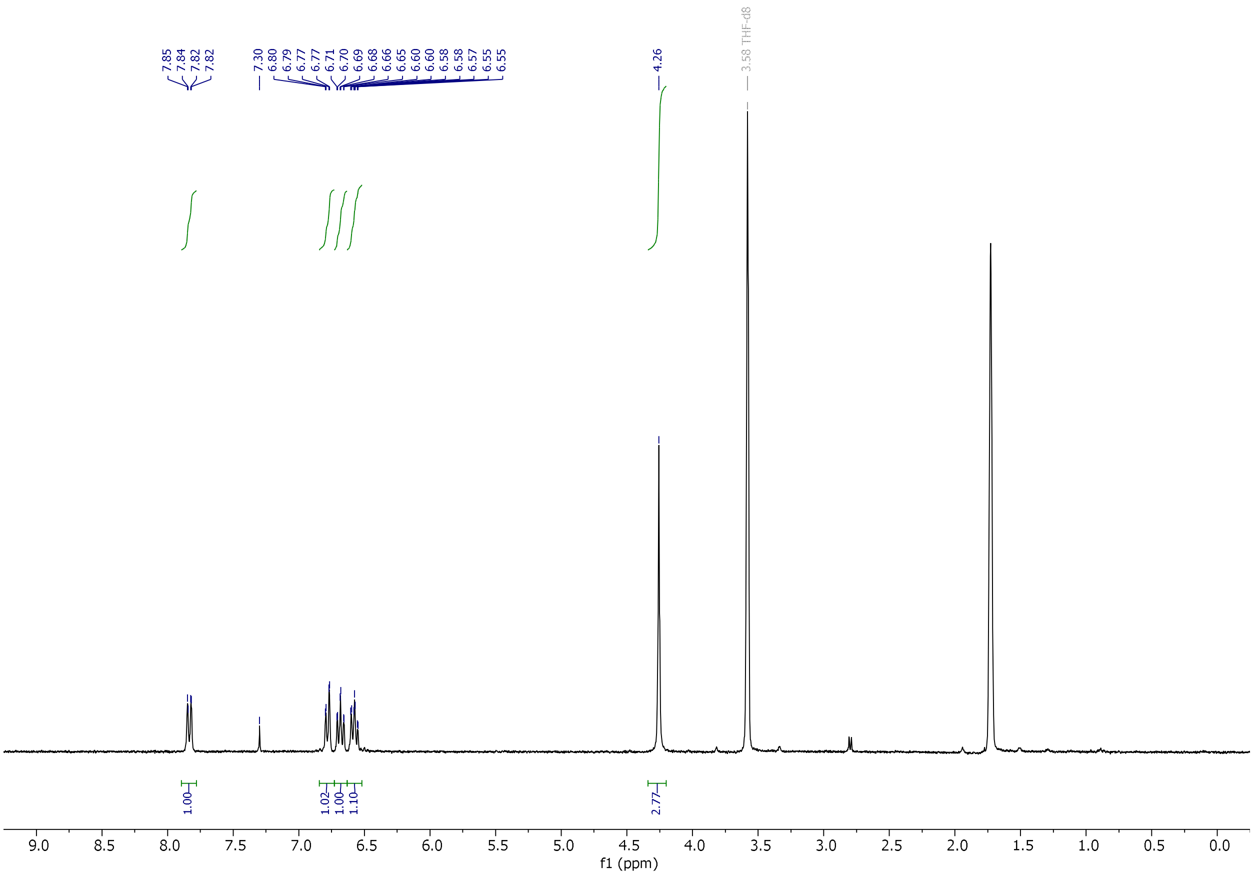

Figure S54. *^{1}H NMR spectrum (THF-d8, 300 MHz) of* ***2a*** *at 303 K. The doublet at 2.80 ppm is identified as the free ligand N–CH_3 proton signal.*

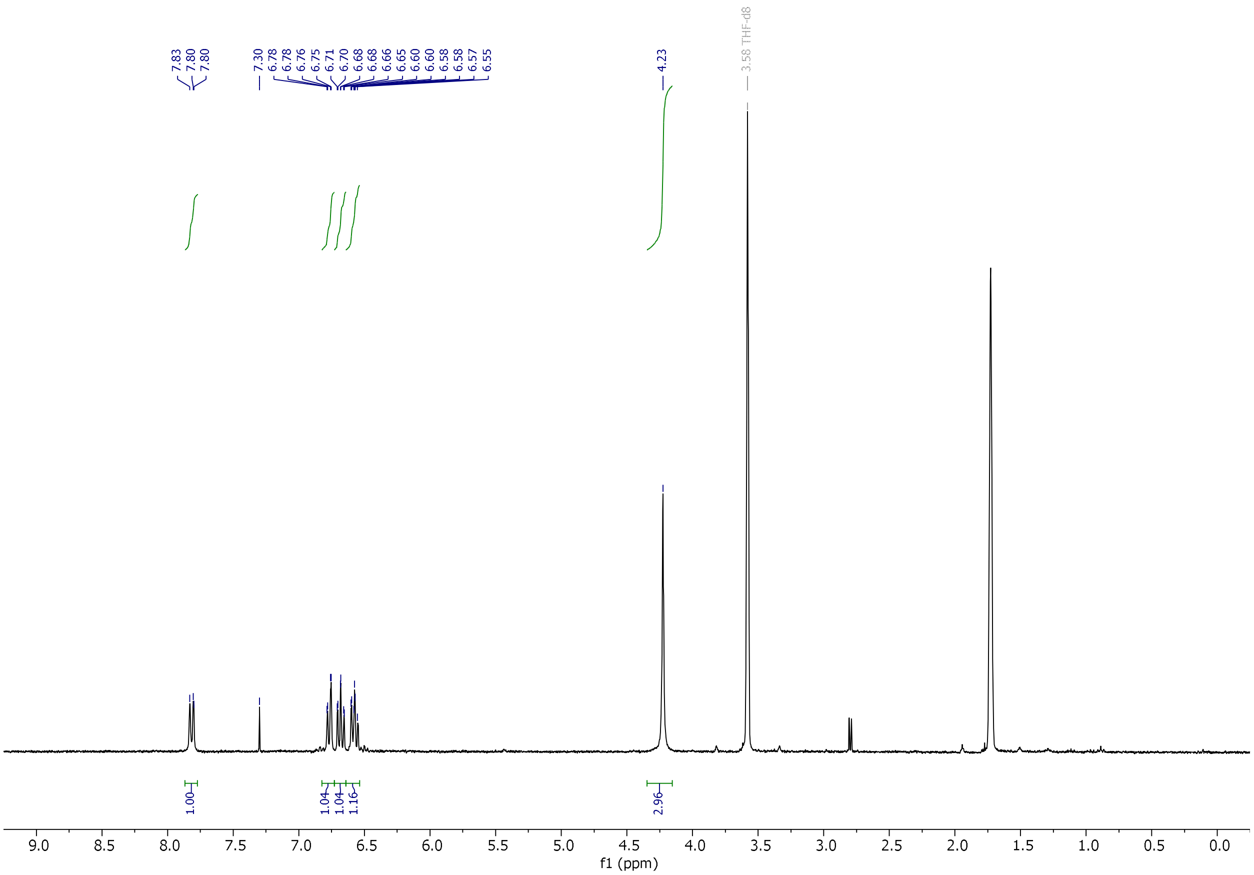

Figure S55. *^{1}H NMR spectrum (THF-d8, 300 MHz) of **2a** at 298 K. The doublet at 2.80 ppm is identified as the free ligand N–CH_3 proton signal.*

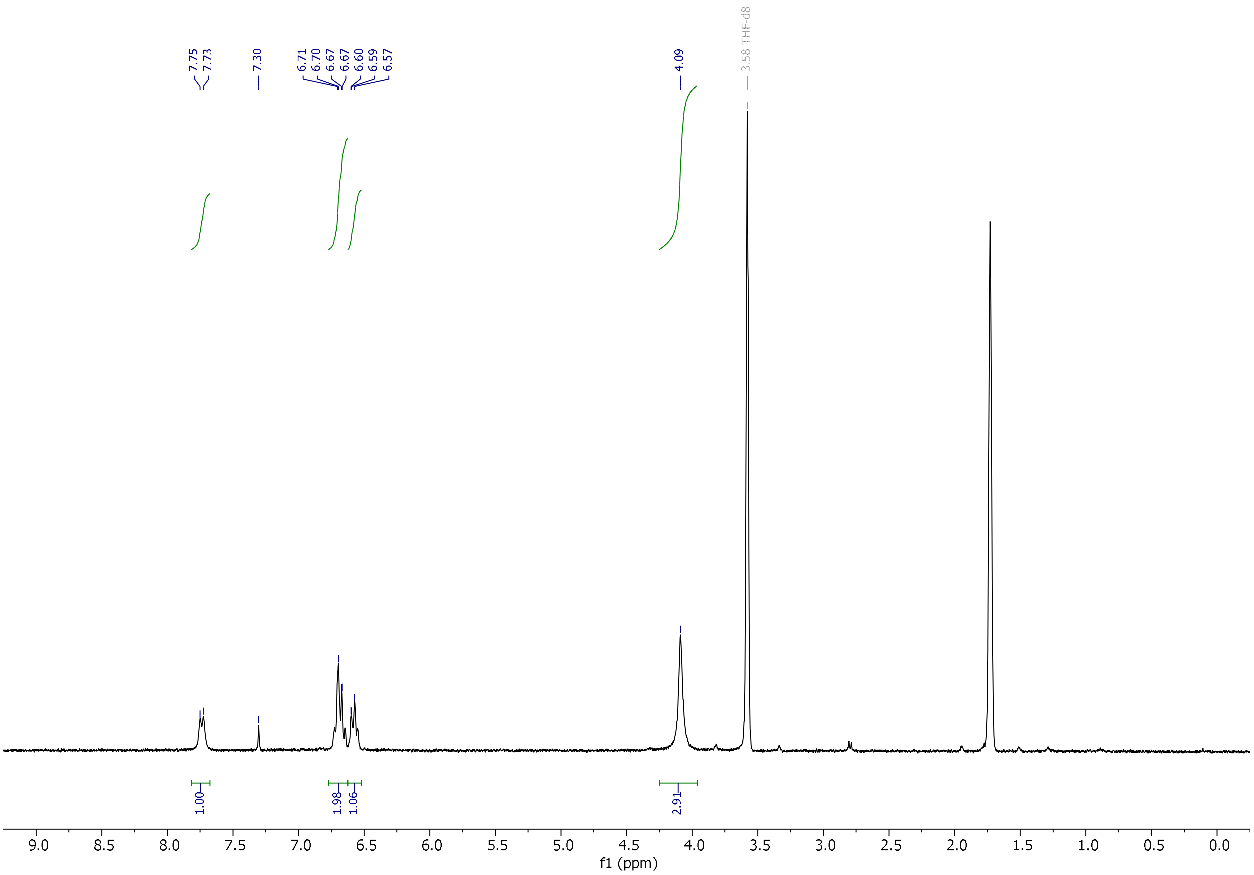

Figure S56. *^{1}H NMR spectrum (THF-d8, 300 MHz) of* ***2a*** *at 293 K. The doublet at 2.80 ppm is identified as the free ligand N–CH_3 proton signal.*

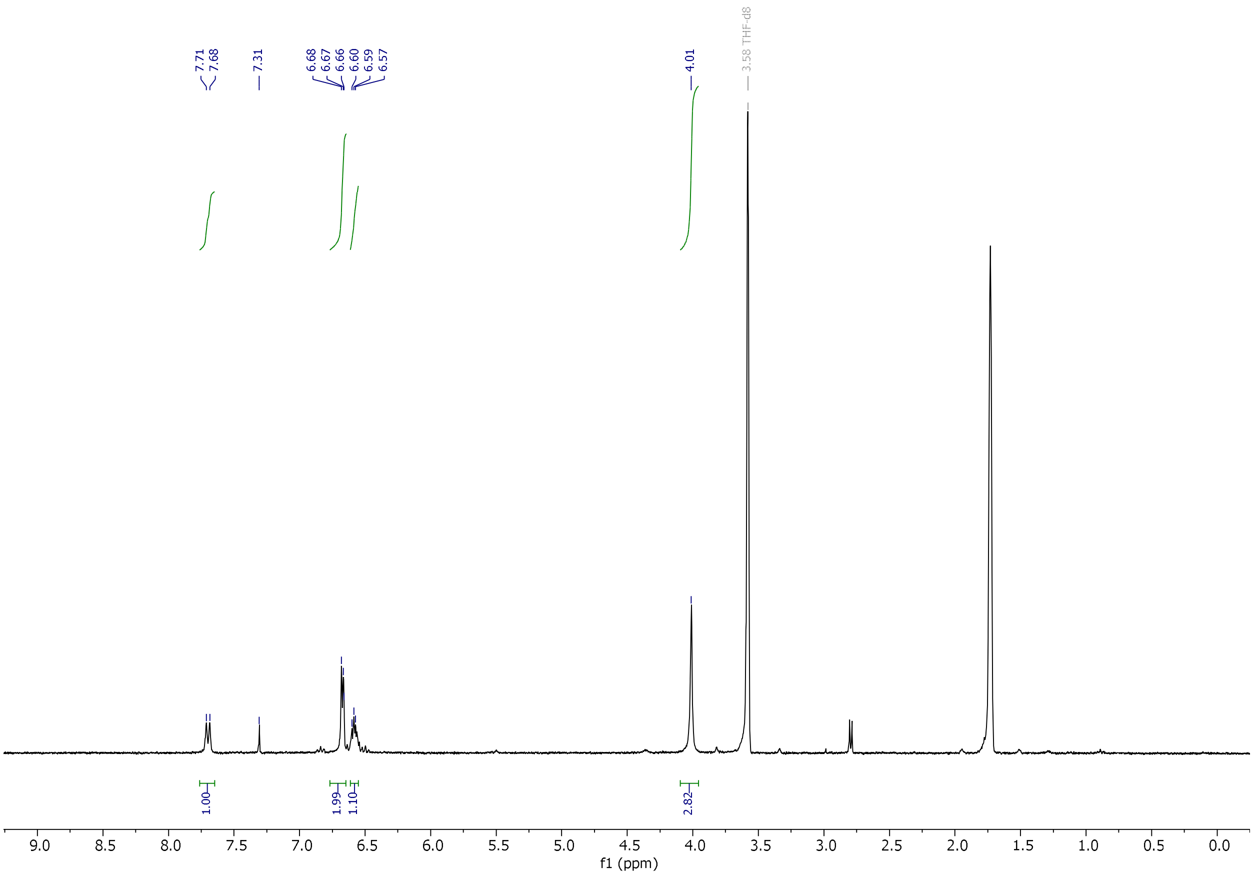

Figure S57. *^{1}H NMR spectrum (THF-d8, 300 MHz) of **2a** at 283 K. The doublet at 2.80 ppm is identified as the free ligand N–CH_3 proton signal.*

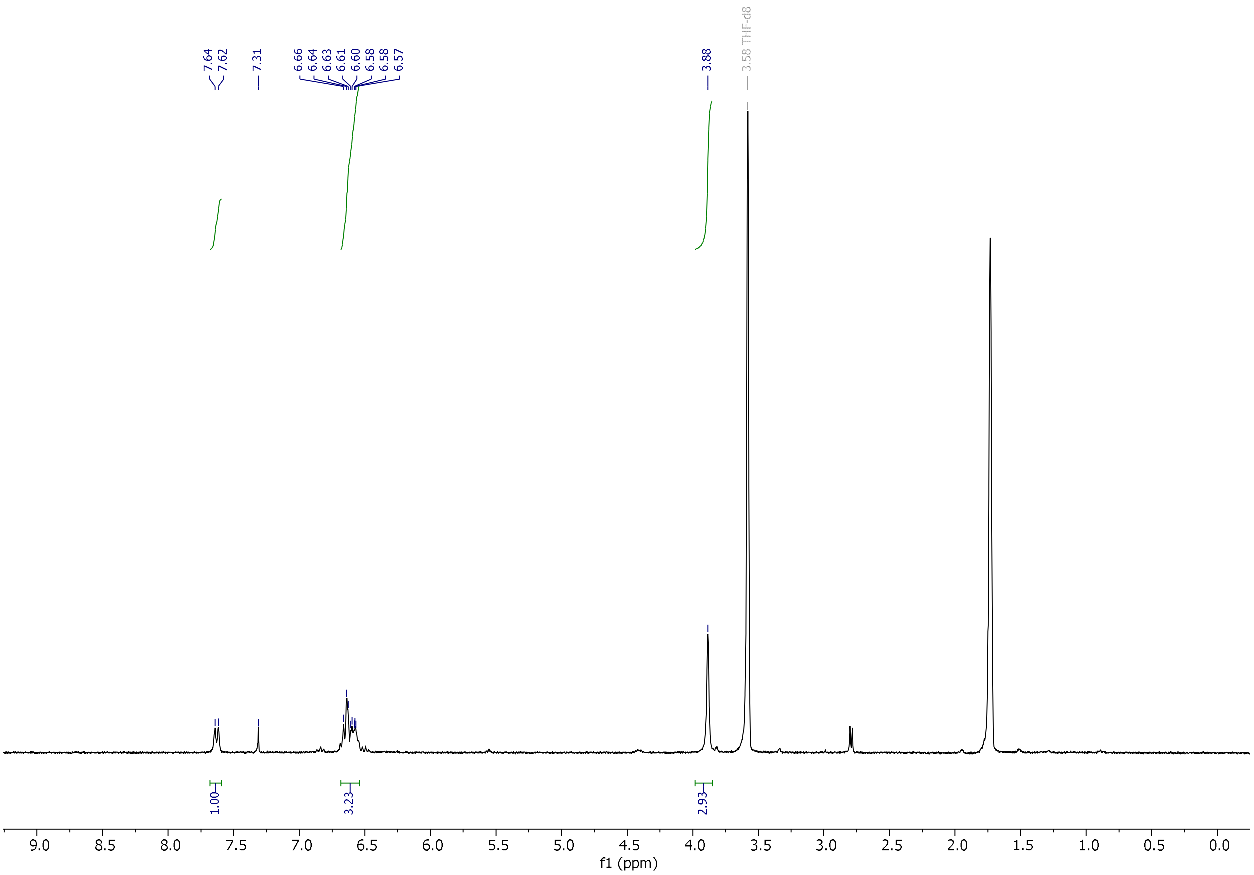

Figure S58. *^{1}H NMR spectrum (THF-d8, 300 MHz) of* ***2a*** *at 273 K. The doublet at 2.80 ppm is identified as the free ligand N–CH_3 proton signal.*

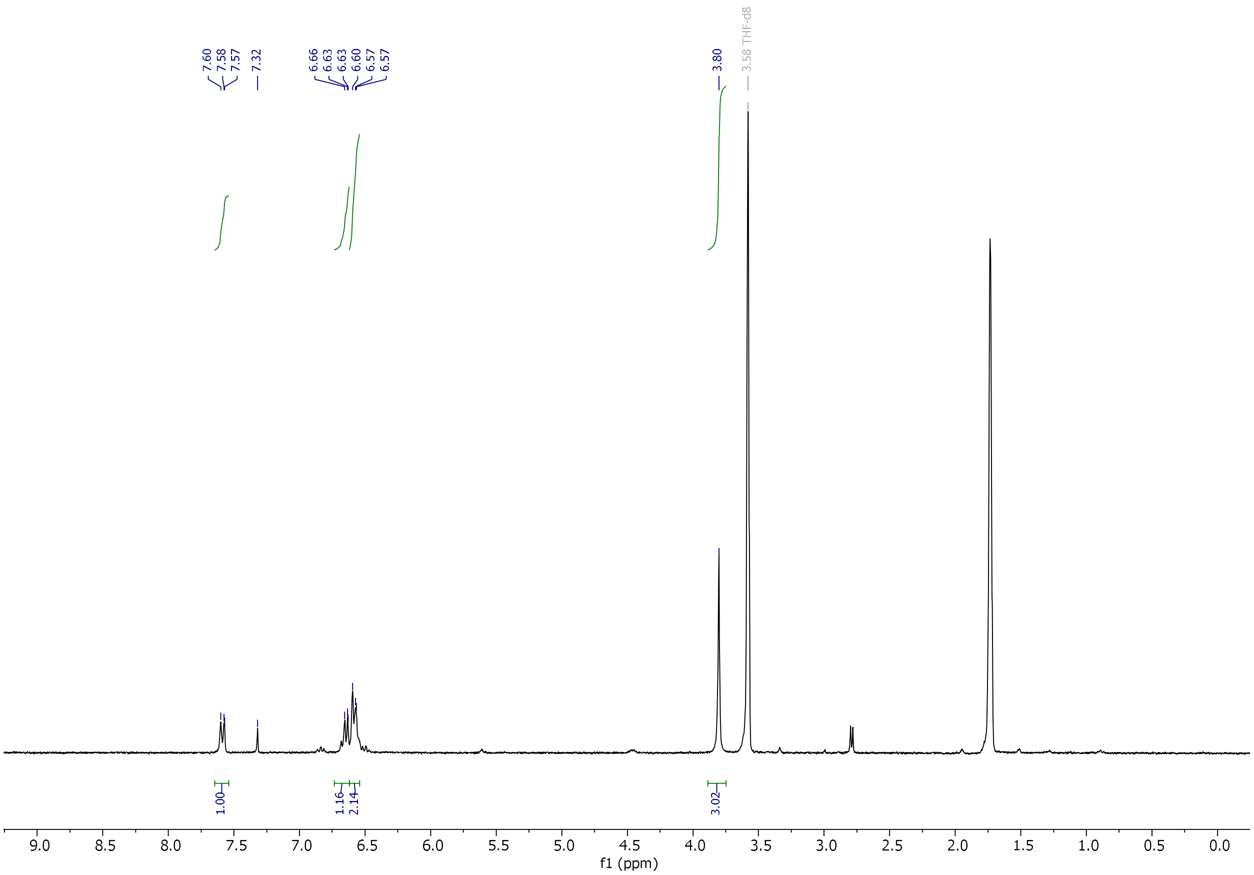

Figure S59. *^{1}H NMR spectrum (THF-d8, 300 MHz) of **2a** at 263 K. The doublet at 2.80 ppm is identified as the free ligand N–CH_3 proton signal.*

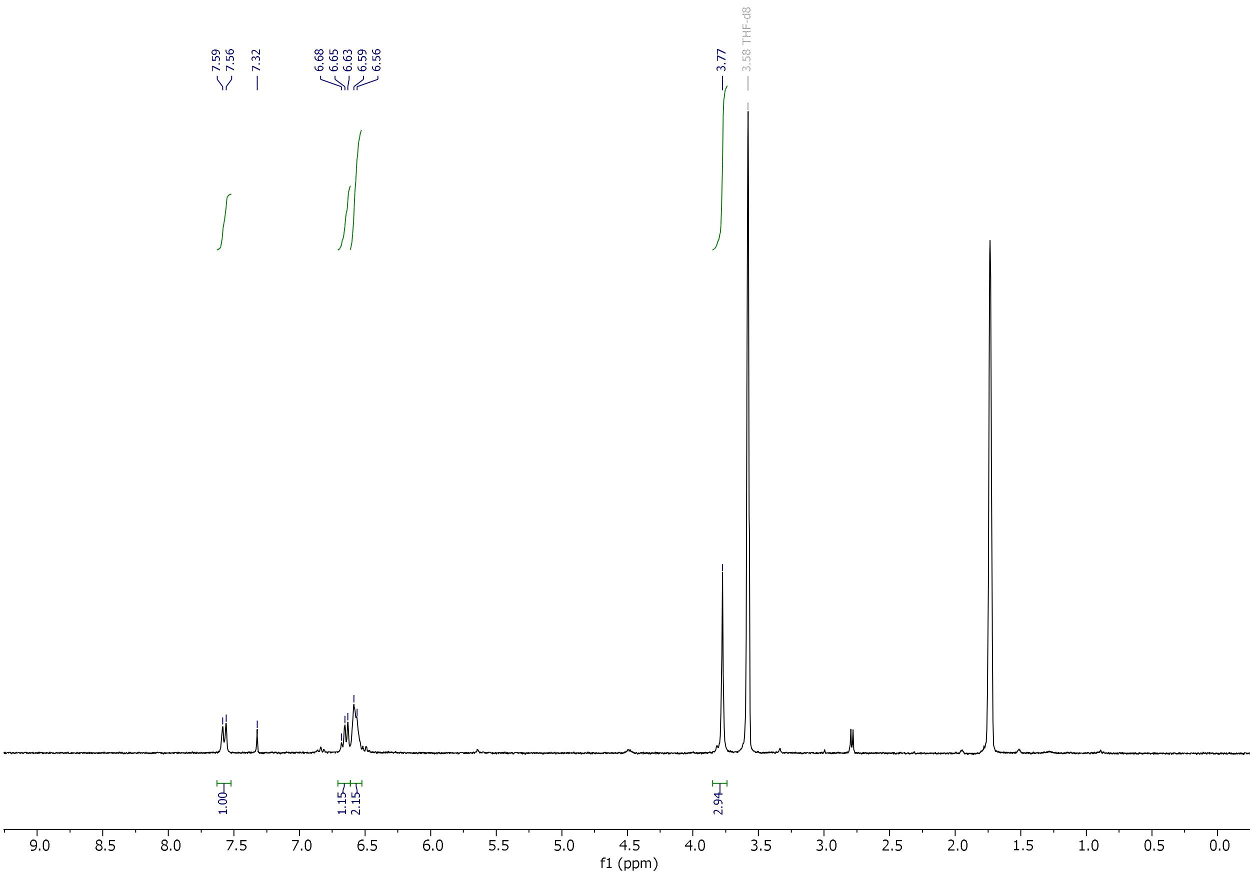

Figure S60. *^{1}H NMR spectrum (THF-d8, 300 MHz) of **2a** at 253 K. The doublet at 2.80 ppm is identified as the free ligand N–CH_3 proton signal.*

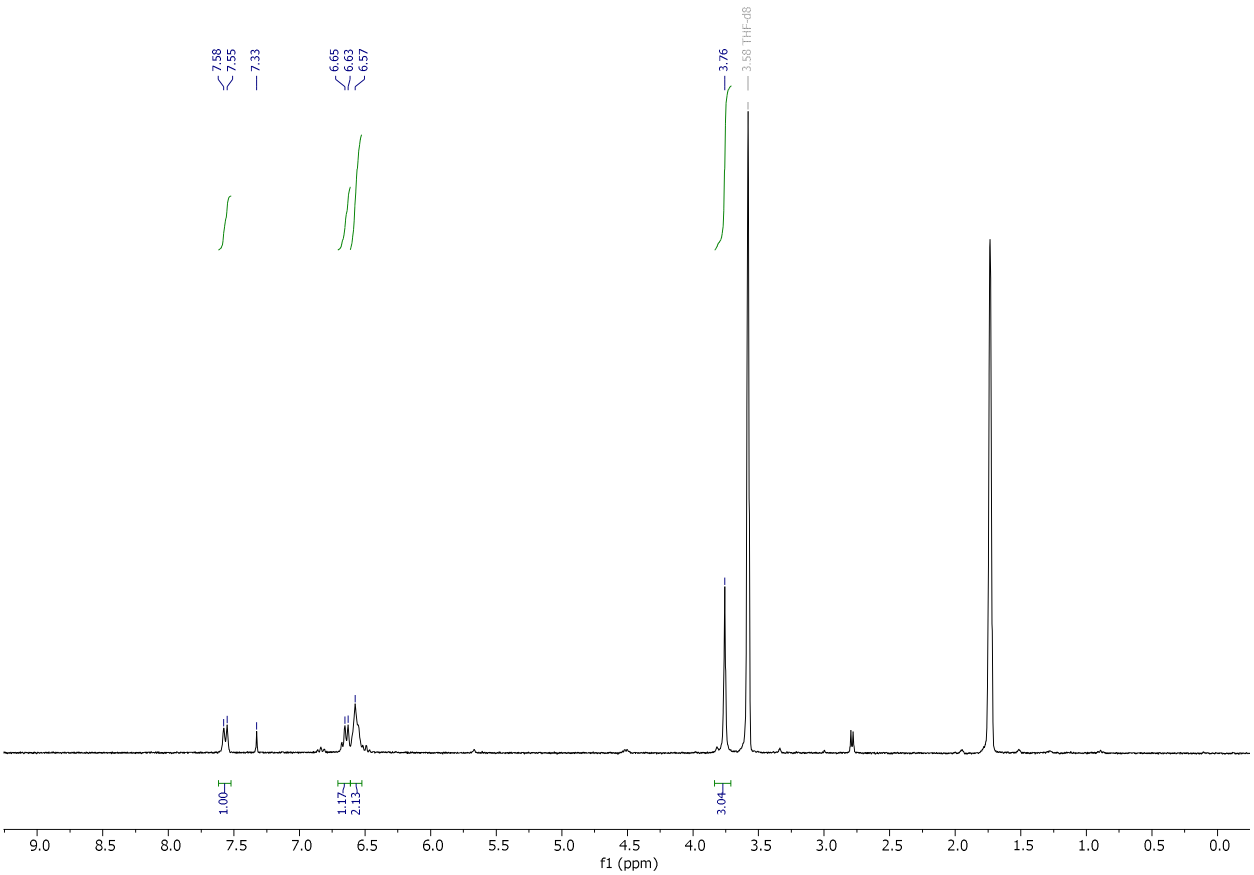

Figure S61. ^{1}H NMR spectrum (THF-d8, 300 MHz) of **2a** at 243 K. The doublet at 2.80 ppm is identified as the free ligand N–CH_3 proton signal.

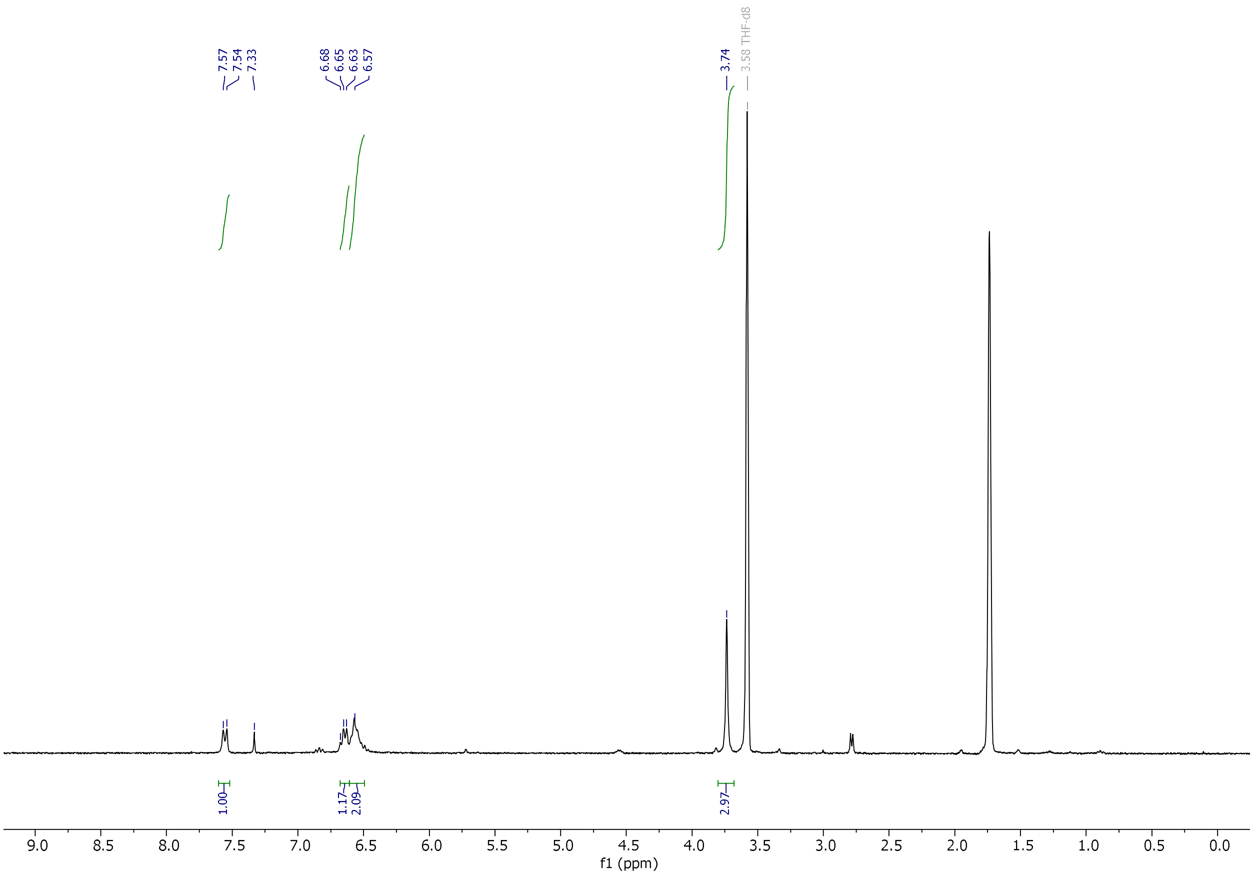

Figure S62. *^{1}H NMR spectrum (THF-d8, 300 MHz) of* ***2a*** *at 233 K. The doublet at 2.80 ppm is identified as the free ligand N–CH_3 proton signal.*

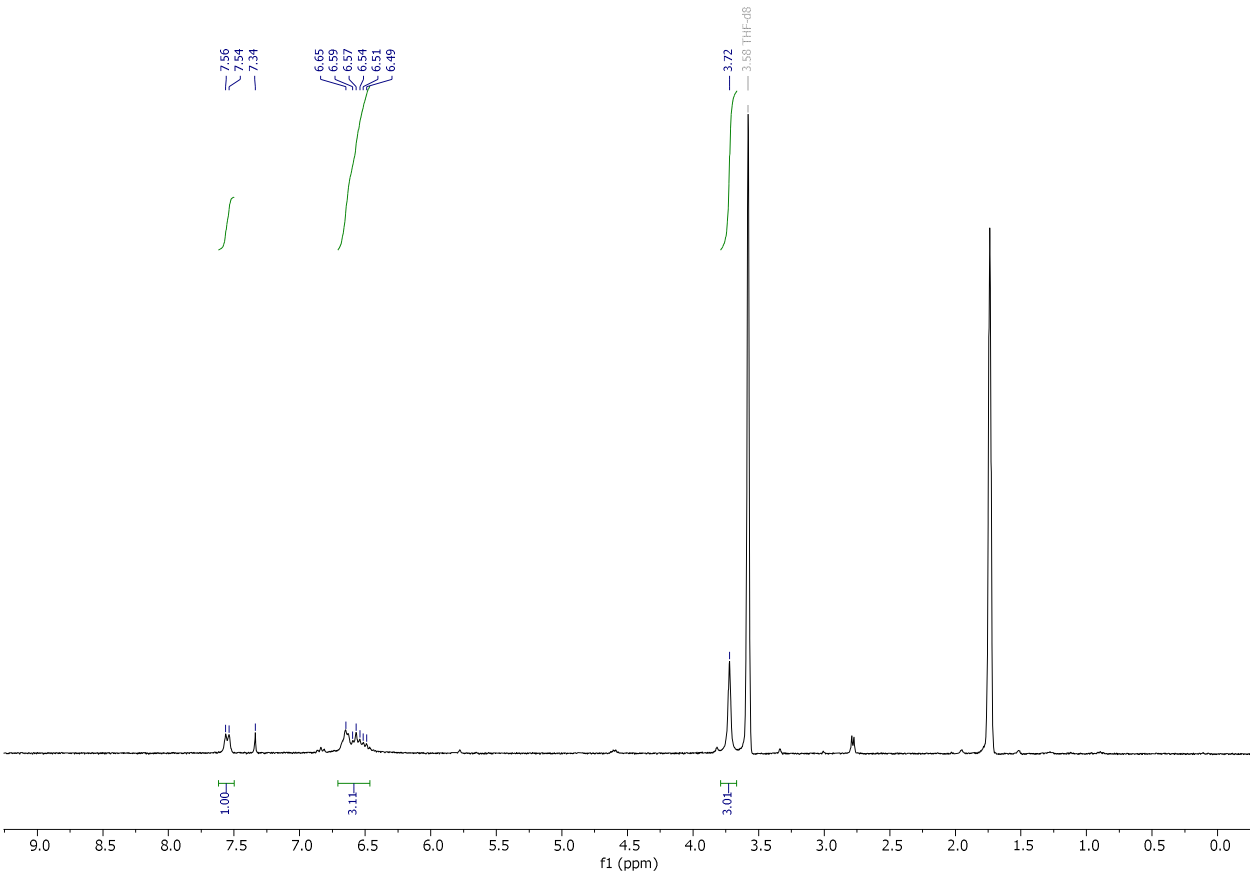

Figure S63. ^{1}H NMR spectrum (THF-d8, 300 MHz) of **2a** at 223 K. The doublet at 2.80 ppm is identified as the free ligand N–CH_3 proton signal.

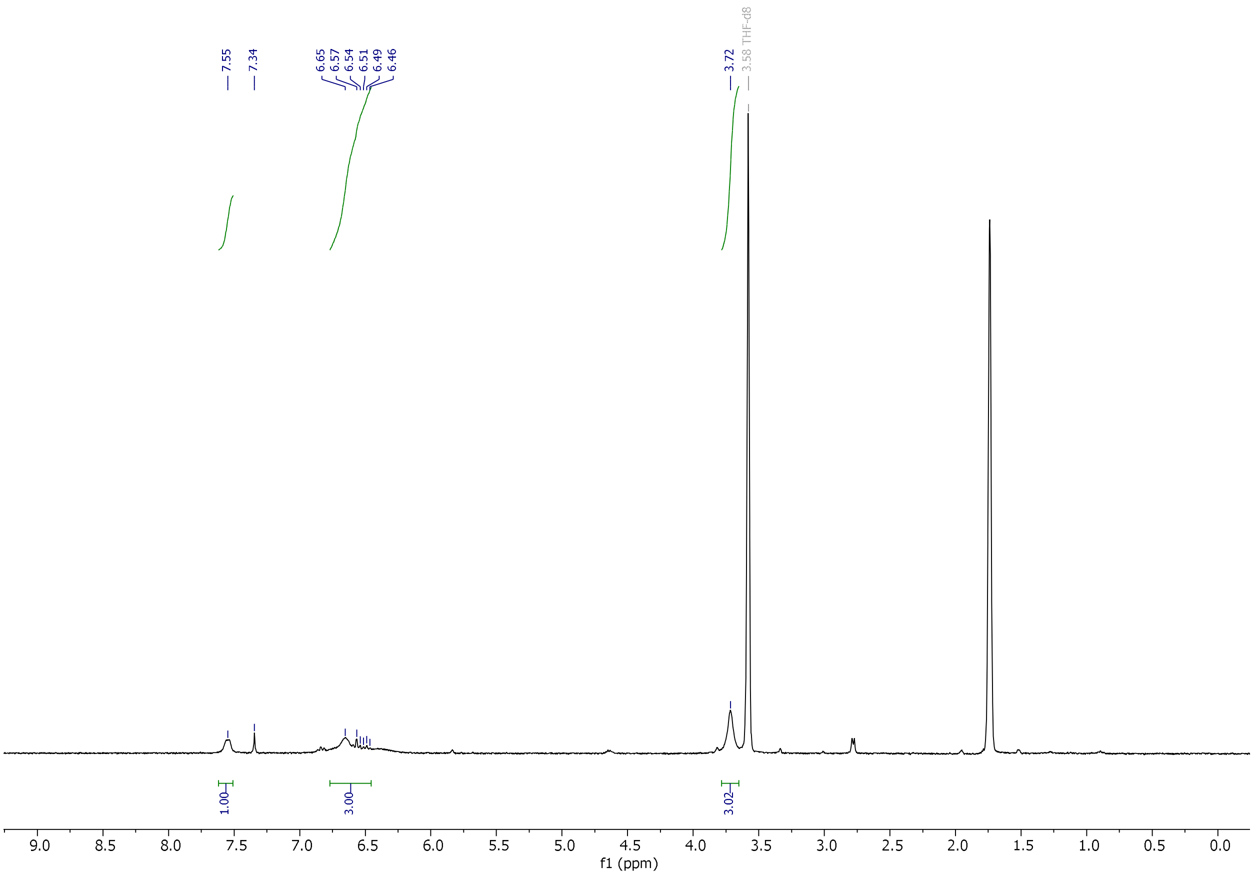

Figure S64. *^{1}H NMR spectrum (THF-d8, 300 MHz) of **2a** at 213 K. The doublet at 2.80 ppm is identified as the free ligand N–CH_3 proton signal.*

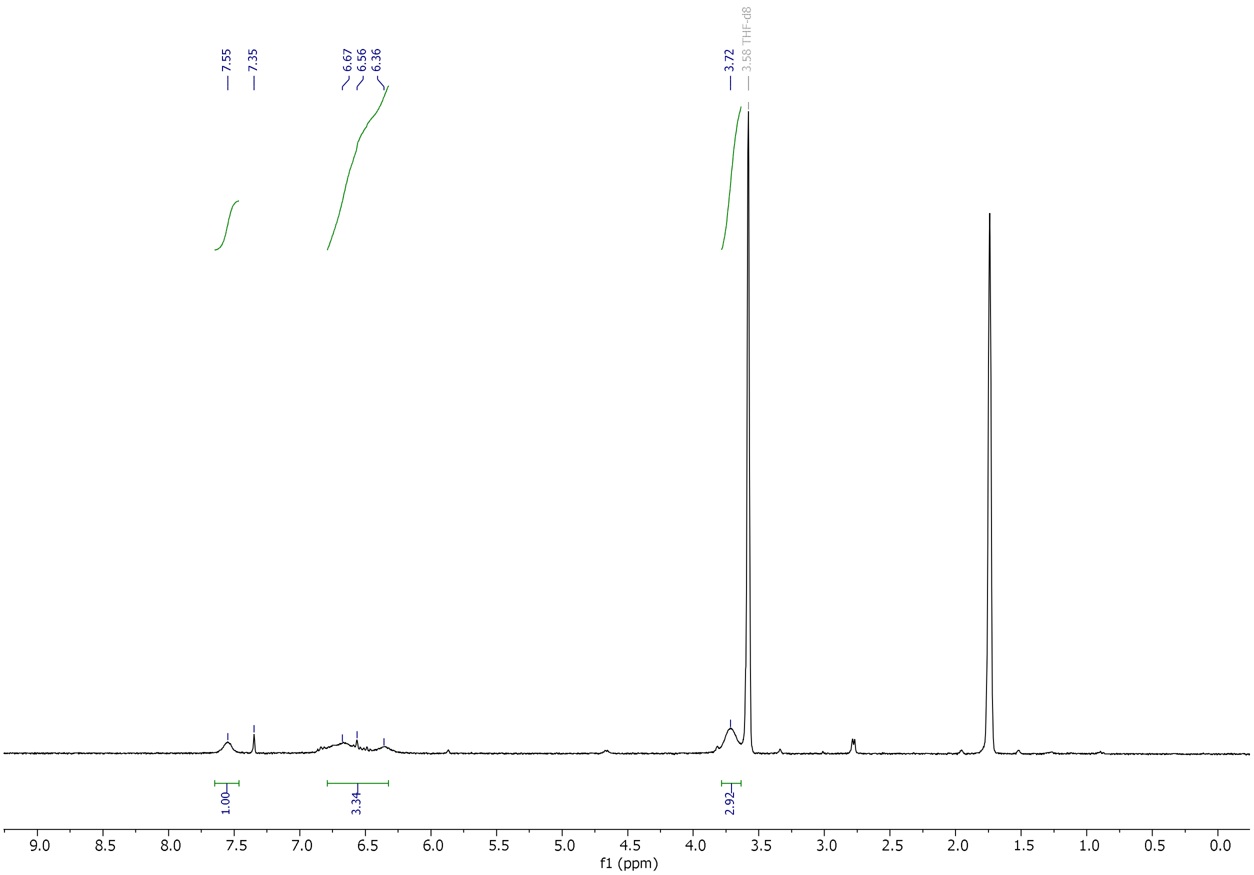

***Figure S65**. ^{1}H NMR spectrum (THF-d8, 300 MHz) of **2a** at 208 K. The doublet at 2.80 ppm is identified as the free ligand N–CH_3 proton signal.*

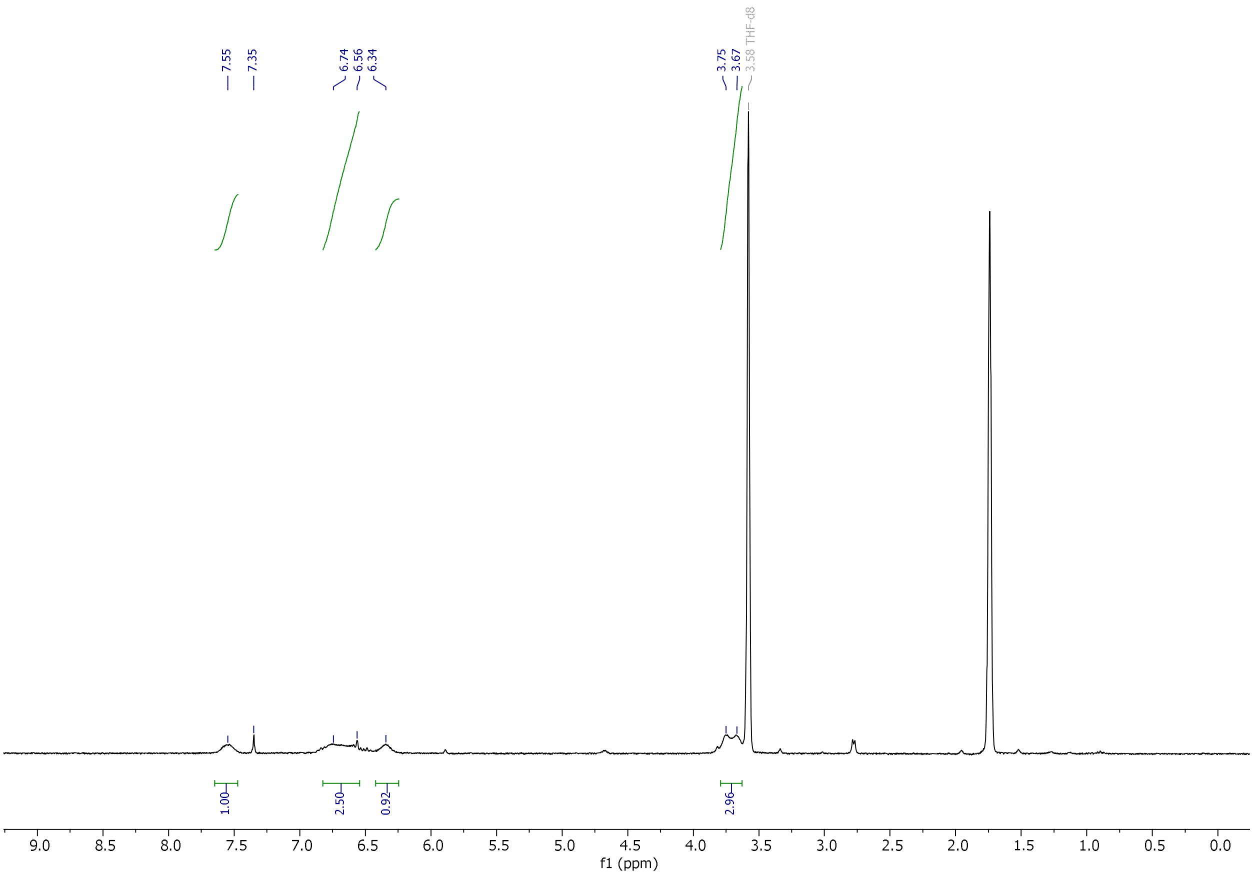

Figure S66. *^{1}H NMR spectrum (THF-d8, 300 MHz) of* ***2a*** *at 203 K. The doublet at 2.80 ppm is identified as the free ligand N–CH_3 proton signal.*

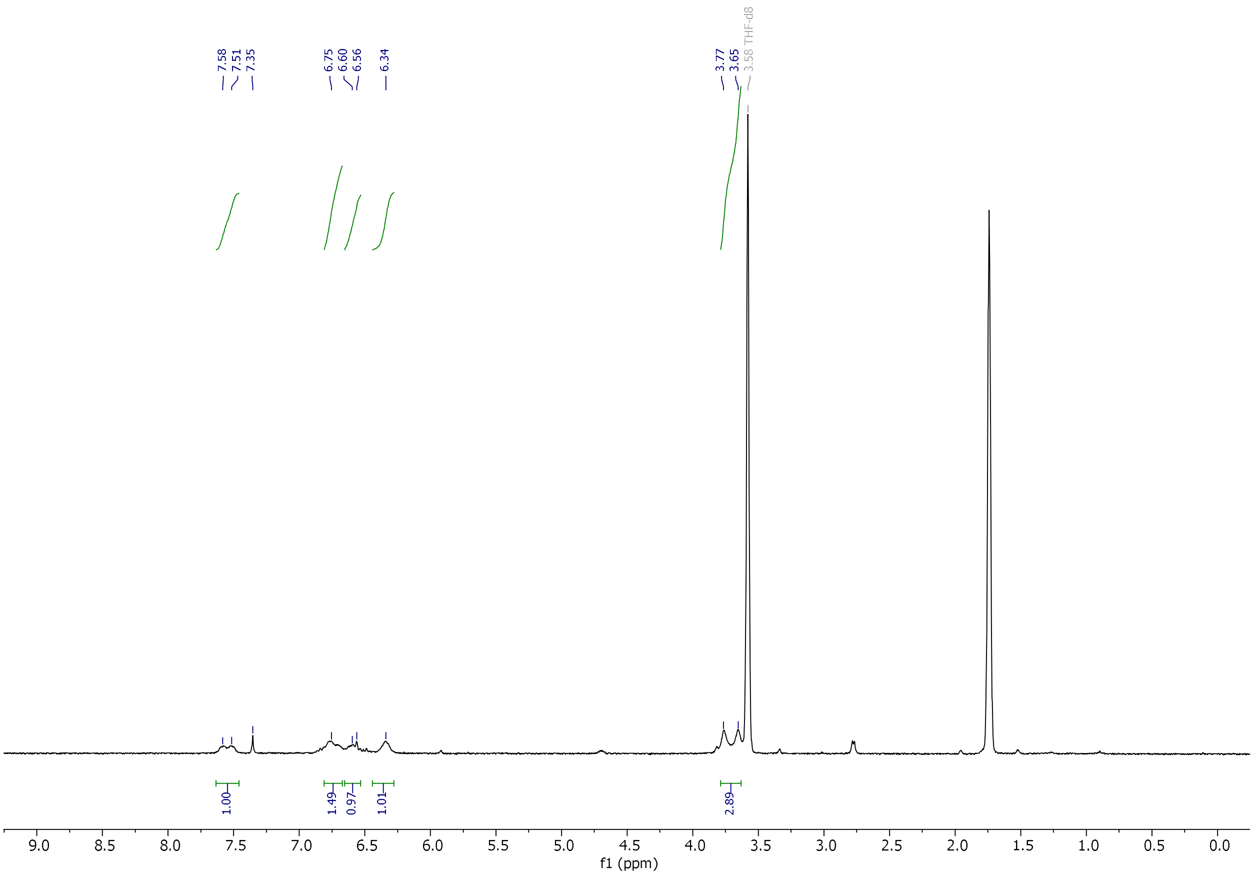

Figure S67. *^{1}H NMR spectrum (THF-d8, 300 MHz) of **2a** at 198 K. The doublet at 2.80 ppm is identified as the free ligand N–CH_3 proton signal.*

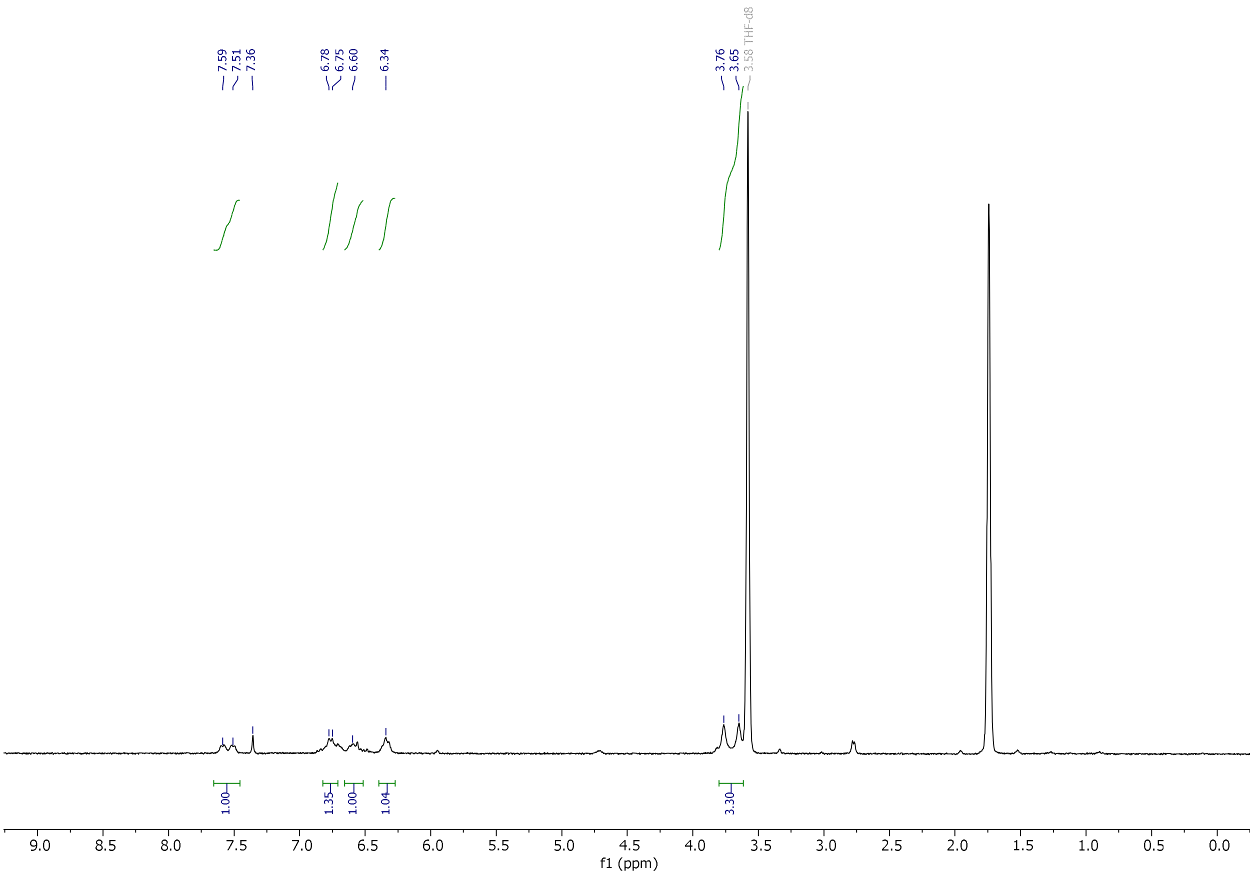

Figure S68. *^{1}H NMR spectrum (THF-d8, 300 MHz) of **2a** at 193 K. The doublet at 2.80 ppm is identified as the free ligand N–CH_3 proton signal.*

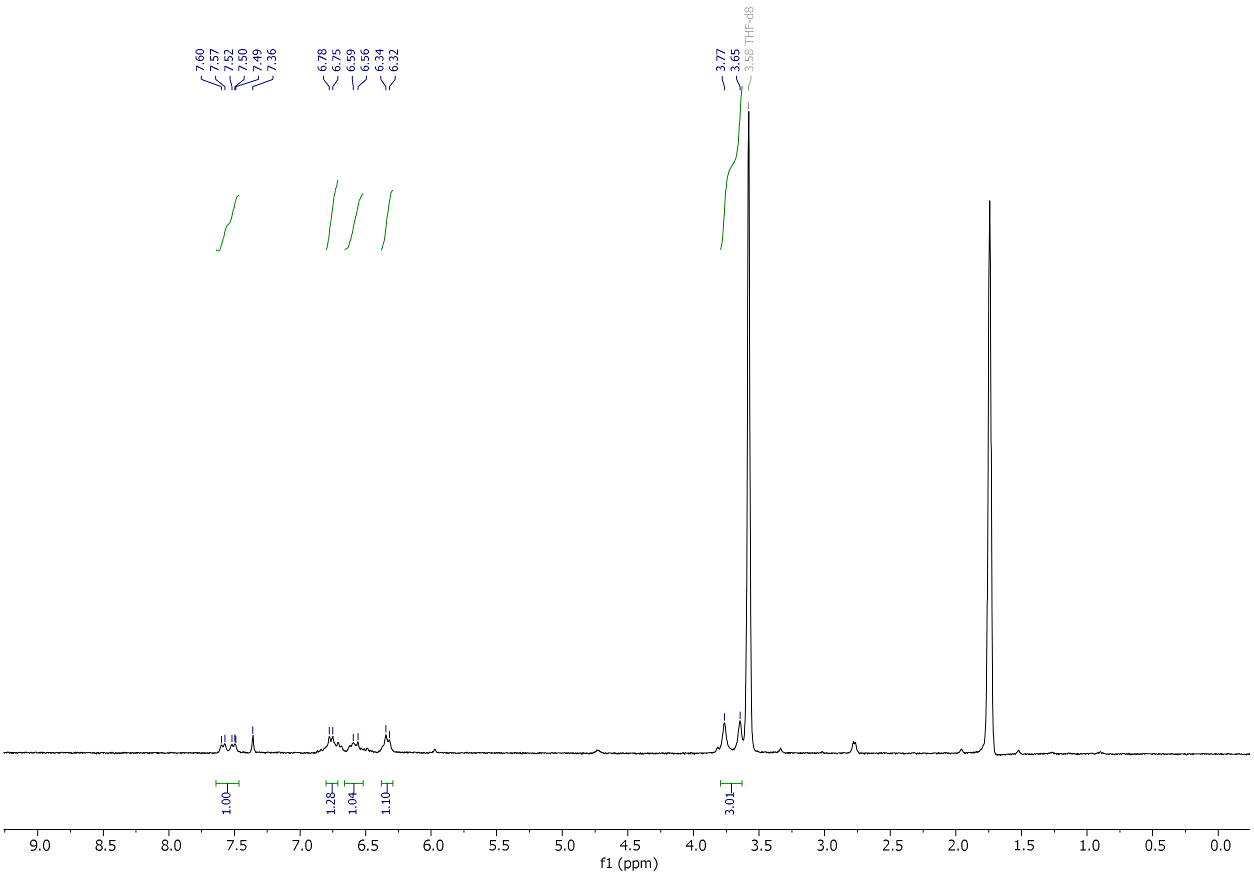

Figure S69. *^{1}H NMR spectrum (THF-d8, 300 MHz) of **2a** at 188 K. The doublet at 2.80 ppm is identified as the free ligand N–CH_3 proton signal.*

4.2.3 Individual VT-NMR Spectra for **4**

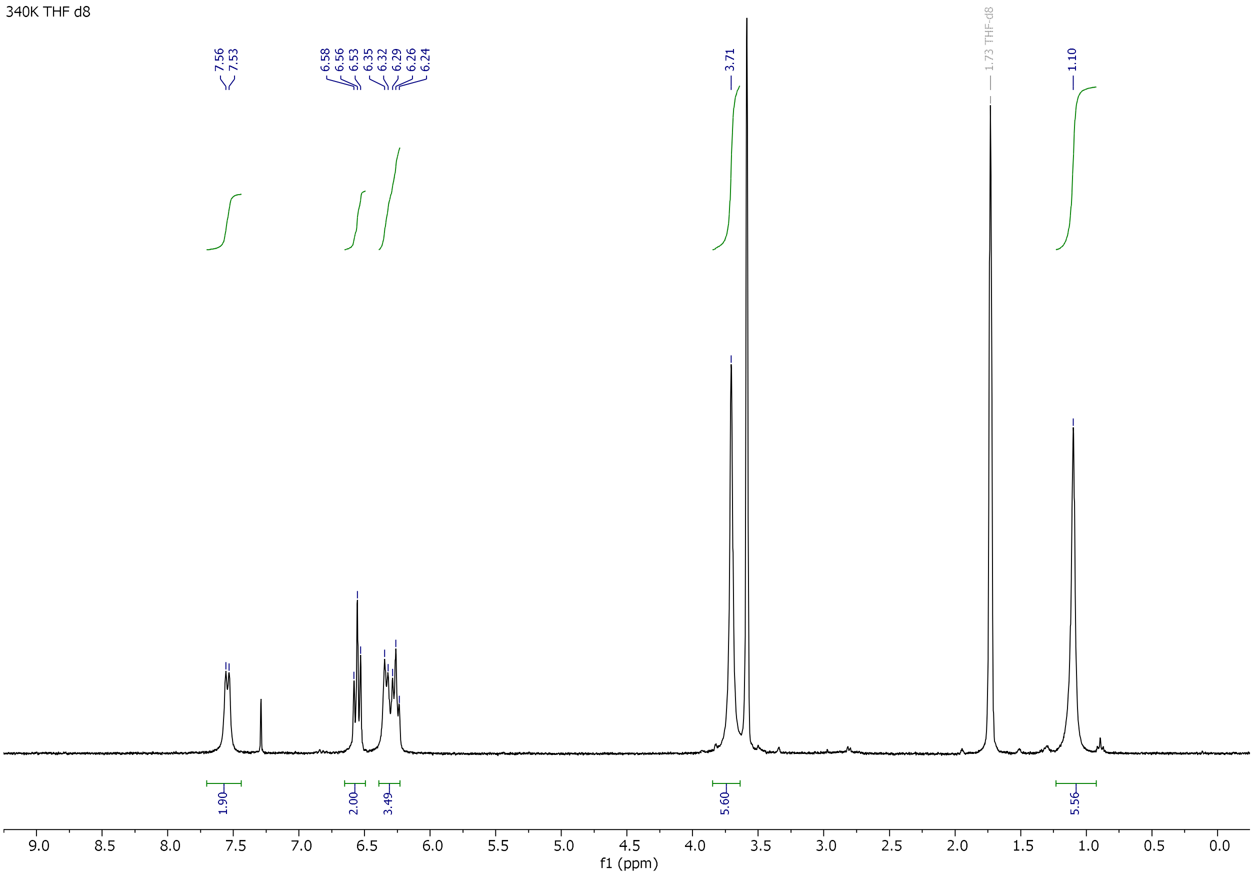

Figure S70. *^{1}H NMR spectrum (THF-d8, 300 MHz) of **4** at 340 K.*

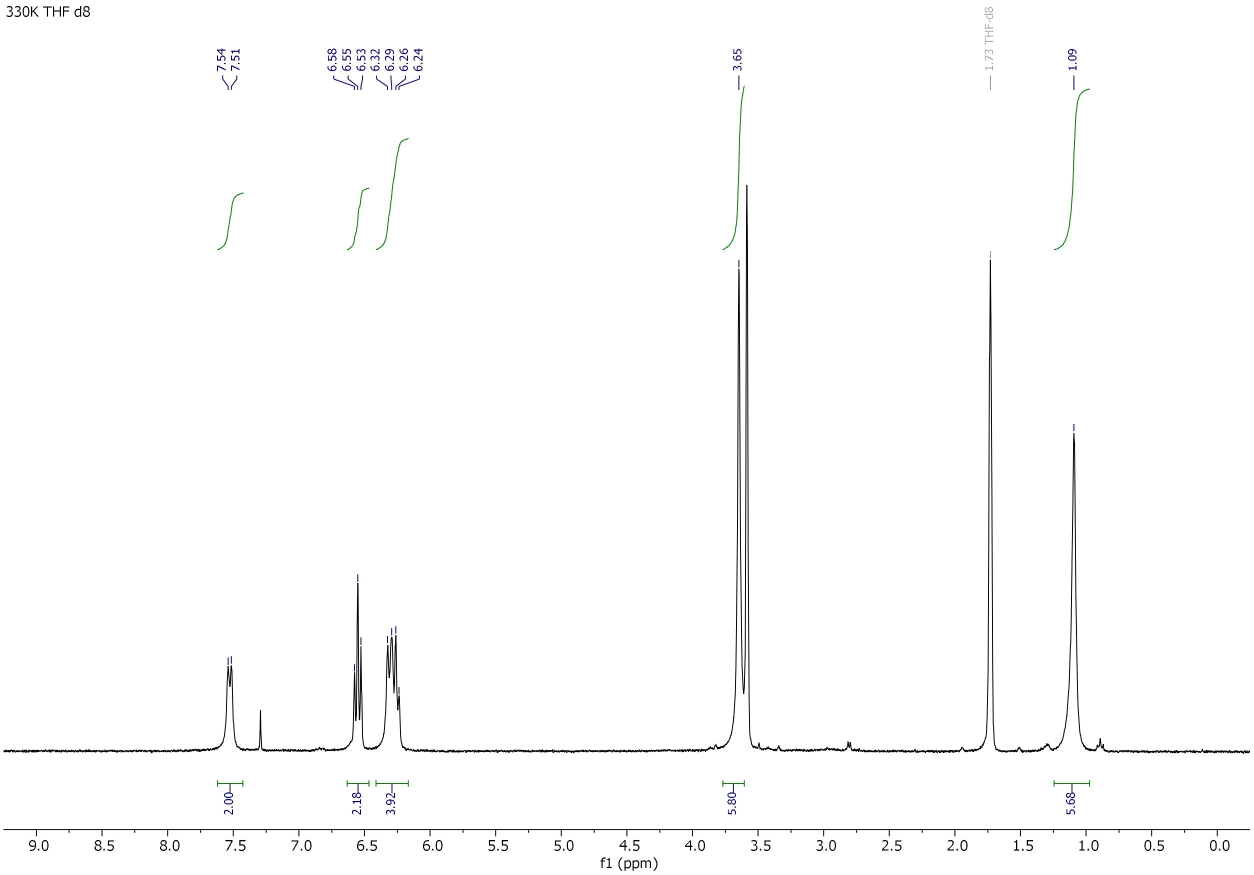

Figure S71. ^{1}H NMR spectrum (THF-d8, 300 MHz) of **4** at 330 K.

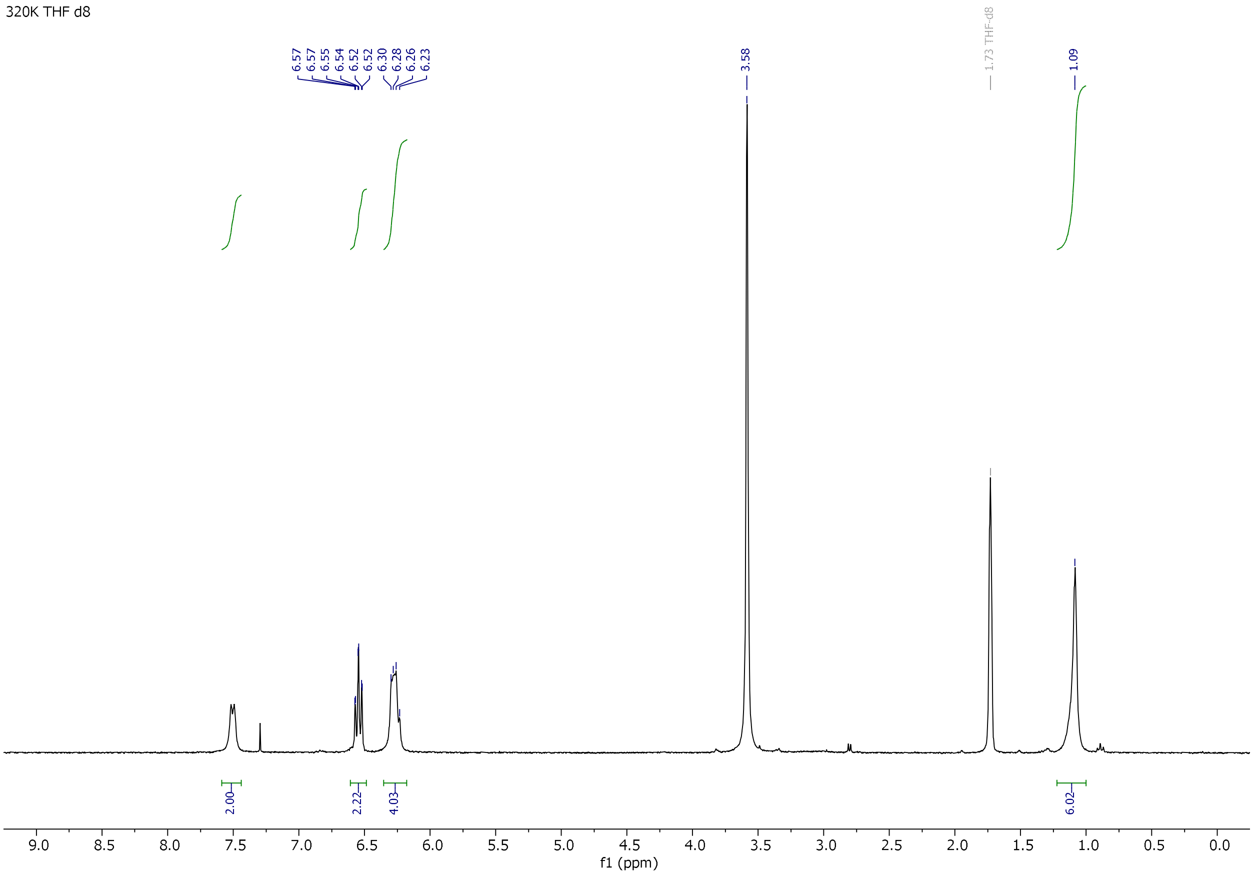

Figure S72. *^{1}H NMR spectrum (THF-d8, 300 MHz) of* ***4*** *at 320 K.*

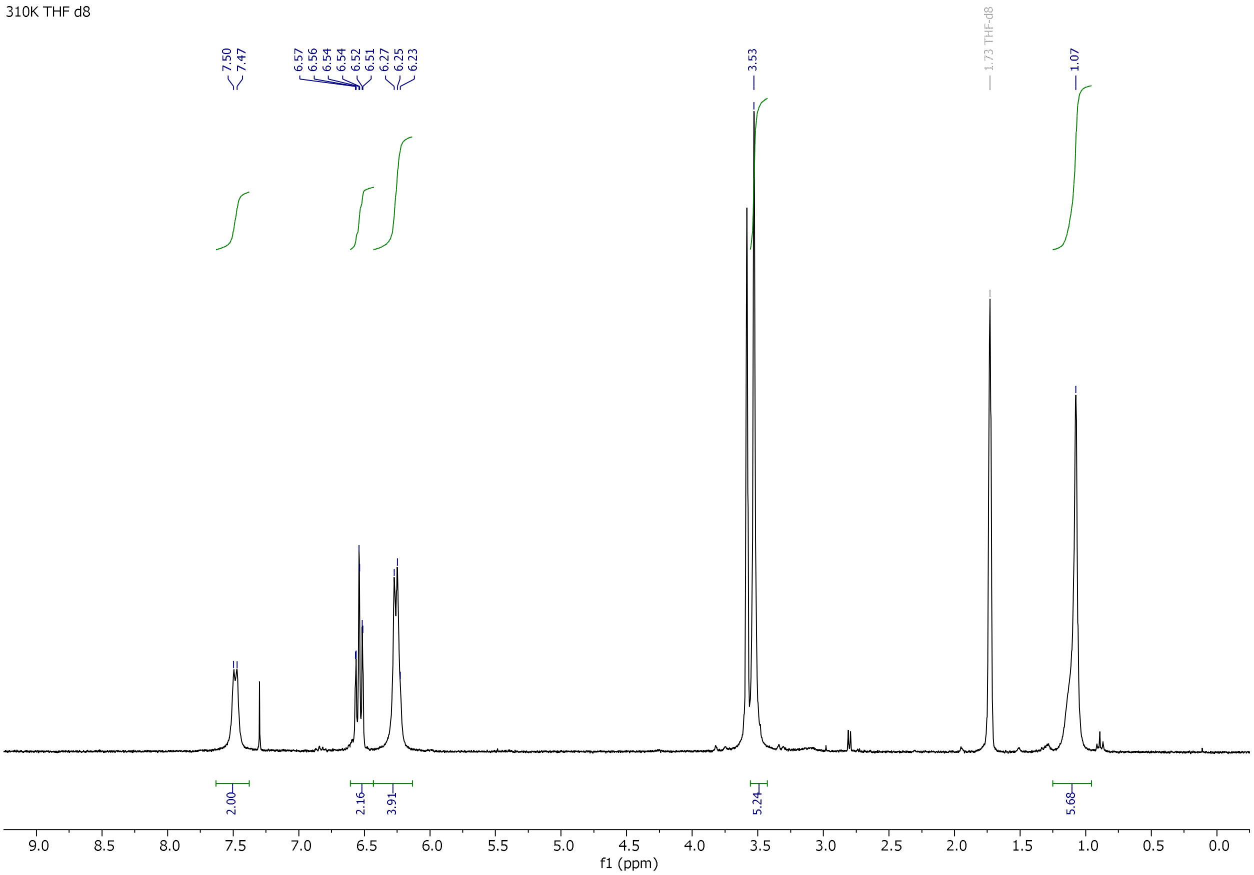

***Figure S73**. ^{1}H NMR spectrum (THF-d8, 300 MHz) of **4** at 310 K.*

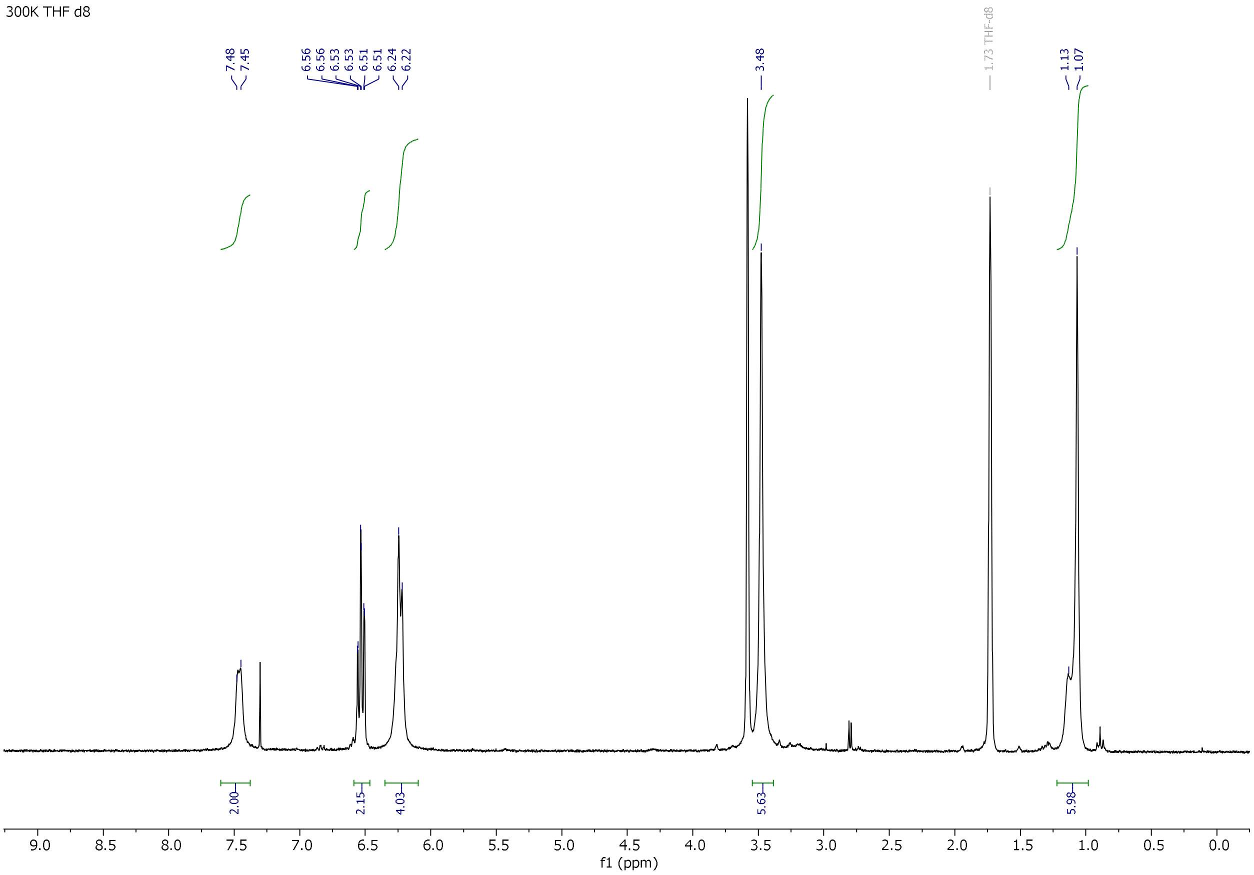

Figure S74. *^{1}H NMR spectrum (THF-d8, 300 MHz) of **4** at 300 K.*

4.2.4 Individual VT-NMR Spectra for **8**

340 K

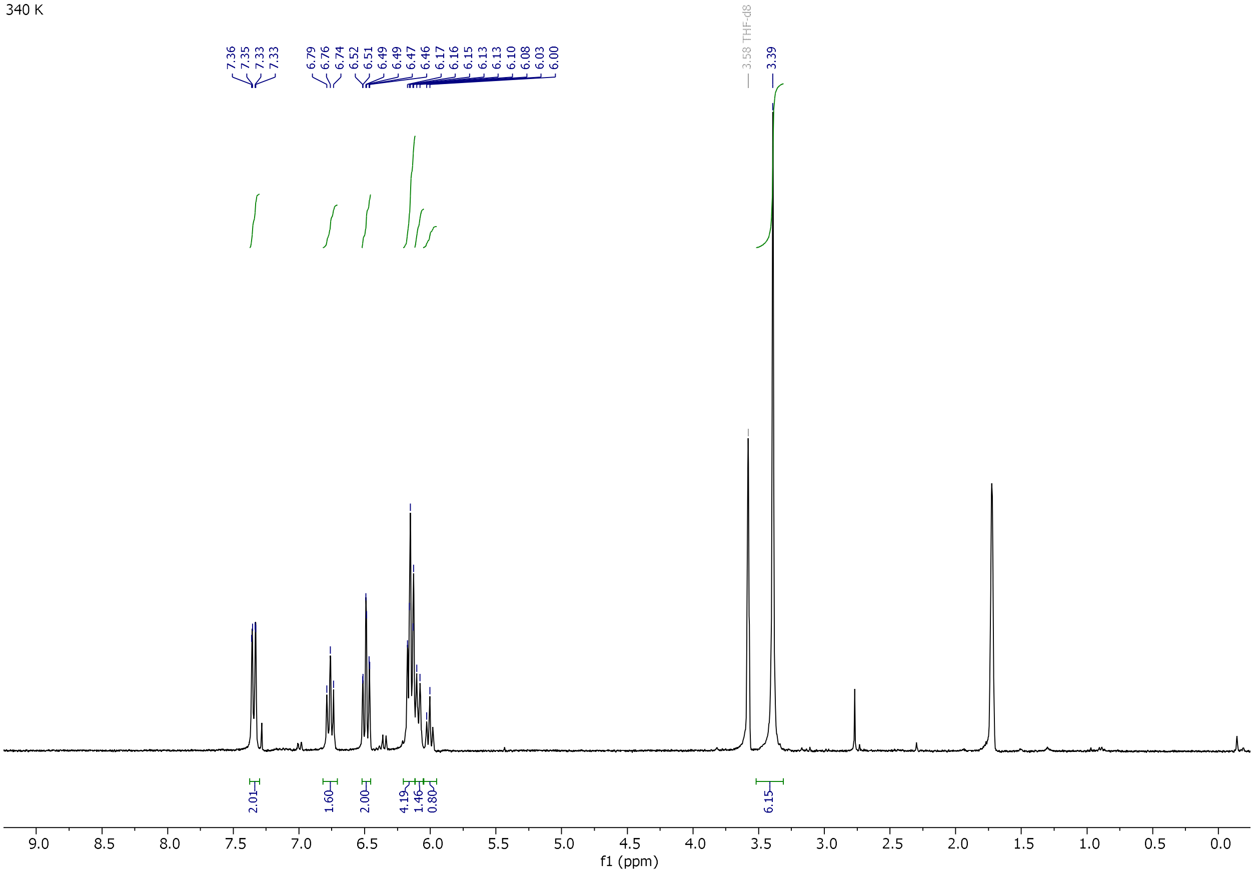

Figure S75. *¹H NMR spectrum (THF-d8, 300 MHz) of **8** at 340 K.*

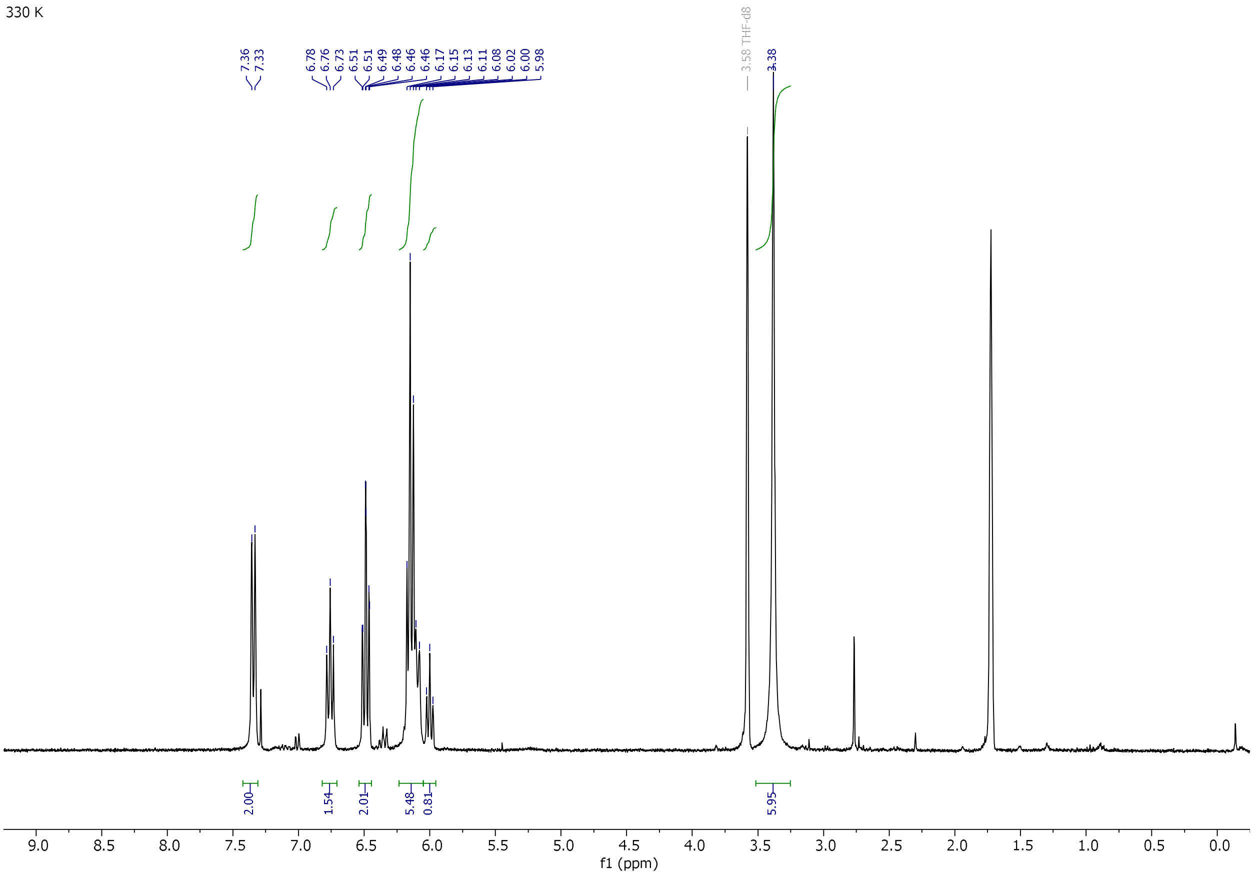

Figure S76. [1]*H NMR spectrum (THF-d8, 300 MHz) of* ***8*** *at 330 K.*

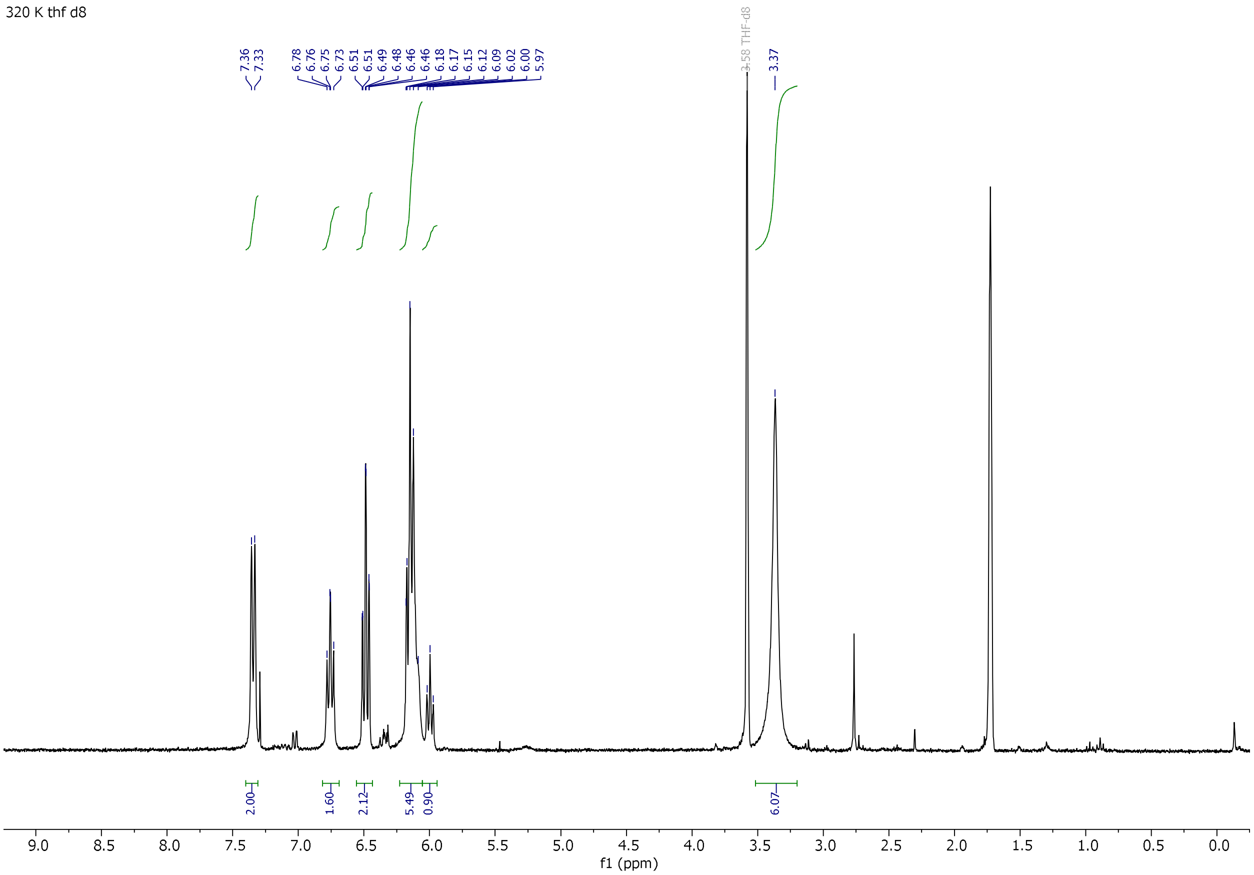

Figure S77. ^{1}H *NMR spectrum (THF-d8, 300 MHz) of **8** at 320 K.*

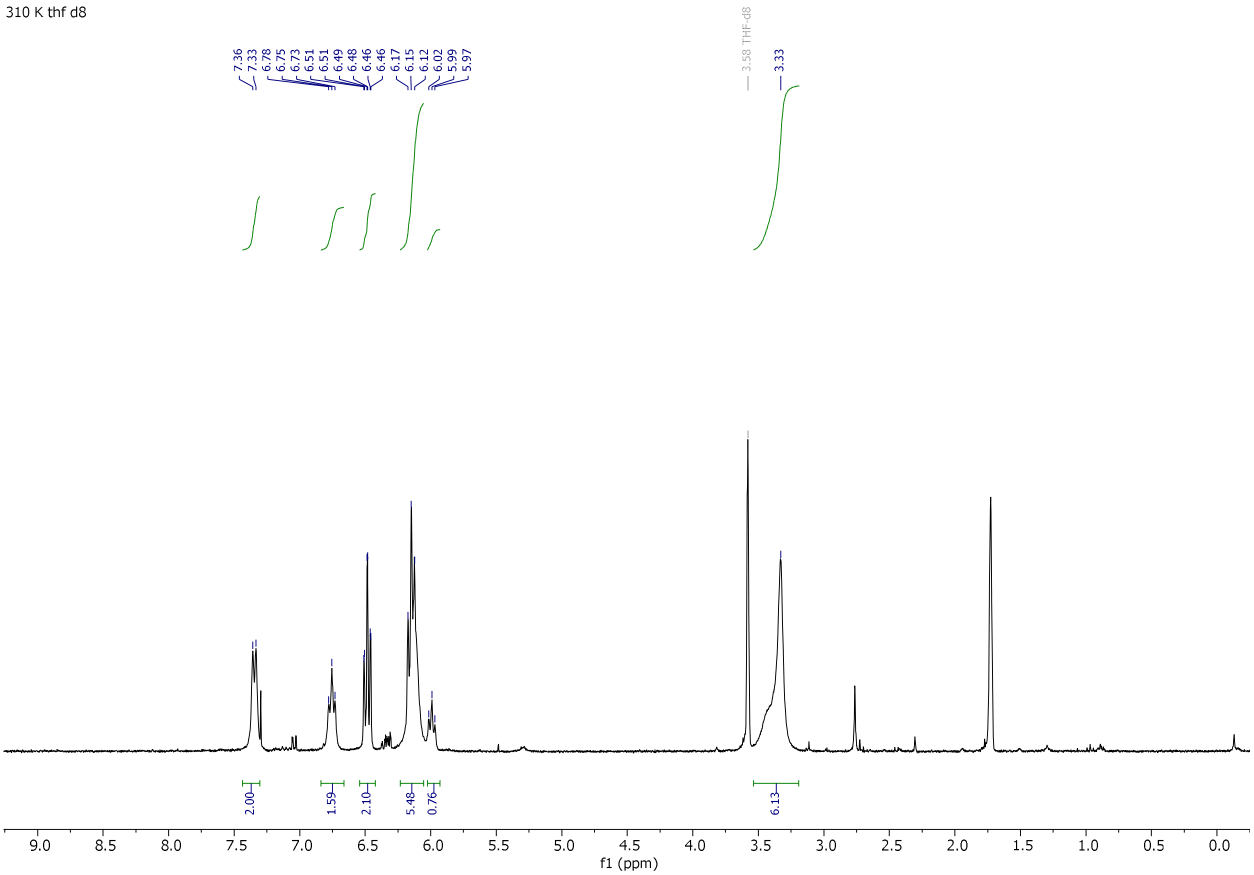

Figure S78. ^{1}H NMR spectrum (THF-d8, 300 MHz) of **8** at 310 K.

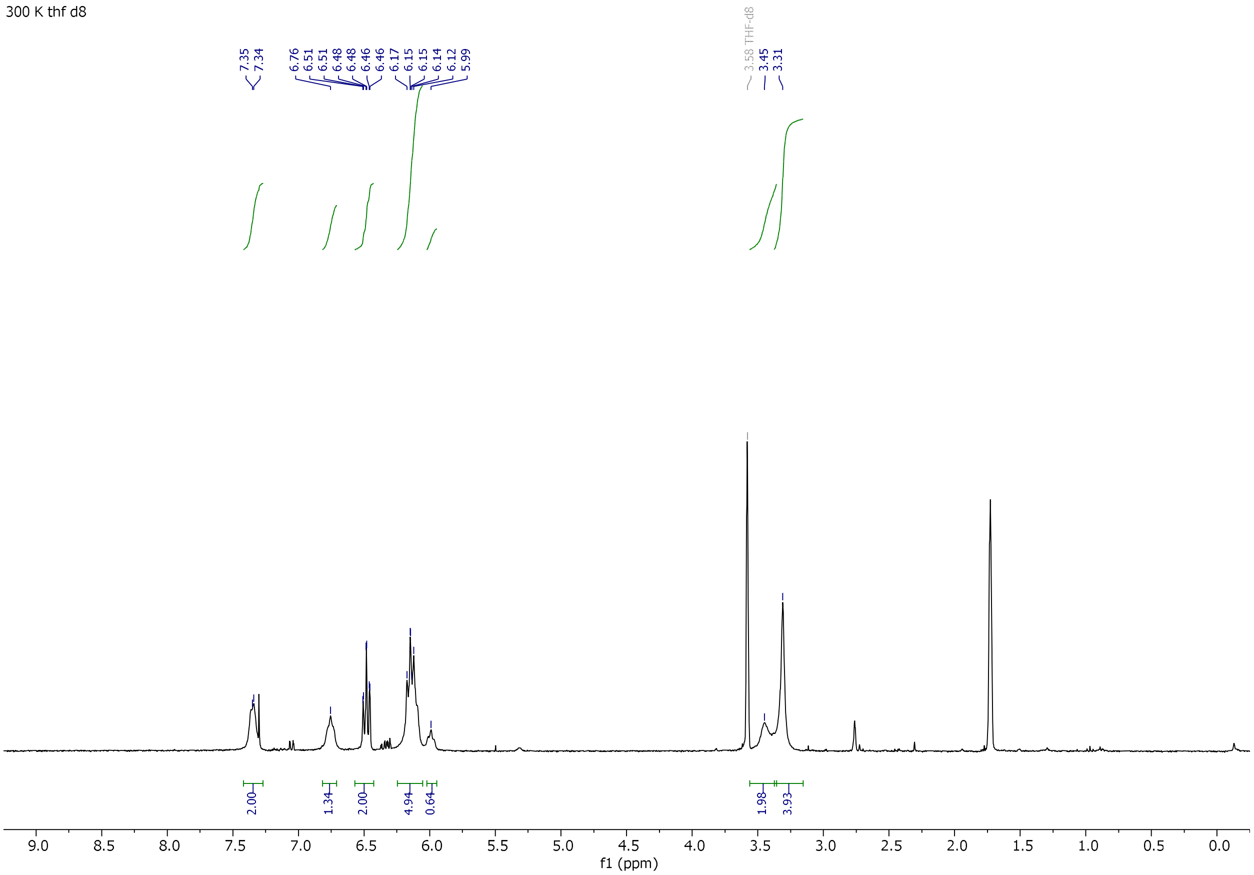

Figure S79. [1]*H NMR spectrum (THF-d8, 300 MHz) of* ***8*** *at 300 K.*

5 X-ray Crystallography Data

CCDC numbers: 2459250 (Compound **2a**), 2459252 (Compound 2b), 2459253 (Compound **3**), 2459254 (Compound **4**), 2459251 (Compound **5**), 2459249 (Compound **6**), 2495260 (**7**).

Table S4.

Compound reference	2a	2b	3	4
Chemical formula	$C_{28}H_{28}Bi_2N_6$•2(C_4H_8O)	$C_{30}H_{50}BiN_3Si_2$	$C_{40}H_{58}Bi_2N_6O_2P_2$	$C_{92}H_{124}Bi_4K_2N_{12}O_{11}$
Crystal colour	red	gold	orange	yellow
Formula Mass	1010.73	717.89	1134.82	2488.14
a/Å	7.3162(3)	15.7960(5)	8.3079(3)	11.9318(10)
b/Å	20.2186(8)	7.8257(2)	19.0371(7)	14.6398(13)
c/Å	11.3147(5)	25.3258(7)	13.0919(4)	14.8560(12)
α/°	90	90	90	77.535(3)
β/°	97.693(2)	96.0310(10)	103.3770(10)	74.628(3)
γ/°	90	90	90	74.494(3)
Unit cell volume/Å^3	1658.64(12)	3113.31(15)	2014.42(12)	2381.6(4)
Temperature/K	150.00	150.00	150.00	150.00
Space group	*P*121/*c*1	*P*21/*c*	*P*121/*c*1	$P\overline{1}$
Z	2	4	2	1
No. of reflections measured	83356	136828	51070	85925
No. of independent reflections	6329	11910	3561	9746
R_{int}	0.0254	0.0435	0.0450	0.0888
Final R_1 values ($I > 2\sigma(I)$)	0.0244	0.0171	0.0282	0.0565
Final $wR(F^2)$ values ($I > 2\sigma(I)$)	0.0634	0.0341	0.0639	0.1423
Final R_1 values (all data)	0.0255	0.0243	0.0383	0.0943
Final $wR(F^2)$ values (all data)	0.0642	0.0366	0.0728	0.1736
Goodness of fit on F^2	1.121	1.031	1.204	1.117

Table S3 (cont.)

Compound reference	5	6	7
Chemical formula	$C_{136}H_{236}Bi_4K_4N_{12}O_4Si_8$	$C_{28}H_{28}N_6W$•C_4H_8O	$C_{23}H_{33}BiKN_3O_3$
Crystal colour	yellow	black	Yellow
Formula Mass	3320.39	704.52	647.60
a/Å	31.604(5)	7.5762(8)	13.367(5)
b/Å	14.541(2)	7.9003(7)	9.742(4)
c/Å	33.340(10)	11.9231(12)	18.652(6)
α/°	90	95.755(3)	90
β/°	90	101.762(4)	98.394(4)
γ/°	90	92.308(4)	90
Unit cell volume/Å^3	15322(6)	693.79(12)	2402.87(15)
Temperature/K	150.00	150.00	150.00
Space group	*Pca*21	$P\overline{1}$	*P*21/*n*
Z	4	1	4
No. of reflections measured	128220	3532	54074
No. of independent reflections	29197	3532	4563
R_{int}	0.0517	?	0.0750
Final R_1 values ($I > 2\sigma(I)$)	0.0303	0.0338	0.0337
Final $wR(F^2)$ values ($I > 2\sigma(I)$)	0.0594	0.0793	0.0788
Final R_1 values (all data)	0.0370	0.0521	0.0482
Final $wR(F^2)$ values (all data)	0.0620	0.0813	0.0922
Goodness of fit on F^2	1.020	1.091	1.135

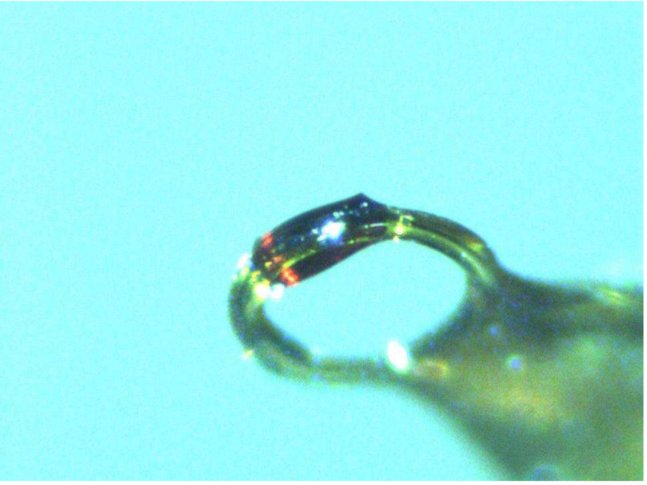

Figure S80. *Photograph of a crystal of* ***2a****.*

5.1 Crystal Structures

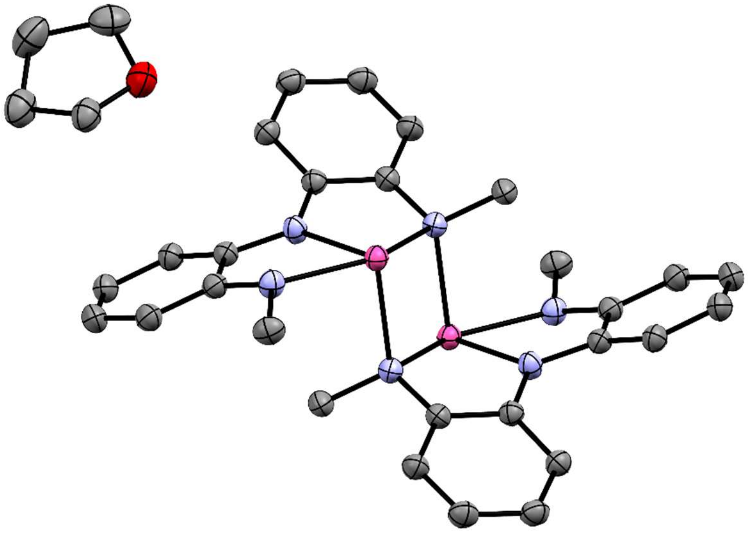

Figure S81. *Solid-state structure of* ***2a*** *in dimeric form with spectator tetrahydrofuran. Hydrogen atoms omitted for clarity. Thermal ellipsoids drawn at 50% probability. Pink: bismuth, grey: carbon, light blue: nitrogen, red: oxygen.*

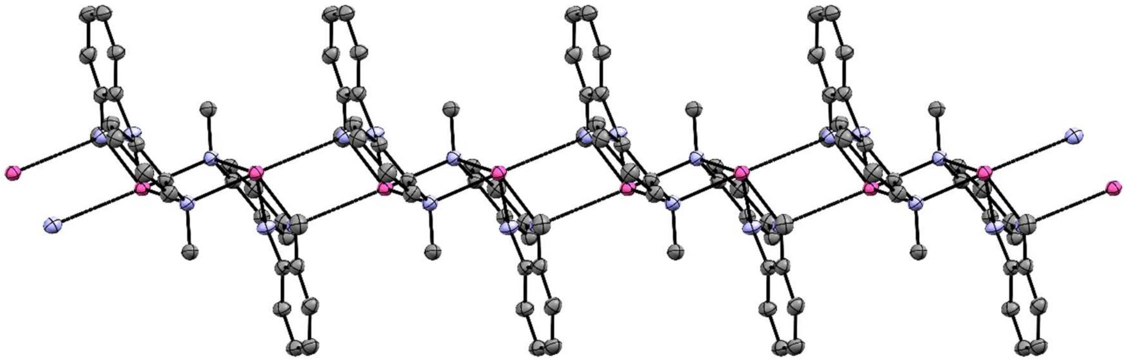

Figure S82. *Extended solid-state structure of* ***2a*** *along the c-axis. Hydrogen atoms and THF solvent molecules omitted for clarity. Thermal ellipsoids drawn at 50% probability. Pink: bismuth, grey: carbon, light blue: nitrogen.*

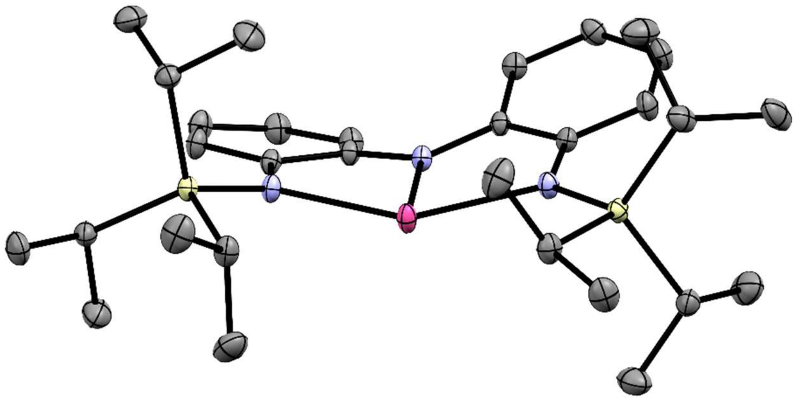

Figure S83. *Solid-state structure of* ***2b*** *in dimeric form with spectator tetrahydrofuran. Hydrogen atoms omitted for clarity. Thermal ellipsoids drawn at 50% probability. Pink: bismuth, grey: carbon, light blue: nitrogen, beige: silicon.*

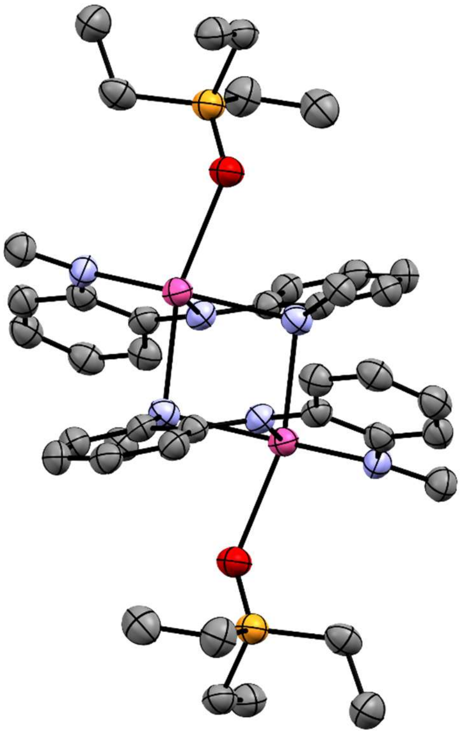

Figure S84. *Solid-state structure of* ***3*** *Hydrogen atoms omitted for clarity. Thermal ellipsoids drawn at 50% probability. Pink: bismuth, grey: carbon, light blue: nitrogen, orange: phosphorus, red: oxygen.*

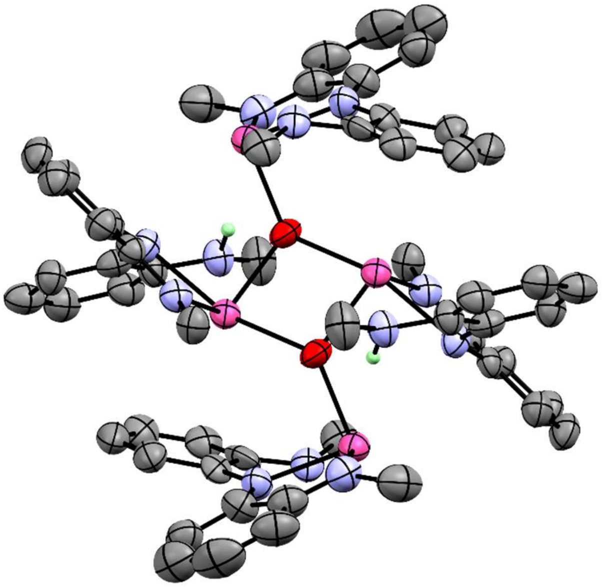

Figure S85. *Solid-state structure of* ***4*** *dianion with protonated amine hydrogen atoms coloured in green. All other hydrogen atoms, potassium cations and coordinated THF molecules are omitted for visual clarity. Thermal ellipsoids drawn at 50% probability. Pink: bismuth, grey: carbon, light blue: nitrogen, red: oxygen, green: hydrogen.*

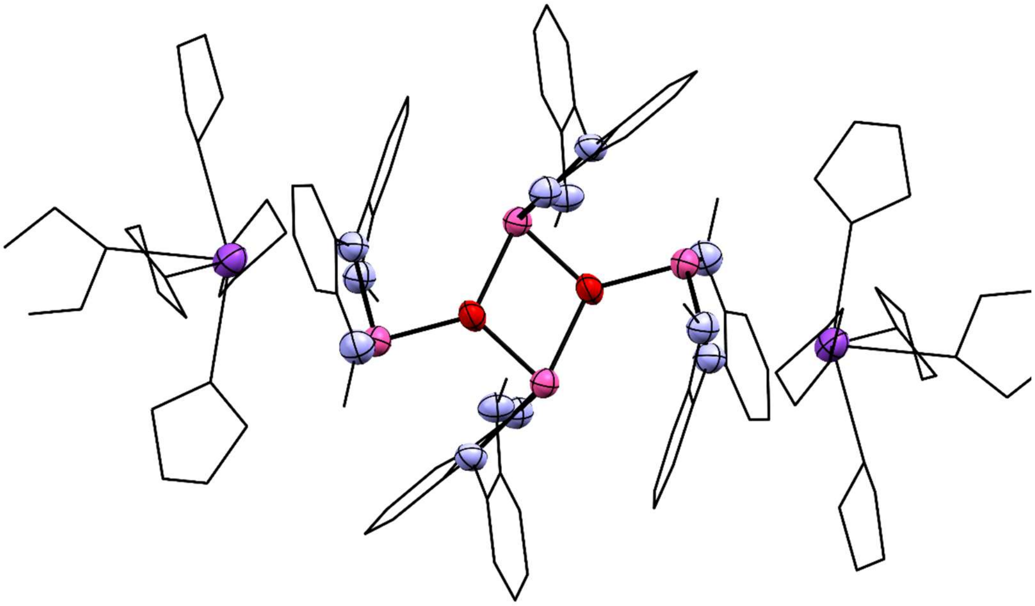

Figure S86. *Solid-state structure of **4** with potassium and coordinated THF molecules. Hydrogen atoms omitted, carbon backbone and THF molecules rendered in wireframe for visual clarity. Thermal ellipsoids drawn at 50% probability. Pink: bismuth, light blue: nitrogen, red: oxygen.*

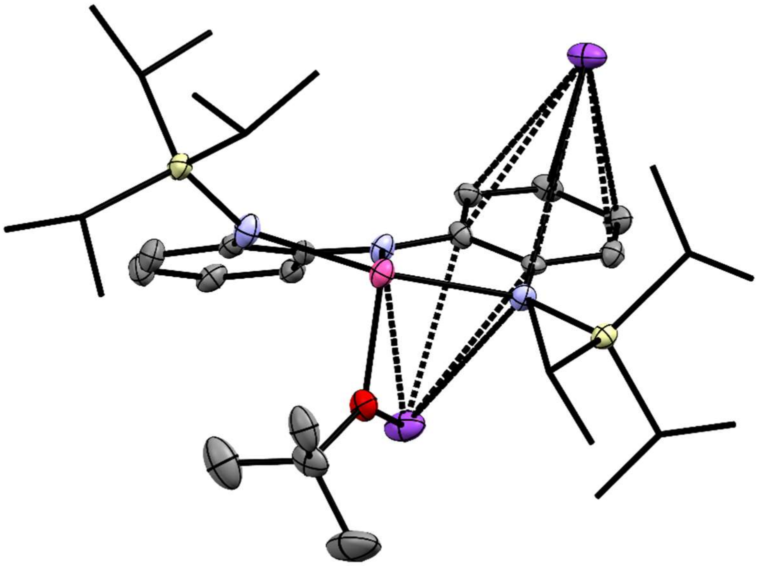

Figure S87. *Solid-state structure of **5** monomer unit with bridging potassium cations. Hydrogen atoms are omitted and isopropyl groups are displayed as wireframes for visual clarity. Thermal ellipsoids drawn at 50% probability. Pink: bismuth, grey: carbon, light blue: nitrogen, red: oxygen, purple: potassium, beige: silicon.*

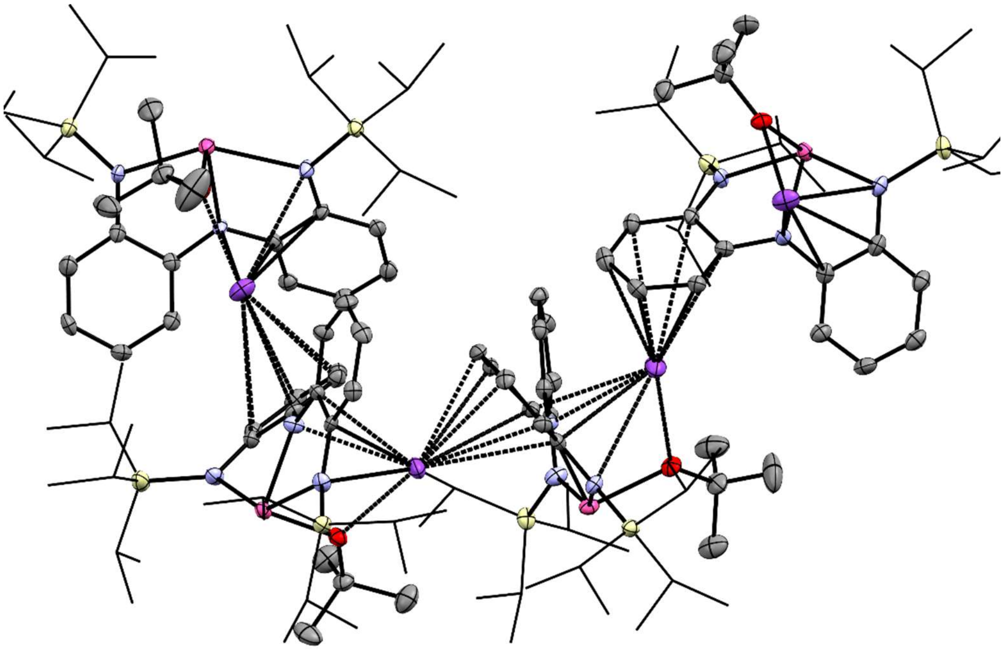

***Figure S88**. Solid-state structure of **5** as tetrameric structure. Hydrogen atoms are omitted and isopropyl groups are displayed as wireframes for visual clarity. Thermal ellipsoids drawn at 50% probability. Pink: bismuth, grey: carbon, light blue: nitrogen, red: oxygen, purple: potassium, beige: silicon.*

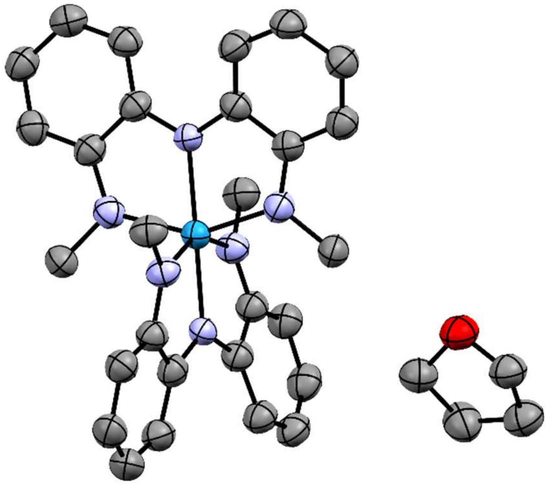

***Figure S89**. Solid-state structure of **6** in with spectator tetrahydrofuran. Hydrogen atoms omitted for clarity. Thermal ellipsoids drawn at 50% probability. Blue: tungsten, grey: carbon, light blue: nitrogen, red: oxygen.*

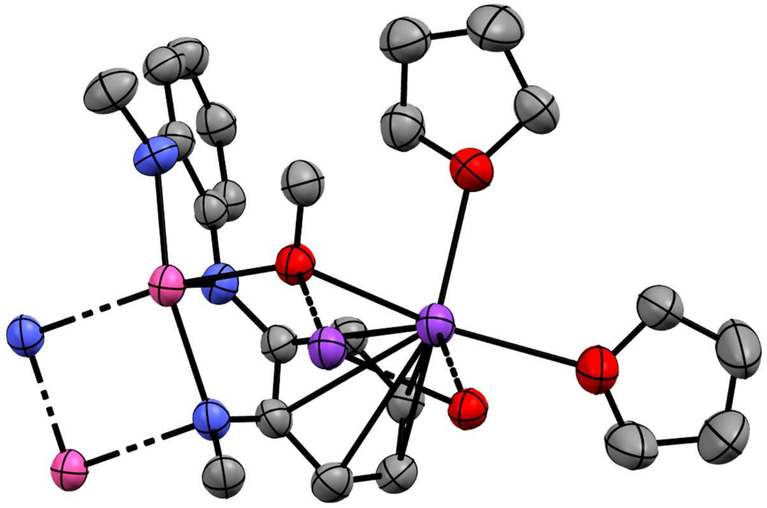

Figure S90. *Solid-state structure of **7** showing coordination of potassium cation to two tetrahydrofuran molecules and the aryl ring of **7**. Hydrogen atoms omitted for clarity. Thermal ellipsoids drawn at 50% probability. Pink: bismuth, grey: carbon, light blue: nitrogen, red: oxygen, purple: potassium.*

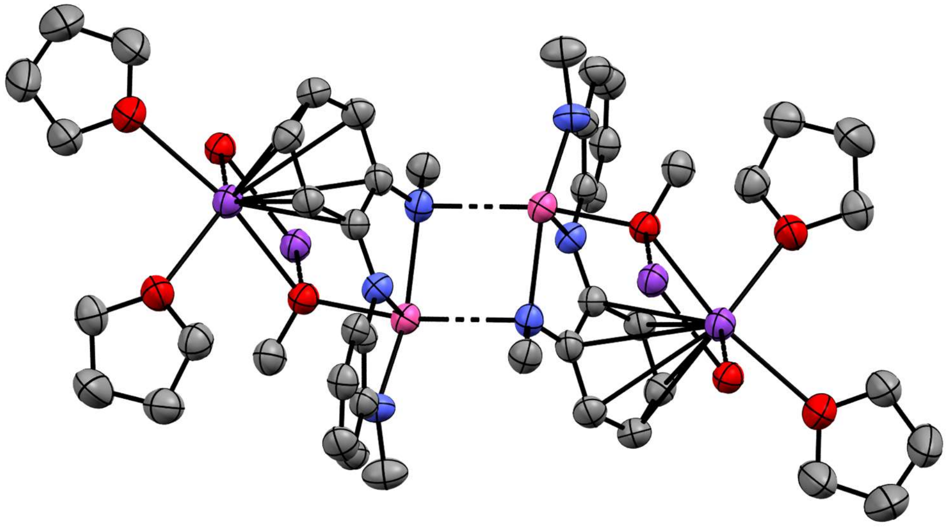

Figure S91. *Extended solid-state structure of **7** showing comparatively loose monomer-monomer interactions (compare Bi–N3' distance of **7** (2.716(5) Å) with **2a** (2.329(2) Å)). Hydrogen atoms omitted for clarity. Thermal ellipsoids drawn at 50% probability. Pink: bismuth, grey: carbon, light blue: nitrogen, red: oxygen, purple: potassium.*

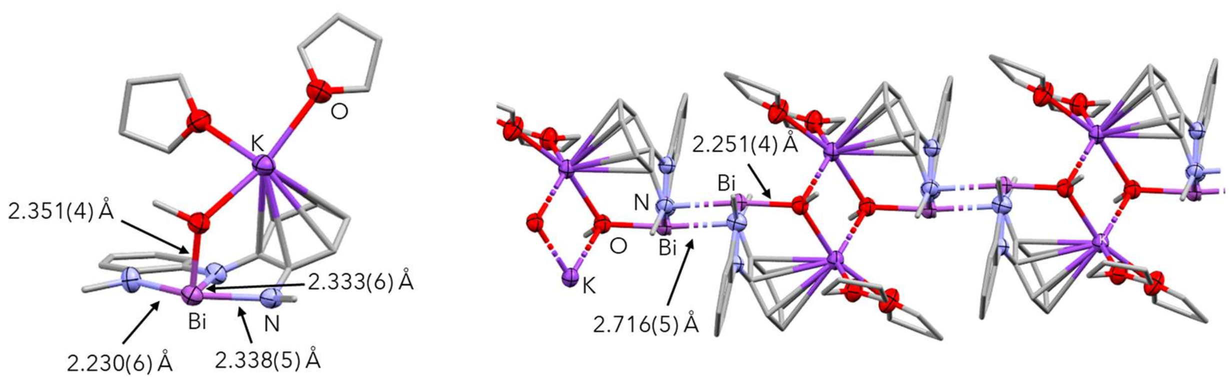

Figure S92. *Views of the molecular structure of **7**.*

6 Computational Methods

6.1 Determination of Percent Buried Volume

The percentage of buried volume, $\%V_{bur}$, quantifies the volume occupied by a ligand within the first coordination sphere around a metal centre.[14,13] In a $\%V_{bur}$ calculation, a sphere of radius R is drawn around the metal centre and partitioned into voxels, representing the coordination sphere of the metal, V_{sphere}. For each voxel, the distance from the centre of the voxel to all atoms of the ligand is checked. If a ligand atom is within a distance less than the van der Waals radius of the atom from the voxel centre, the voxel is classified as buried volume (V_{bur}). If no atoms are found within the van der Waals radius, then the voxel is classified as free volume V_{free}. Thus, $\%V_{bur}$ results from the quotient of V_{bur}/V_{sphere} multiplied by 100%. $\%V_{bur}$ calculations were performed using *SambVca 2.1* based on DFT optimized geometry coordinates.[14,16,17] R was set to R = 3.5 Å and bond radii were scaled by 1.17 for all $\%V_{bur}$ calculations. All calculations were performed with ORCA 6.0.0.[18] Geometry optimizations and dihedral scans were done using the r2-SCAN-3c composite method.[19]

6.2 Percent Buried Volume Data

6.2.1 %V_{bur} values

***Table S5**. %V_{bur} data*

Compound	$\%V_{bur}$
**2a**	43.0%
**2b**	62.1%
**B**	59.9%
**D**	54.9%

6.2.2 Steric Maps

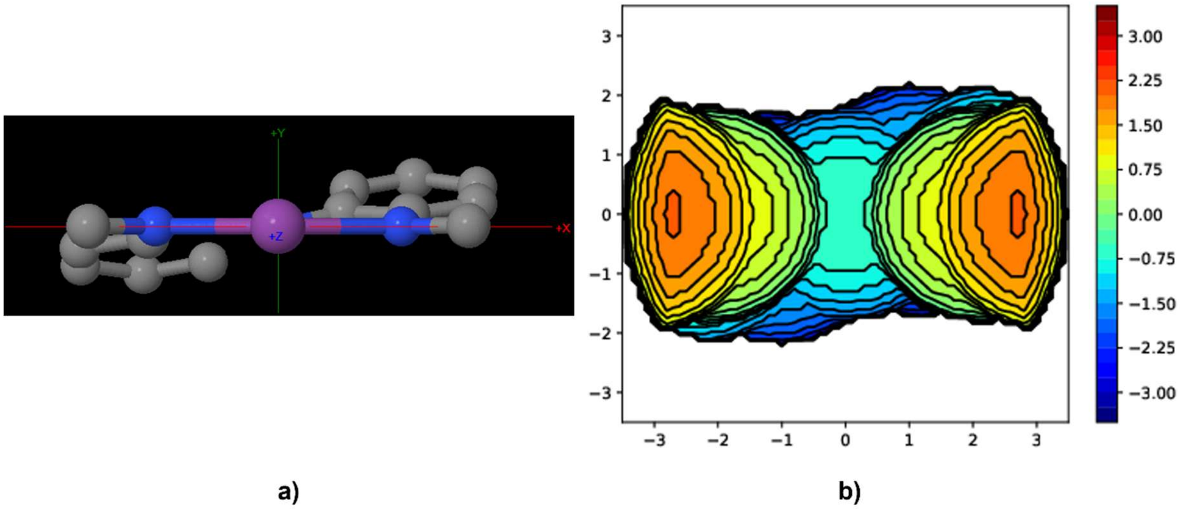

Figure S93. *Orientation of **2a** along the z-axis (a). Topographic steric map calculated for **2a**, viewed along the z-axis (b). Steric map axes refer to the x- and y-axes. Purple: bismuth, blue: nitrogen, grey: carbon. Protons have been omitted from the calculation. %V_{bur} = 43.0%.*

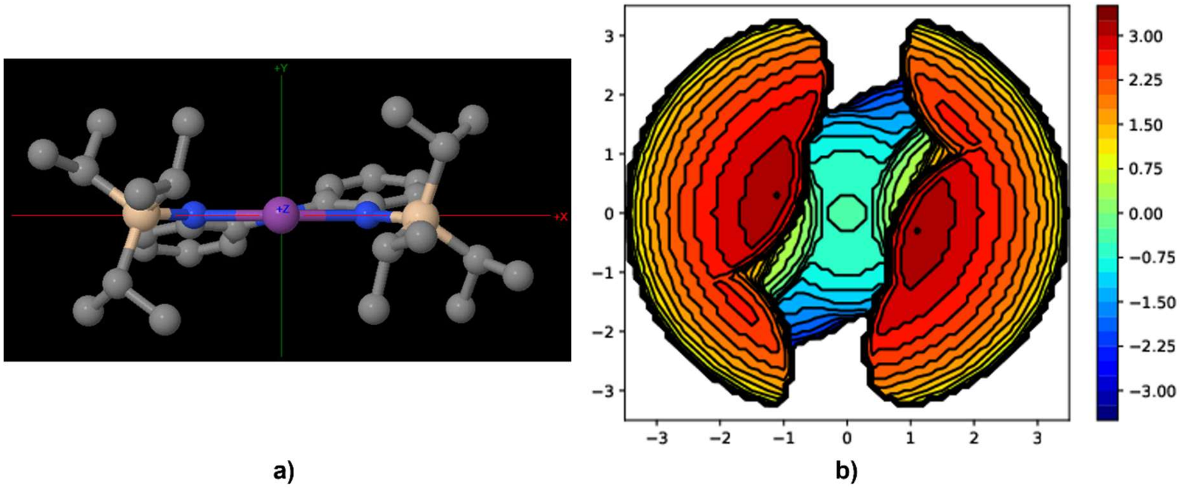

Figure S94. *Orientation of **2b** along the z-axis (a). Topographic steric map calculated for **2a**, viewed along the z-axis (b). Steric map axes refer to the x- and y-axes. Purple: bismuth, blue: nitrogen, grey: carbon, beige: silicon. Protons have been omitted from the calculation. %V_{bur} = 62.1%.*

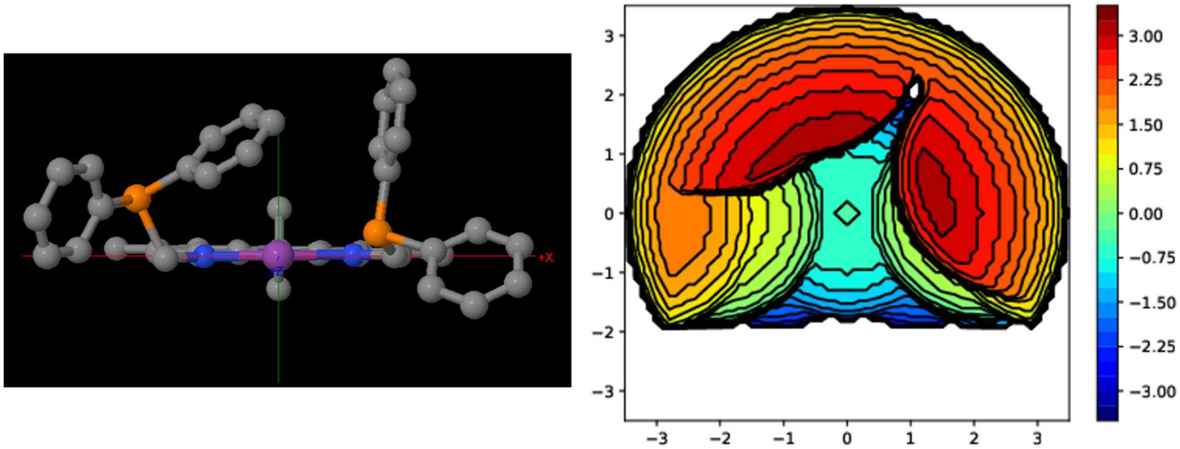

a) b)

Figure S95. *Orientation of* ***B*** *along the z-axis (a). Topographic steric map calculated for* ***2a****, viewed along the z-axis (b). Steric map axes refer to the x- and y-axes. Purple: bismuth, blue: nitrogen, grey: carbon, orange: phosphorus. Protons have been omitted from the calculation. %V_{bur} = 59.9%.*

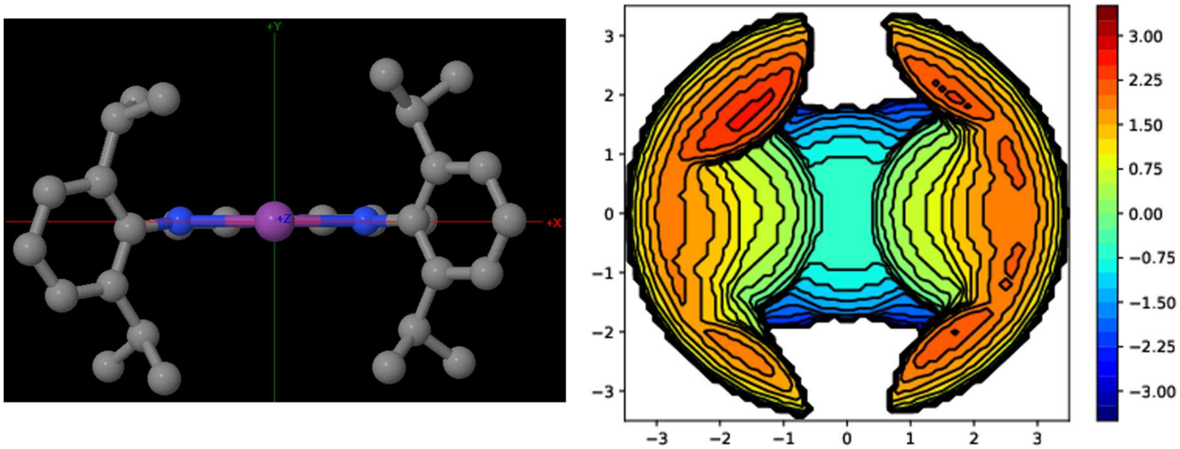

a) b)

Figure S96. *Orientation of* ***D*** *along the z-axis (a). Topographic steric map calculated for* ***2a****, viewed along the z-axis (b). Steric map axes refer to the x- and y-axes. Purple: bismuth, blue: nitrogen, grey: carbon. Protons Shave been omitted from the calculation. %V_{bur} = 54.9%.*

6.3 Calculated UV-Vis spectra and assignments

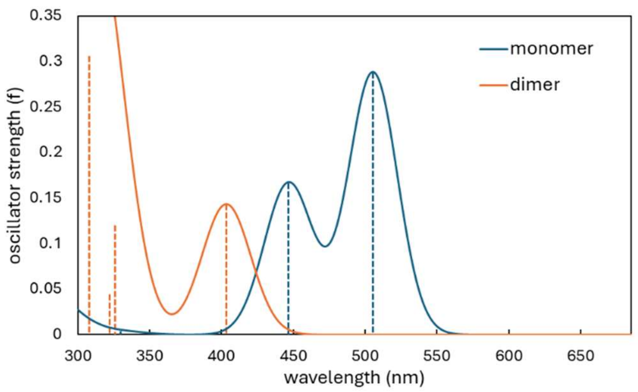

***Figure S97**. Calculated (ωb97xd/def2tzvp) UV-Vis spectra of **2a** and **2a'**.*

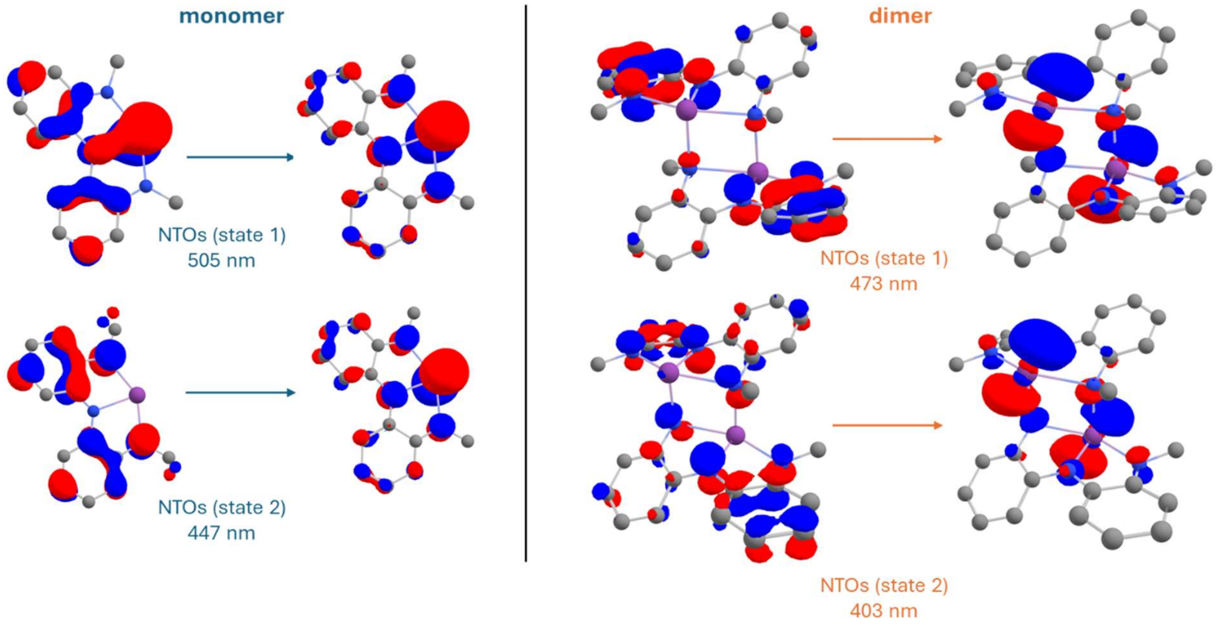

***Figure S98**. Calculated (ωb97xd/def2tzvp) natural transition orbitals (NTOs) involved in the two lowest energy excitations in **2a'** (left) and **2a** (right).*

6.4 Calculated frontier MOs for **2a** and **2b**

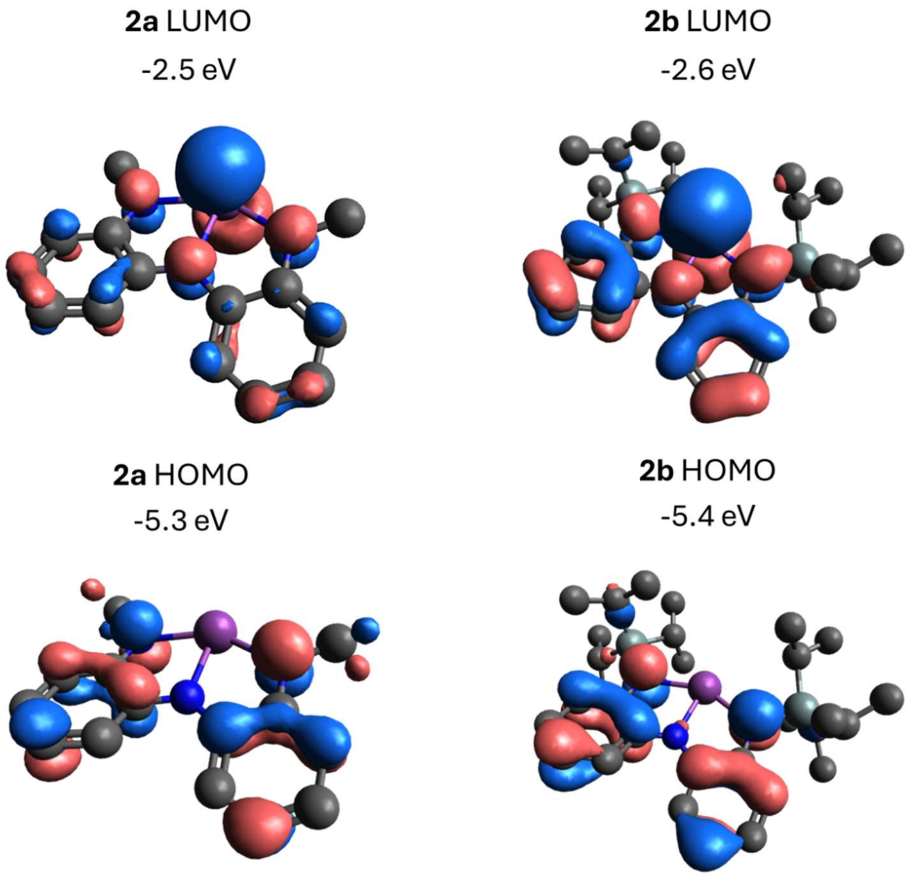

Figure S99. *Views of the calculated (pbe0/def2-QZPV+D3BJ) frontier MOs for **2a** and **2b**.*

6.5 Cartesian coordinates of calculated structures

2a monomer (r2SCAN-3c)

Bi	4.86602	10.35979	6.09083
N	4.57103	8.74031	4.58581
N	3.68232	8.69021	7.08047
N	5.86564	11.01062	4.15570
C	3.24702	8.76158	8.45983
H	3.74378	8.01135	9.09434
H	2.15989	8.61917	8.55554
H	3.48323	9.75342	8.86444
C	6.54699	12.28180	4.02519
H	6.03786	12.95874	3.32183
H	7.58591	12.15803	3.68365
H	6.58178	12.77733	5.00321
C	5.62434	10.25597	3.06866

C	6.01230	10.59739	1.75053
H	6.61599	11.48350	1.58668
C	5.60414	9.83636	0.67566
H	5.90220	10.12074	-0.32944
C	4.77000	8.72725	0.87213
H	4.38419	8.17695	0.01965
C	4.41248	8.33952	2.14972
H	3.71906	7.51854	2.27882
C	4.87710	9.04211	3.27757
C	3.57578	7.53789	6.39475
C	3.03207	6.33918	6.91629
H	2.59135	6.34237	7.90736
C	3.08704	5.16650	6.19317
H	2.67477	4.25423	6.61476
C	3.71301	5.13894	4.93938
H	3.82678	4.19984	4.40688
C	4.21142	6.30122	4.38166
H	4.74526	6.25012	3.44150
C	4.10008	7.53355	5.05288

2a' dimer (r2SCAN-3c)

Bi	4.80897	10.29348	6.18899
N	4.65848	8.70964	4.65931
N	3.49929	8.73171	7.04348
N	6.25629	10.85607	4.24762
C	2.98965	8.79875	8.39951
H	3.51696	8.11834	9.08925
H	1.91859	8.54832	8.44074
H	3.10477	9.81833	8.78653
C	6.39967	12.31059	4.05833
H	5.49400	12.71866	3.58845
H	7.26121	12.58625	3.43695
H	6.54766	12.79101	5.03137
C	5.79433	10.15344	3.10874
C	6.09526	10.53971	1.80088
H	6.75319	11.38781	1.63570
C	5.55589	9.86447	0.71083
H	5.80730	10.17450	-0.29859
C	4.67465	8.81171	0.93159
H	4.20963	8.30227	0.09261
C	4.36063	8.41177	2.22456
H	3.62647	7.63013	2.37851
C	4.94184	9.03810	3.33794
C	3.50454	7.52421	6.38684
C	2.99645	6.33020	6.92065

H	2.52562	6.33676	7.89801
C	3.09165	5.13664	6.21345
H	2.68234	4.22594	6.64060
C	3.72807	5.10919	4.97727
H	3.84317	4.17380	4.43829
C	4.23591	6.28198	4.42500
H	4.77183	6.23752	3.48391
C	4.10644	7.50540	5.09113
Bi	8.30890	9.92511	5.02376
N	8.45933	11.50890	6.55352
N	9.61849	11.48697	4.16932
N	6.86151	9.36243	6.96523
C	10.12811	11.42003	2.81327
H	9.60077	12.10045	2.12359
H	11.19916	11.67050	2.77204
H	10.01303	10.40047	2.42621
C	6.71806	7.90792	7.15456
H	7.62370	7.49983	7.62448
H	5.85649	7.63233	7.77593
H	6.57006	7.42748	6.18154
C	7.32348	10.06507	8.10409
C	7.02253	9.67883	9.41195
H	6.36459	8.83075	9.57714
C	7.56189	10.35408	10.50200
H	7.31047	10.04407	11.51142
C	8.44315	11.40683	10.28124
H	8.90815	11.91628	11.12022
C	8.75718	11.80675	8.98826
H	9.49133	12.58840	8.83431
C	8.17599	11.18041	7.87488
C	9.61322	12.69445	4.82602
C	10.12125	13.88850	4.29226
H	10.59206	13.88200	3.31489
C	10.02602	15.08203	4.99951
H	10.43529	15.99276	4.57239
C	9.38962	15.10940	6.23570
H	9.27450	16.04475	6.77473
C	8.88183	13.93656	6.78793
H	8.34593	13.98096	7.72903
C	9.01134	12.71318	6.12174

6.6 Description of **2a** Bonding as a Dimer

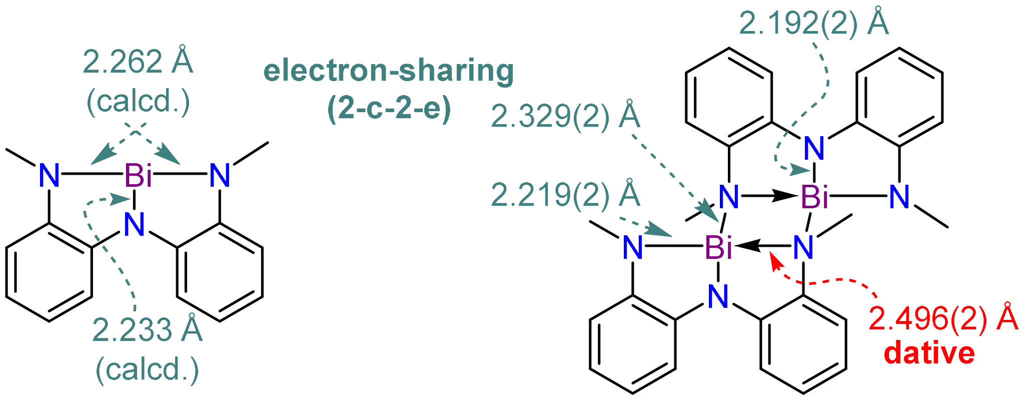

Figure S100. *Calculated bond lengths in 2a and experimental values for 2a' along with assignment as being electron-sharing (i.e. 2-centre-2-electron) bonds (teal) or dative bonds (red). These interpretations of the bond length are in line with orbital analyses of monomers vs. dimers reported previously.*[20]

7 References

1. W. Clegg, N. A. Compton, R. J. Errington, G. A. Fisher, M. E. Green, D. C. Hockless and N. C. Norman, *Inorg. Chem.*, **1991**, *30*, 4680-4682.

2. M. B. Kindervater, K. M. Marczenko, U. Werner-Zwanziger and S. S. Chitnis, *Angew. Chem. Int. Ed.*, **2019**, *58*, 7850-7855.

3. W. Zhao, S. M. McCarthy, T. Y. Lai, H. P. Yennawar and A. T. Radosevich, *J. Am. Chem. Soc.*, **2014**, *136*, 17634-17644.

4. R. E. H. Kuveke, L. Barwise, Y. van Ingen, K. Vashisth, N. Roberts, S. S. Chitnis, J. L. Dutton, C. D. Martin and R. L. Melen, *ACS Cent. Sci.*, **2022**, 8, 855-863.

5. APEX 4 V2022.10-1 (Bruker, **2022**) Bruker AXS Inc., Madison, Wisconsin, USA.

6. SAINT (Bruker, **2016**) Bruker AXS Inc., Madison, Wisconsin, USA.

7. SADABS (Bruker, **2016**) Bruker AXS Inc., Madison, Wisconsin, USA.

8. Sheldrick, G.M. (**2015**) *Acta Cryst.*, A71, 3-8.

9. Sheldrick, G.M. (**2015**) *Acta Cryst.*, C71, 3-8.

10. O. V. Dolomanov, L. J. Bourhis, R. J. Gildea, J. A. K. Howard and H. Puschmann, *J. Appl. Crystallogr.*, **2009**, 42, 339-341.

11. A. K. Tomov, V. C. Gibson, G. J. P. Britovsek, R. J. Long, M. van Meurs, D. J. Jones, K. P. Tellmann, J. J. Chirinos, *Organometallics*, **2009**, *28*, 7033-7040.

12. a) I. Kumar, P. Bhattacharya and K. H. Whitmire, *J. Organomet. Chem.*, **2015**, *794*, 153-167. b) F. He, Z. He, J. Xie and Y. Li, *Am. J. Analyt. Chem.*, **2014**, *5,* 1142-1150.

13. Y. Vonhausen, F. Würthner *Chem. Eur. J.* **2023**, *29*, e202300359.

14. L. Falivene, Z. Cao, A. Petta, L. Serra, A. Poater, R. Oliva, V. Scarano, L. Cavallo, *Nat. Chem.* **2019**, *11*, 872–879.

15. A. Poater, B. Cosenza, A., Correa, S. Giudice, F. Ragone, V. Scarano, L. Cavallo, *Eur. J. Inorg. Chem.*, **2009**, 1759–1766.

16. A. Poater, F. Ragone, S. Giudice, C. Costabile, R. Dorta, S. P. Nolan, L. Cavallo, *Organometallics*, **2008**, *27*, 2679–2681.

17. A. Poater, F. Ragone, R. Mariz, R. Dorta, L. Cavallo, *Chem. Eur. J.,* **2010**, *16*, 14348–14353.

18. F. Neese, *WIREs Comput. Mol. Sci.*, **2012**, *2*, 73-78.

19. S. Grimme, A. Hansen, S. Ehlert and J.-M. Mewes, *J. Chem. Phys.*, **2021**, *154*, 064103.

20. K. M. Marczenko, J. A. Zurakowski, M. B. Kindervater, S. Jee, T. Hynes, N. Roberts, S. Park, U. Werner-Zwanziger, M. Lumsden, D. N. Langelaan, S. S. Chitnis*, *Chem. Eur. J.*, **2019**, *25*, 16414-16424.
